# Trimerization and cyclization of reactive P-functionalities confined within OCO pincers†

**DOI:** 10.1039/d1ra05926b

**Published:** 2021-08-25

**Authors:** Beatrice L. Chinen, Jakub Hyvl, Daniel F. Brayton, Matthew M. Riek, Wesley Y. Yoshida, Timothy W. Chapp, Arnold L. Rheingold, Matthew F. Cain

**Affiliations:** Department of Chemistry, University of Hawai'i at Mānoa 2545 McCarthy Mall Honolulu HI 96822 USA mfcain@hawaii.edu; Department of Chemistry, Allegheny College 520 N. Main Street Meadville PA 16335 USA; Department of Chemistry, University of California 9500 Gilman Drive, La Jolla San Diego California 92093 USA

## Abstract

In order to stabilize a 10–P–3 species with *C*_2v_ symmetry and two lone pairs on the central phosphorus atom, a specialized ligand is required. Using an NCN pincer, previous efforts to enforce this planarized geometry at P resulted in the formation of a *C*_s_-symmetric, 10π-electron benzazaphosphole that existed as a dynamic “bell-clapper” in solution. Here, OCO pincers 1 and 2 were synthesized, operating under the hypothesis that the more electron-withdrawing oxygen donors would better stabilize the 3-center, 4-electron O–P–O bond of the 10–P–3 target and the sp^3^-hybridized benzylic carbon atoms would prevent the formation of aromatic P-heterocycles. However, subjecting 1 to a metalation/phosphination/reduction sequence afforded cyclotriphosphane 3, resulting from trimerization of the P(i) center unbound by its oxygen donors. Pincer 2 featuring four benzylic CF_3_ groups was expected to strengthen the O–P–O bond of the target, but after metal–halogen exchange and quenching with PCl_3_, unexpected cyclization with loss of CH_3_Cl was observed to give monochlorinated 5. Treatment of 5 with (*p*-CH_3_)C_6_H_4_MgBr generated crystalline P-(*p*-Tol) derivative 6, which was characterized by NMR spectroscopy, elemental analysis, and X-ray crystallography. The complex ^19^F NMR spectra of 5 and 6 observed experimentally, were reproduced by simulations with MestreNova.

## Abstract 2

Attempted synthesis of OCO-supported 10–P–3 species led to trimerization or cyclization.

## Graphical abstract

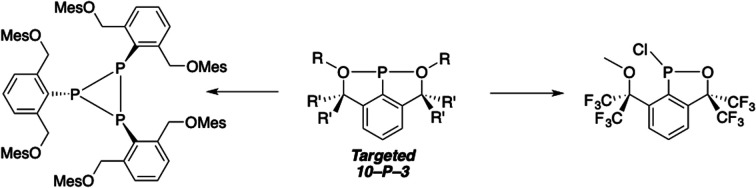

## Introduction

**Fig. 1:**
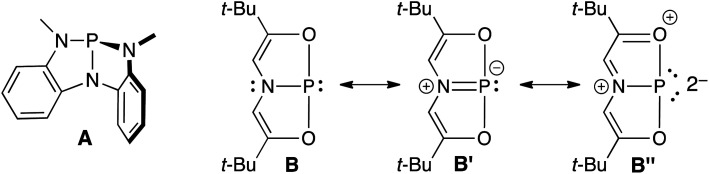
Structures of geometrically distorted A and B with contributing resonance structures B′ and B′′.

Transition metal (TM) catalysis^1^ has revolutionized the chemical industry, enabling the conversion of cheap feedstocks into valuable products for pharmaceuticals, polymers, and other specialty chemicals.^2^ However, some of the most commonly employed metals like Ru,^3^ Rh,^4^ Ir,^5^ Pd,^6^ and Pt^7^ are scarce, while the supporting ligands are often phosphines,^8^ N-heterocyclic carbenes (NHCs),^9^ and/or other elaborate scaffolds including chiral diols,^10^ functionalized cyclopentadienyl ligands (*ansa*-metallocenes),^11^ and rare and/or non-naturally occurring amines.^12^ These metal–ligand platforms are both expensive and toxic, resulting in a delicate balance between the benefits of chemical synthesis and its harmful effects to the environment and human health. An environmentally friendly and sustainable TM alternative would be to use a more benign and earth-abundant Main Group (MG) element such as phosphorus as the active center. Yet, unlike TMs that have closely spaced HOMO–LUMO gaps, MG compounds feature orbitals that are far apart energetically, which limits their ability to engage in TM-type reactivity like oxidative addition, insertion, and reductive elimination.^13^ Fortunately, by distorting phosphines away from their classic three-fold symmetry, their frontier orbitals can become energetically accessible.^14^ For example, *C*_s_-symmetric phosphorus triamide A can promote oxidative addition of alcohols and amines.^15–17^ Related *C*_2v_-symmetric B,^18^ describable by numerous resonance structures including B′ and B′′ due to extensive conjugation within the ONO ligand^19^ will oxidatively add H_2_ from H_3_N–BH_3_ and transfer that hydrogen equivalent to azobenzene in a catalytic fashion, producing hydrazines (Fig. 1).^20^

**Fig. 2:**
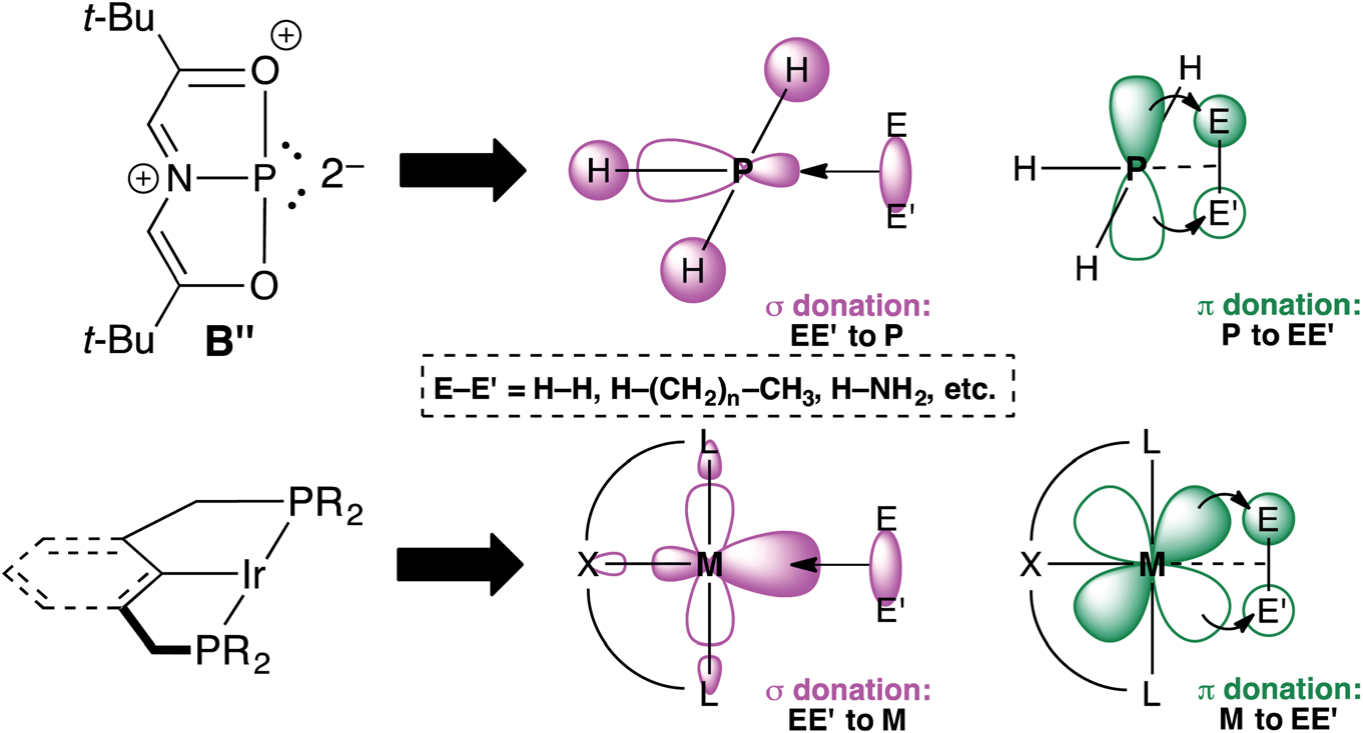
Simplified frontier orbital comparison between B′′ and an Ir(PCP) pincer fragment.

Resonance structure B′′ is a T-shaped 10–P–3 species,^21^ which contains a frontier orbital environment remarkably similar to a *C*_2v_-symmetric, d^8^ ML_3_ TM complex like the Ir(PCP) pincer fragment with a low-lying σ acceptor orbital and a higher energy lone pair with π-symmetry,^22^ capable of engaging in backbonding to an incoming substrate (Fig. 2). In the case of Ir(PCP) pincers,^23^ this results in the oxidative addition of dihydrogen,^24^ alkanes,^25^ and ammonia.^26^ However, B does not add H_2_ because the high-energy p-lone pair necessary for backbonding is delocalized into the ligand scaffold.^27^ Therefore, we speculated that replacing the tridentate ONO ligand with a traditional pincer featuring a central aryl donor would prevent π-delocalization, rendering the p lone pair available for backbonding to small molecules.

**Scheme 1:**
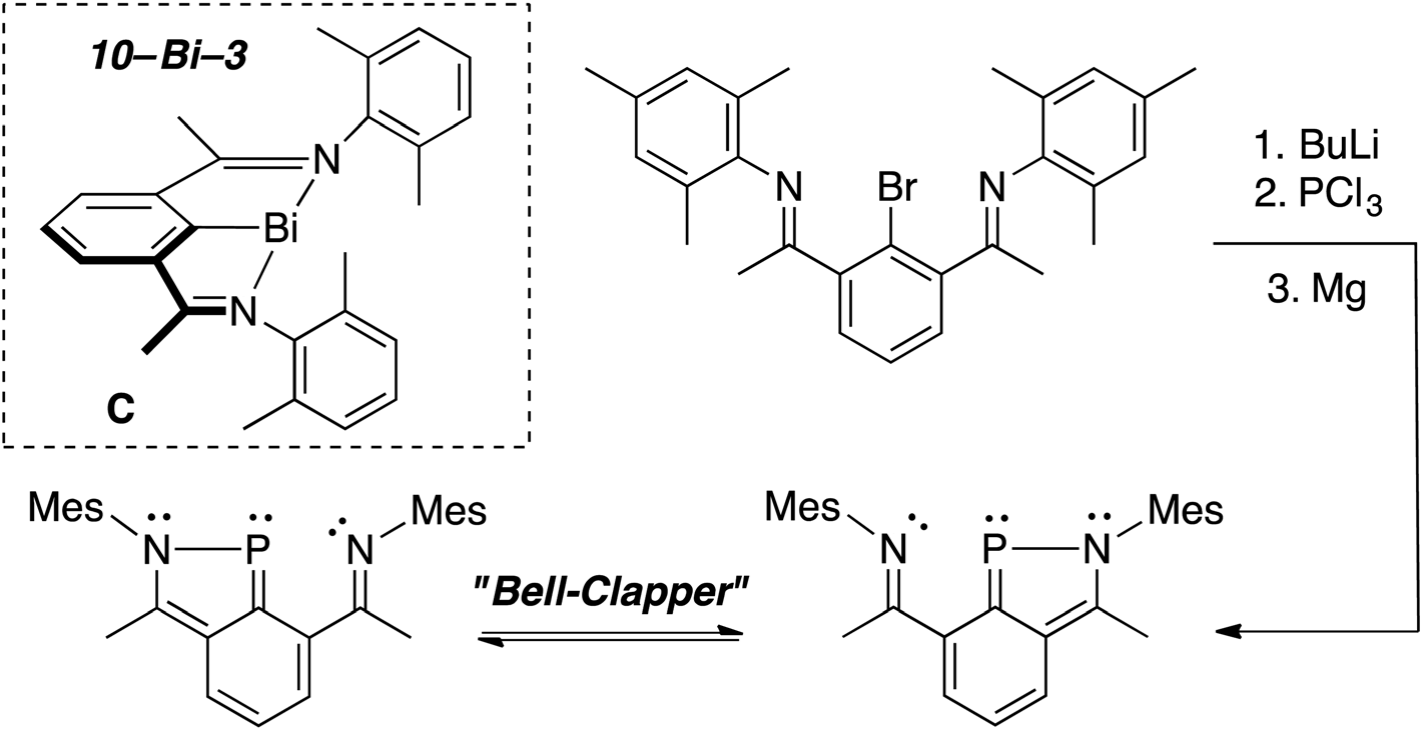
Synthesis of a benzazaphosphole “bell-clapper” with 10–Bi–3 species C shown in the inset.

In 2010,^28^ the Dostál group demonstrated that heavier, *C*_2v_-symmetric 10–Bi–3 species C (Scheme 1, inset) could be stabilized within an NCN pincer and similar analogues could perform oxidative addition of the weak bonds of diphenyldichalcogenides^29^ (ex. PhS–SPh, S–S bond = 55 kcal mol^−1^).^30^ This led us to attempt to stabilize a 10–P–3 species within a related NCN scaffold with the hope that substrates with stronger bonds, like those present in dihydrogen (H–H bond = 104 kcal mol^−1^),^31^ could be broken at P. Yet, unlike the Bi analogue, reduction of the P(iii) intermediate did not afford the desired 10–P–3 species, but rather a *C*_s_-symmetric 10π-electron benzazaphosphole with a tethered imine arm, which existed as a dynamic “bell-clapper” in solution (Scheme 1).^32^

**Chart 1:**
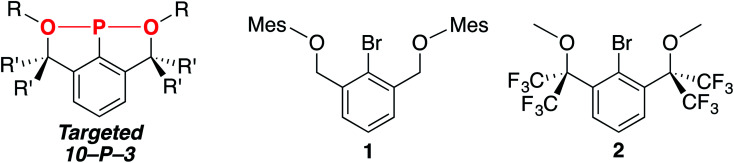
The targeted 10–P–3 species and brominated OCO pincers 1 and 2.

In order to access the targeted 10–P–3 species, we hypothesized that strengthening the 3-center, 4-electron bond between the axial donors and phosphorus (indicated in red, Chart 1, left) could be accomplished using more electron-withdrawing oxygen atoms.^19^ Additionally, sp^3^-hybridized benzylic carbons would be employed on the pincer arms to prevent the formation of aromatic P-heterocycles.^33^ Guided by these ligand design principles, brominated OCO pincers 1 (ref. 34) and 2 were synthesized (Chart 1, right), and we report here on the unexpected trimerization and cyclization chemistry encountered when installing the P-functionality within these scaffolds.

## Results and discussion

### Synthesis of 1 and the formation of cyclotriphosphane 3*via* reduction

**Scheme 2:**
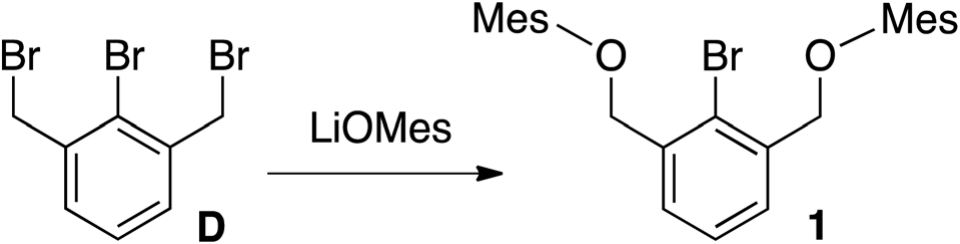
Synthesis of 1.

Tribromide D^35^ was treated with LiOMes (Mes = 2,4,6-trimethylbenzene), generated *in situ* from BuLi and 2,4,6-trimethylphenol, affording 1 in 64% yield (Scheme 2).

**Fig. 3:**
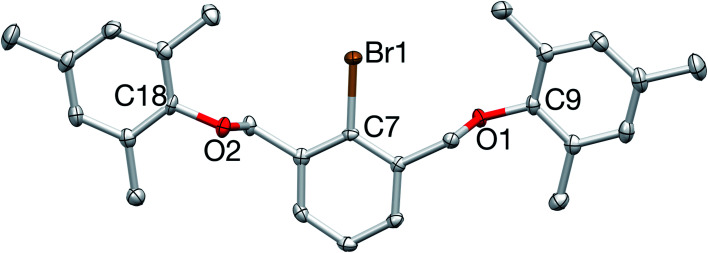
X-ray crystal structure of 1. All bond lengths (Å) and angles (deg) can be found in the ESI.†

Monobrominated OCO pincer 1 was readily identified by ^1^H NMR spectroscopy with its downfield shifted benzylic signals (CDCl_3_: *δ* 4.93; relative to D, CDCl_3_: *δ* 4.55), integrating in a 4 : 2 : 1 ratio with the diagnostic doublet/triplet pattern of the central aryl protons. The proligand was further characterized by ^13^C{^1^H} NMR spectroscopy and elemental analysis, and its structure was unequivocally confirmed by X-ray crystallography (Fig. 3).

**Scheme 3:**
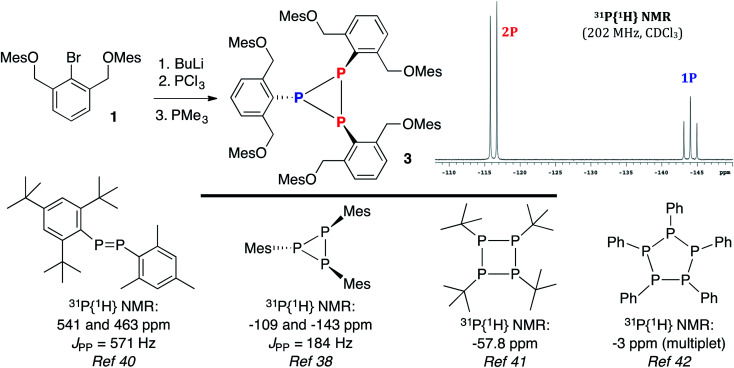
Synthesis of 3 and a selected view of its ^31^P{^1^H} NMR spectrum (top) and related (RP)_*n*_ oligomers (bottom).

Based on literature precedent with heavier pnictogens,^28,36^ it was anticipated that the bromide in 1 could be substituted by a PCl_2_ unit *via* a lithium–halogen exchange/phosphination sequence. Reduction of the intermediate dichlorophosphine would then produce the desired 10–P–3 species. However, treatment of 1 with BuLi, followed by quenching with PCl_3_ and reduction^37^ with PMe_3_ did not afford the target, but rather cyclotriphosphane 3 in 56% yield (Scheme 3). The ^31^P{^1^H} NMR spectrum of 3 (inset) contained upfield shifted resonances at −116 and −144 ppm with a *J*_PP_ = 186 Hz, consistent with a solution structure in which two P atoms are spectroscopically distinct from a third.^38^ This NMR signature is in line with other (PR)_3_ species like (PIs)_3_ (Is = 2,4,6-tri-isopropylbenzene) and (PMes)_3_,^38^ but not diphosphenes like Mes*P=PMes* (Mes* = 2,4,6-tri-*tert*-butylbenzene), which feature *bona fide* P=P double bonds (2.034(2) Å),^39^ downfield shifted ^31^P{^1^H} NMR signals (*δ* = 493),^40^ and if unsymmetrical,^38,40^ large *J*_PP_ couplings approaching 600 Hz (Mes*P=PMes, *J*_PP_ = 571 Hz). Other higher order monocyclophosphanes like tetrameric [P(*t*-Bu)]_4_ are singlets (^31^P NMR: *δ* −57.8),^41^ while (PR)_5_ pentamers (R = Ph) are complex multiplets (^31^P NMR: *δ* −3)^42^ (Scheme 3, bottom panel).

**Fig. 4:**
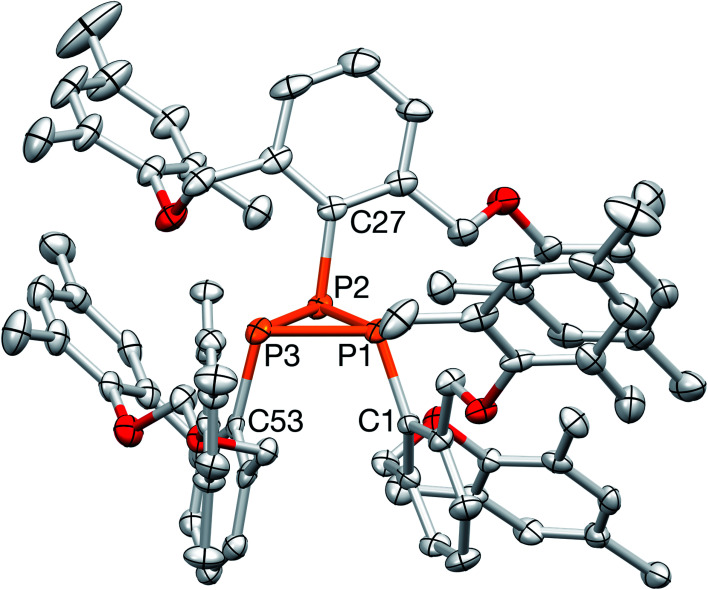
X-ray crystal structure of 3. Selected bond lengths (Å) and angles (deg): P_1_–P_2_ = 2.194(2), P_2_–P_3_ = 2.217(2), P_1_–P_3_ = 2.237(2), P_1_–C_1_ = 1.839(5), P_2_–C_27_ = 1.853(5), P_3_–C_53_ = 1.850(5), P_3_–P_2_–P_1_ = 60.95(7), P_1_–P_3_–P_2_ = 59.02(7), P_3_–P_1_–P_2_ = 60.03(7).

The structure of cyclotriphosphane 3 was further corroborated by ^1^H and ^13^C{^1^H} NMR spectroscopy. Specifically, this “2-Down, 1-Up”-type (referring to the organic substituents on P) structure was readily apparent as two distinct aryl *O*-Mes singlets were observed in the ^1^H NMR spectrum (CDCl_3_) at 6.71 and 6.62 ppm in an 8 : 4 ratio with all the remaining resonances paired (although some broadened) in a similar 2 : 1 fashion. In addition, the ^13^C{^1^H} NMR spectrum displayed two separate benzylic signals and four methyl signals, all consistent with the assignment of 3. Ultimately, the structure of 3, as proposed, was established by X-ray crystallography (Fig. 4). Structural characterization of cyclotriphosphanes is rare,^43^ but the bond lengths and angles of 3 are quite similar to [P(*t*-Bu)]_3_ and [PCH(SiMe_3_)_2_]_3_.^44,45^ In particular, the P–P bonds measure 2.217(2), 2.194(2), and 2.237(2) Å, respectively with PPP angles of 60.95(7), 59.02(7), and 60.03(7) deg, indicative of P–P single bonds and unhybridized P-centers confined into a small ring system.^43^ The P–C bonds in 3 (avg = 1.847 Å) are slightly shorter than observed with [P(*t*-Bu)]_3_ and [PCH(SiMe_3_)_2_]_3_, which may be due to the presence of an sp^2^-hybridized^46^*C*-substituent with considerably less bulk and more flexibility than the *t*-Bu and CH(SiMe_3_)_2_ groups. In fact, in comparison with cyclotriphosphanes [P(*t*-Bu)]_3_, [PCH(SiMe_3_)_2_]_3_, (PIs)_3_, and (PMes)_3_, it is somewhat surprising that 3 adopts a related structure because normally, as steric bulk decreases, the size of the oligomeric fragment increases (note: (PMes)_3_*versus* (PPh)_5_); however, here, many close contacts (under 4 Å)^47^ between the aryl rings of the OMes units may impart added stability to the cyclotriphosphane structure.

Regardless, the structure of 3 suggested that the axial 3-center, 4-electron bond of the targeted 10–P–3 species was still the weak point. In fact, cyclotriphosphane 3 is formally the result of trimerization^48^ of an OCO-supported P(i) intermediate after the *O*-donors rotated away from the electron-deficient phosphorus center. In order to elucidate any mechanistic details, the phosphination and reduction steps (shown in Scheme 3) were monitored by ^31^P{^1^H} NMR spectroscopy, but did not reveal the generation of RP=PMe_3_ (R = OCO pincer), a proposed precursor to the formation of cyclotriphosphanes such as (PIs)_3_, nor did a solution of 3, PMe_3_, and benzaldehyde produce any phosphaalkenes.^48^

### Synthesis of 2 and cyclization of PCl_2_-functionalized 4

**Scheme 4:**
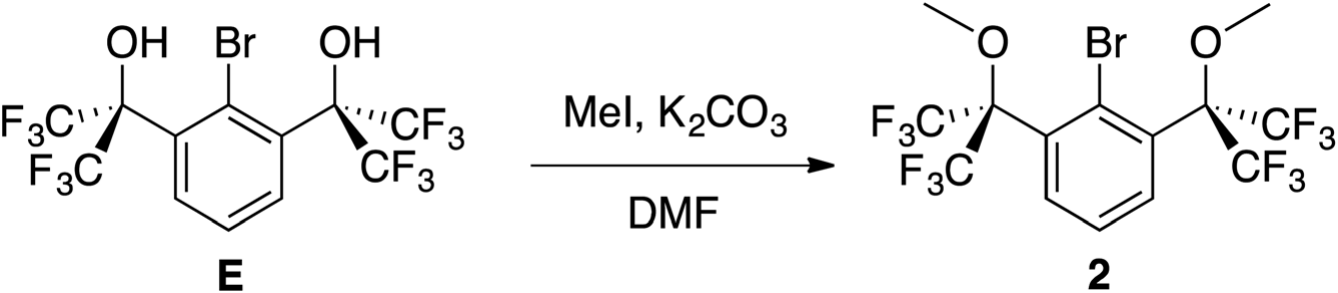
Synthesis of 2.

In order to further strengthen the 3-center, 4-electron O–P–O bond of the potential 10–P–3 species, the electronegativity of the *O*-donors was increased using benzylic CF_3_ groups. To this end, diol E,^49^ now accessible in a single step^50^ from dimethyl 2-bromoisophthalate and TMSCF_3_ and previously used to stabilize numerous hypervalent MG species^51^ including 10–Br–3 (ref. 52) and 12–I–5,^49^ was selected as the building block to brominated OCO pincer 2. After isolation of E in multi-gram quantities,^53^ dimethylation with MeI/K_2_CO_3_ in DMF resulted in the isolation of 2 in 78% yield (Scheme 4).

**Fig. 5:**
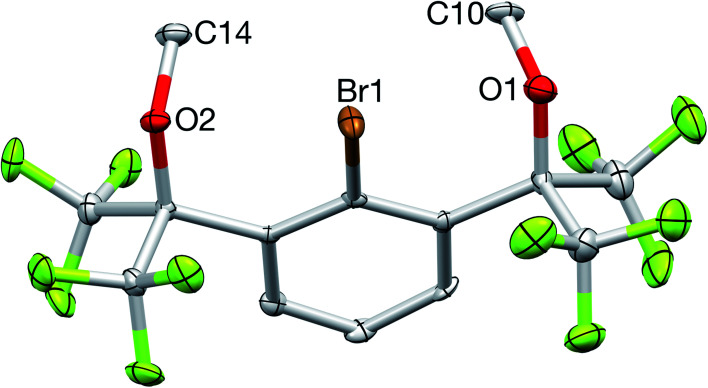
X-ray crystal structure of 2. All bond lengths (Å) and angles (deg) can be found in the ESI.†

The ^1^H NMR spectrum (CDCl_3_) featured a prominent singlet for the OMe groups at 3.52 ppm, which integrated in a 6 : 2 : 1 ratio with the remaining central aryl protons, while the ^19^F NMR spectrum contained a single resonance at −66.9 ppm, all consistent with the expected *C*_2v_ symmetry of 2. Additional characterization by ^13^C{^1^H} NMR spectroscopy, elemental analysis, and X-ray crystallography confirmed its structure (Fig. 5).

**Scheme 5:**
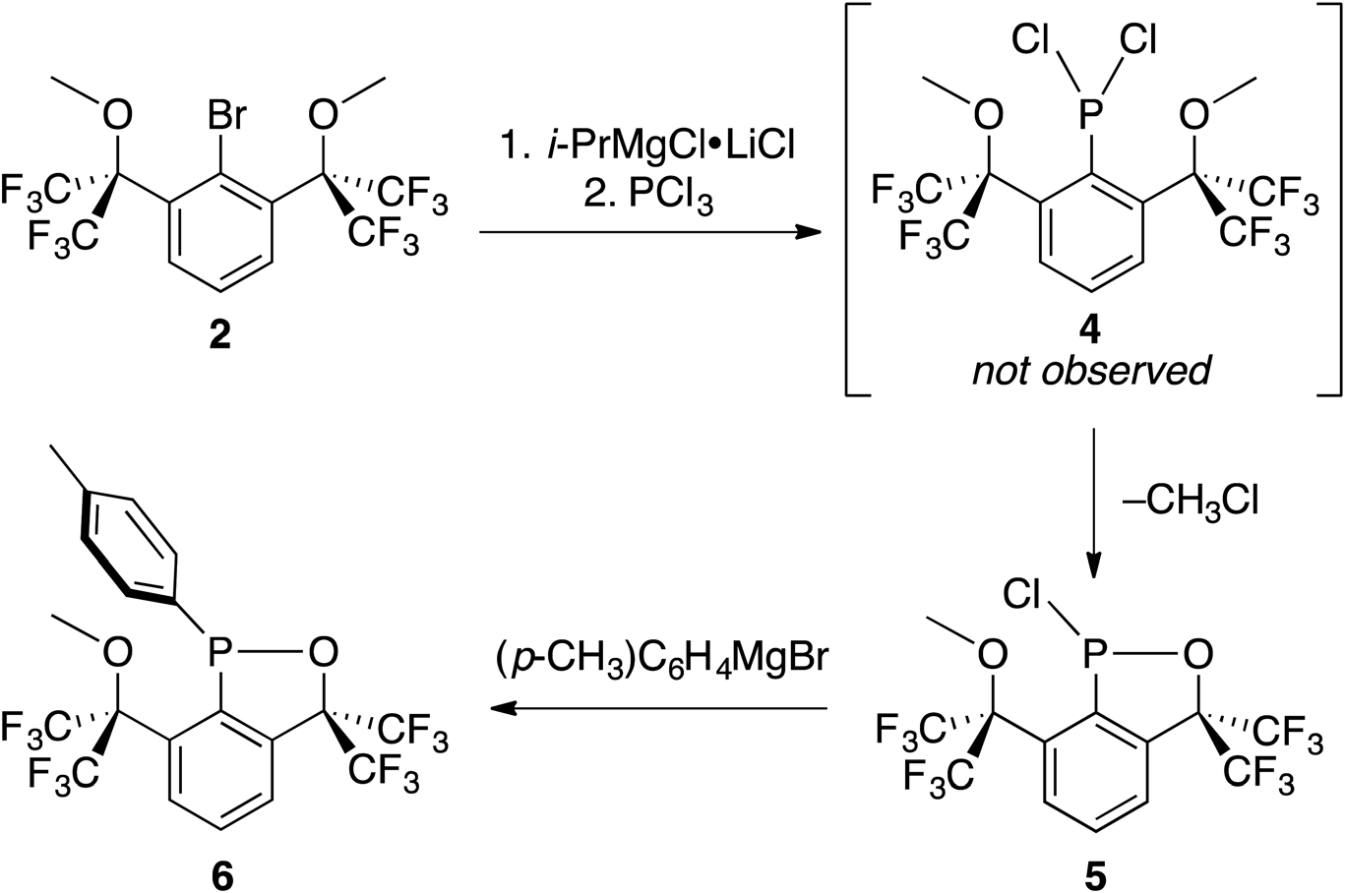
Intramolecular cyclization of 4 to 5 and synthesis of crystalline derivative 6.

Cognizant that lithium–halogen exchange using BuLi in the presence of fluorine atoms may be complicated by LiF formation^54^ and that magnesium–halogen exchange is faster with electron-withdrawing substrates and more functional group tolerant,^55^2 was exposed to i-PrMgCl·LiCl,^56^ resulting in smooth *in situ* conversion to the Grignard reagent, which was subsequently quenched with a precooled solution (−35 °C) of PCl_3_ in THF (Scheme 5).

**Fig. 6:**
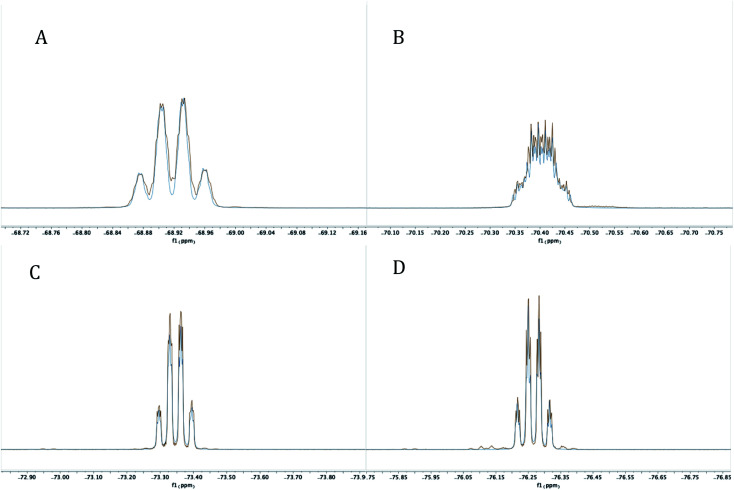
Experimental (red) and simulated (blue) ^19^F NMR signals at −68.9237 (A), −70.4093 (B), −73.3518 (C) and −76.2722 (D) for 5 at 282 MHz.

The reaction mixture was then analyzed by ^31^P{^1^H} NMR spectroscopy (THF), revealing the presence of some unreacted PCl_3_ (218 ppm), (i-Pr)PCl_2_ (202 ppm), and an unidentified product (171 ppm). The ^19^F NMR spectrum (C_6_D_6_) displayed four complex multiplet CF_3_ signals at −68.9, −70.4, −73.4, and −76.3 ppm, demonstrating that the unidentified product no longer contained *C*_2v_ symmetry and was not PCl_2_-substituted 4. Instead, we suspected an intramolecular reaction occurred between the highly electrophilic PCl_2_ functionality and one of the *O*-donors, resulting in cyclization and the loss of chloride, which subsequently dealkylated^57^ the O–Me unit affording monochlorinated 5. Using MestreNova,^58^ simulated ^19^F NMR signals of 5 that closely matched the experimental spectrum were generated (Fig. 6), revealing the presence of both second order and long range coupling between the diastereotopic CF_3_ groups, the heterocyclic P atom, and the aryl protons (see ESI† for details).

**Fig. 7:**
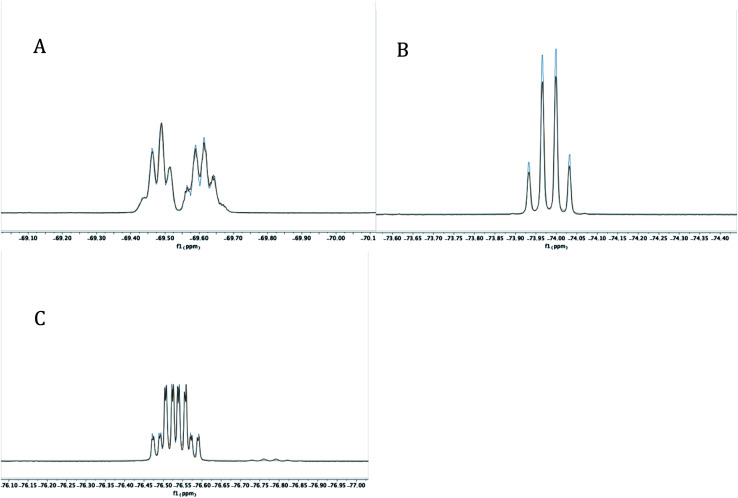
Experimental (red) and simulated (blue) ^19^F NMR signals at −69.4819 and −69.6176 (A), −73.9876 (B), and −76.5368 (C) for 6 at 282 MHz.

Experimentally, the cyclization to 5 was confirmed by synthesizing a more crystalline derivative *via* nucleophilic substitution. Specifically, a solution of 5 in THF at 0 °C was treated with (*p*-CH_3_)C_6_H_4_MgBr, leading to the isolation of 6 as large off-white crystals in 38% yield (from 2, Scheme 5, above; see Fig. 9 for picture of crystals). The ^31^P{^1^H} NMR spectrum (C_6_D_6_) of 6 exhibited an apparent septet at 129.7 ppm (^4^*J*_PF_ = 6 Hz), while its ^19^F NMR spectrum, like 5, displayed four distinct resonances for the diastereotopic CF_3_ groups at −69.5, −69.6, −74.0, and −76.5 ppm. These complex ^19^F NMR signals could also be reproduced with MestreNova (Fig. 7, see ESI† for details).

**Fig. 8:**
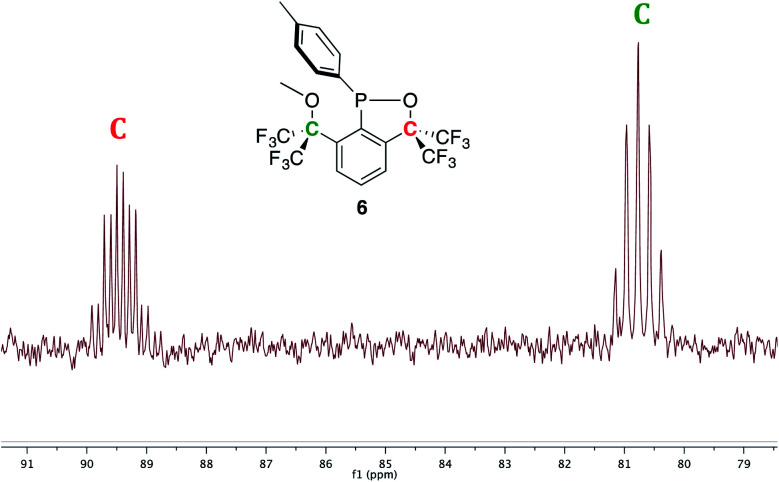
A selected view of the ^13^C{^1^H} NMR spectrum (C_6_D_6_) of 6 displaying the quaternary carbons.

^1^H NMR spectroscopy (C_6_D_6_) further corroborated the structure of 6 as the aromatic signals associated with the *p*-tolyl group and the three inequivalent protons of the central aryl unit of the OCO pincer integrated in a 2 : 2 : 1 : 1 : 1 ratio; in addition, two distinct methyl signals (OMe, 3.11 ppm and *p*-Me, 1.92 ppm) were observed. The ^13^C{^1^H} NMR spectrum also highlighted the inequivalency of the CF_3_ groups with four overlapping signals (see ESI† for zoomed in NMR spectrum): two quartets (*J*_CF_ ∼ 290 Hz) combined with two quartets of doublets (*J*_CF_ ∼ 290 Hz and *J*_CP_ ∼ 9 or 2 Hz, respectively) in the 122–123 ppm range, while the two quaternary carbons *C*(CF_3_)_2_ resonated as a septet (80.8 ppm, *J*_CF_ = 28 Hz) and a septet of doublets (89.5 ppm, *J*_CF_ = 31.5 Hz, *J*_CP_ = 16.5 Hz, Fig. 8).

**Fig. 9:**
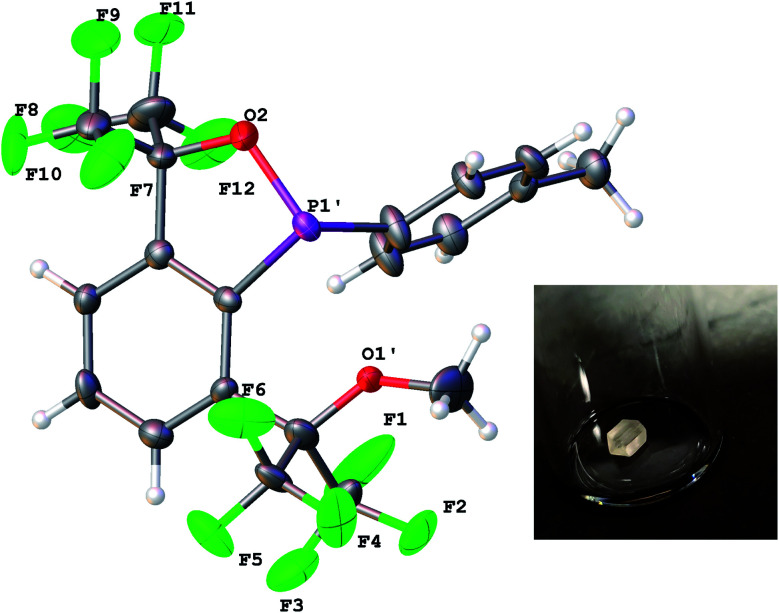
X-ray crystal structure of 6. All bond lengths (Å) and angles (deg) can be found in the ESI.† A picture of one of the large blocks is shown in the inset.

Ultimately, although disordered across a pseudo mirror plane, X-ray crystallography established the structure of 6 with its bulk purity verified by elemental analysis (Fig. 9).

Unfortunately though, like 1, fluorinated OCO pincer 2 also failed to deliver access to the 10–P–3 target. Here, the increased electron-withdrawing power of four benzylic CF_3_ groups resulted in, after a metal–halogen exchange/phosphination sequence, highly reactive dichlorophosphine 4, which *via* an unexpected cyclization^57^ to 5, partially disassembled the pincer framework before reduction could be attempted.

## Conclusions and future directions

Undoubtedly, the hypothesis (*vide supra*) that the formation of 10–P–3 species over benzazaphosphole “bell-clappers” would be favored by switching from NCN pincers to more electron withdrawing OCO pincers with sp^3^-hybridized benzylic carbon atoms was overly simplistic. As the phrase goes, “hindsight is 20/20” and here, the biggest oversight was expecting a P-center to adopt a high-energy planarized geometry when lower barrier pathways to phosphorus maintaining its preferred pyramidal geometry exist. For example, 10–P–3 species like B′′ (Fig. 1) and the target (Chart 1, left) can be considered “internally-solvated phosphinidenes”^19^ that are supported by a 3-center, 4-electron O–P–O bond. However, B′′ features a conjugated ONO ligand that locks the *O*-donors into place, while C_Ar_–C_sp_^3^ or C_sp_^3^–O bond rotations within OCO pincer 1 expose the reactive P(i) center, resulting in oligomerization chemistry,^43,48,59^ a known route by which phosphorus preserves its pyramidal structure and is exemplified by the formation of trimer 3. To prevent this, we aimed to strengthen the 3-center, 4-electron bond using more electron-withdrawing *O*-donors, but neglected how the enhanced electrophilicity of PCl_2_-substituted 4 can make the ligand framework susceptible to nucleophilic attack, in this case, cyclization to 5 (and its functionalization to 6). Guided by these lessons, a pincer framework that is rigid featuring electron-withdrawing *O*-donors, but lacking sp^3^-hybridized^57^ carbon atoms prone to nucleophilic attack may provide the stabilization necessary to confine a P-center into a planarized 10–P–3 arrangement and will be investigated.

## Experimental section

### General experimental details

Unless otherwise specified, all reactions were performed under an atmosphere of nitrogen in an MBraun or Vacuum Atmospheres glovebox or using standard Schlenk techniques. All glassware was dried overnight in an oven at 140 °C prior to use. Solvents used in the glove box were purchased directly from chemical suppliers (Aldrich or Acros), pumped directly into the glove box, and stored over oven-activated 4 or 5 Å molecular sieves (Aldrich). Solvents used outside the glove box were purged with N_2_ for 30 min and stored over molecular sieves. TMSCF_3_ was dried by cryogenic transfer. ^1^H, ^13^C{^1^H}, ^19^F, and ^31^P{^1^H} NMR spectra were recorded on a Varian Mercury-300 (300/75/282/121 MHz), Varian Unity Inova-500 (500/126/470/202 MHz), or Agilent 600 DD2 (600/151/565/243 MHz) spectrometer at ambient temperature. Chemical shifts are reported in ppm downfield of tetramethylsilane using the solvent as internal standard (^1^H CDCl_3_ = 7.27 ppm, ^1^H C_6_D_6_ = 7.16 ppm, ^13^C CDCl_3_ = 77.16 ppm, ^13^C C_6_D_6_ = 128.06 ppm). Multiplicities are abbreviated as br (broad), s (singlet), d (doublet), t (triplet), q (quartet), or m (multiplet). Coupling constants (*J*) are reported in Hertz (Hz). Flash column chromatography was performed on silica gel (40–63 μm, SiliCycle). High-resolution mass spectrometry (HRMS) was recorded on an Agilent 6545 LC-MS Q-ToF spectrometer (NSF-1532310). Dimethyl 2-bromoisophthalate and [Me_4_N][F] were synthesized according to the published procedures.^60,61^ All other chemicals were used as received, unless otherwise noted.

### Synthesis of 1

In the glovebox, 2,4,6-trimethylphenol (3.00 g, 22.03 mmol, 2.5 equiv.) was dissolved in THF (100 mL), and a 1.6 M solution of *n*-BuLi in hexanes (13.0 mL, 20.8 mmol, 2.3 equiv.) was added dropwise at room temperature. The reaction mixture was stirred for 15 min. Subsequently, tribromide D (3.086 g, 9.0 mmol) was added to the stirred solution, and the reaction mixture was transferred to a Schlenk bomb equipped with a Teflon screw cap and heated at 100 °C for 1 day. The volatiles were then removed under reduced pressure, and the crude residue was extracted with a toluene : hexane mixture (1 : 1, 3 × 15 mL). The combined extracts were filtered through a Celite plug, and the filtrate was concentrated under vacuum then purified by column chromatography (silica gel, toluene : hexane 1 : 1, *R*_f_ = 0.63). The title product (2.64 g, 5.822 mmol) was obtained as white plates in 65% yield. X-Ray quality crystals were obtained by slow evaporation from a concentrated solution of 1 in *n*-hexanes.

Anal. Calcd for C_26_H_29_BrO_2_: C, 68.87; H, 6.45. Found: C, 68.77; H, 6.27. ^1^H NMR (500 MHz, CDCl_3_): *δ* 7.78 (d, *J* = 7.6 Hz, 2H, Ar), 7.51 (t, *J* = 7.6 Hz, 1H, Ar), 6.89 (s, 4H, Ar), 4.93 (s, 4H, CH_2_), 2.30 (s, 12H, Me), 2.29 (s, 6H, Me). ^13^C{^1^H} NMR (126 MHz, CDCl_3_): *δ* 153.5 (Ar), 137.8 (Ar), 133.6 (Ar), 130.9 (Ar), 129.6 (Ar), 127.9 (Ar), 127.7 (Ar), 121.6 (Ar), 73.3 (CH_2_), 20.9 (CH_3_), 16.4 (CH_3_).

### Synthesis of 2

Diol E (3.61 g, 7.38 mmol) and DMF (180 mL) were combined in a Schlenk flask with a stir bar. The solution was purged with N_2_ on the Schlenk line for 30 min, then K_2_CO_3_ (5.10 g, 36.9 mmol, 5 equiv.) was added with stirring under positive N_2_ pressure. Next, MeI (2.30 mL, 36.9 mmol, 5 equiv.) was injected *via* syringe, and the reaction mixture was stirred overnight, then quenched with NH_4_Cl (aq) and extracted with toluene (3 × 50 mL). The combined organic layers were washed with water, brine, dried over MgSO_4_, filtered, and the filtrate concentrated under vacuum, resulting in pale yellow solid 2 (2.97 g, 5.74 mmol, 78%). Recrystallization from acetonitrile (8 mL) at 4 °C afforded crystals suitable for X-ray diffraction.

Anal. Calcd for C_14_H_9_BrO_2_F_12_: C, 32.52; H, 1.75. Found: C, 32.71; H, 1.77. ^1^H NMR (500 MHz, CDCl_3_): *δ* 7.76 (d, *J* = 10 Hz, 2H, Ar), 7.51 (t, *J* = 10 Hz, 1H, Ar), 3.52 (s, 6H, Me). ^13^C{^1^H} NMR (151 MHz, CDCl_3_): *δ* 134.1 (Ar), 129.6 (Ar), 127.0 (Ar), 123.5 (Ar), 122.4 (q, *J* = 290 Hz, CF_3_), 86.5 (septet, *J* = 29 Hz, *C*(CF_3_)_2_), 54.8 (OMe). ^19^F NMR (471 MHz, CDCl_3_): *δ* −66.9 (s).

### Synthesis of 3

OCO-supported aryl bromide 1 (1.134 g, 2.50 mmol) was loaded into a Schlenk flask, dissolved in Et_2_O (60 mL), taken outside of the glovebox, and cooled to −78 °C. A 1.6 M solution of *n*-BuLi in hexanes (1.7 mL, 2.72 mmol, 1.1 equiv.) was injected (under N_2_), and the reaction mixture was stirred at −78 °C for 5 min. A second Schlenk flask containing a solution of PCl_3_ (550 mg, 4.01 mmol, 1.6 equiv.) in Et_2_O (5 mL) was transferred *via* cannula to the cooled reaction mixture. The reaction mixture was stirred at −78 °C for 10 min and then at room temperature (RT) for 1 h, leading to the precipitation of a white solid. The volatiles were removed under reduced pressure, the Schlenk flask was brought back into the glovebox, the residue was triturated with Et_2_O (10 mL), and the volatiles were again removed. The residue was dissolved in THF (20 mL), and PMe_3_ was added (485 mg, 6.375 mmol, 2.6 equiv.). The reaction mixture was stirred at RT for 1 day. The organic volatiles were removed under reduced pressure, and the residue was extracted with toluene (3 × 20 mL). The combined extracts were filtered through a Celite plug, the filtrate was concentrated to dryness, triturated with *n*-pentane (10 mL), and again concentrated to dryness under reduced pressure. The resulting residue was triturated with acetonitrile (10 mL) and stirred at RT for 1 h, generating a white precipitate that was collected by filtration and dried (565 mg, 0.466 mmol, 56%). X-Ray quality crystals of 3 were obtained by recrystallization from a solution of hot acetonitrile.

Anal. Calcd for C_78_H_87_O_6_P_3_: C, 77.20; H, 7.23. Found: C, 76.89; H, 7.14. ^31^P{^1^H} NMR (202 MHz, CDCl_3_): *δ* −116.2 (d, ^1^*J*_PP_ = 186 Hz), −144.0 (t, ^1^*J*_PP_ = 186 Hz). ^1^H NMR (500 MHz, CDCl_3_): *δ* 7.71 (d, *J* = 7.7 Hz, 2H, Ar^1^), 7.40 (t, *J* = 7.7 Hz, 1H, Ar^1^), 7.33–7.28 (m, 4H, Ar^2^), 7.28–7.22 (m, 2H, Ar^2^), 6.71 (s, 8H, Ar^2^), 6.62 (s, 4H, Ar^1^), 5.39 (s, 4H, CH_2_–Ar^1^), 4.71 (br, 8H, CH_2_–Ar^2^), 2.25 (s, 12H, Ar^1^), 2.20 (s, 6H, Ar^1^), 2.02 (s, 12H, Ar^2^), 1.95 (br, 24H, Ar^2^). ^13^C NMR (126 MHz, CDCl_3_): *δ* 153.46 (Ar), 153.44 (Ar), 143.5 (Ar), 143.4 (d, *J*_PC_ = 6.9 Hz, Ar), 133.6 (Ar), 133.0 (Ar), 132.9 (Ar), 130.7 (Ar), 130.5 (Ar), 130.4 (Ar), 129.6 (Ar), 129.4 (Ar), 129.0 (Ar), 128.4 (d, *J*_PC_ = 10.0 Hz, Ar), 127.9 (Ar), 126.4 (Ar), 73.2 (CH_2_), 72.8 (d, *J*_PC_ = 7.6 Hz, CH_2_), 20.9 (CH_3_), 16.7 (CH_3_), 16.4 (CH_3_), 16.3 (CH_3_).

### Synthesis of 6

Fluorinated aryl bromide 2 (500 mg, 0.967 mmol) was dissolved in 6 mL of THF in a vial with a stir bar inside the glovebox and (i-Pr)MgCl·LiCl was added dropwise *via* syringe (0.82 mL, 1.064 mmol, 1.1 equiv., 1.3 M in THF), resulting in a homogeneous, yellow reaction mixture. The reaction mixture was stirred for 1 h at room temperature (RT) then directly filtered into 2 mL of a pre-cooled solution (−35 °C, 1 h) of PCl_3_ (146 mg, 1.064 mmol, 1.1 equiv.) in THF. The solution was warmed to RT for 1 h then analyzed by ^31^P{^1^H} NMR spectroscopy, revealing the presence of unreacted PCl_3_ (218 ppm), i-PrPCl_2_ (202 ppm) and the chlorophosphine (170 ppm). The “intermediate” product mixture was then concentrated under vacuum to remove the volatile and unwanted phosphorus byproducts (PCl_3_ and i-PrPCl_2_), leaving a pale yellow residue that was dissolved in 6 mL of THF and transferred to a Schlenk bomb fitted with a screw-top Teflon cap. The bomb was taken outside of the glovebox, cooled to 0 °C, and (*p*-CH_3_)C_6_H_4_MgBr (1.06 mL, 1.064 mmol, 1.1 equiv., 1.0 M in THF) was injected *via* syringe under positive N_2_ pressure, affording a light orange reaction mixture, which was subsequently warmed to RT. The Schlenk bomb was resealed (under positive N_2_ pressure), brought back inside the glove box, and an aliquot was analyzed by ^31^P{^1^H} NMR spectroscopy displaying a prominent signal at 129 ppm with slight impurities at −2 and −8 ppm. The entire reaction mixture was concentrated under vacuum, extracted with pentane (3 × 50 mL), and filtered through a Kimwipe plug. The filtrate was concentrated under vacuum, dissolved in ether (2 mL) and cooled to −35 °C overnight, resulting in large white/colorless blocks suitable for X-ray diffraction (202 mg, 0.371 mmol, 38% yield).

Anal. Calcd for C_20_H_13_F_12_O_2_P: C, 44.14; H, 2.41. Found: C, 44.09; H, 2.41. ^1^H NMR (600 MHz, C_6_D_6_): *δ* 7.57 (br d, *J* = 8 Hz, 1H, Ar), 7.52 (d, *J* = 8 Hz, 1H, Ar), 7.10 (apparent t, *J* = 8 Hz, 2H, Ar), 6.88 (t, *J* = 8 Hz, 1H, Ar), 6.81 (d, *J* = 8 Hz, 2H, Ar), 3.11 (s, 3H, OMe), 1.92 (s, 3H, *p*-Me). ^13^C{^1^H} NMR (151 MHz, C_6_D_6_): *δ* 143.4 (d, *J* = 33 Hz, Ar), 141.2 (Ar), 137.8 (d, *J* = 41 Hz, Ar), 135.7 (d, *J* = 6 Hz, Ar), 133.0 (d, *J* = 5 Hz, Ar), 132.2 (d, *J* = 25 Hz, Ar), 131.1 (Ar), 130.0 (Ar), 129.0 (d, *J* = 8 Hz, Ar), 126.9 (Ar), four overlapping CF_3_ signals*: 123.1 (qd, *J* ∼ 290 and 9 Hz), 123.0 (q, *J* ∼ 290 Hz), 122.5 (q, *J* ∼ 290 Hz), and 122.4 (qd, *J* ∼ 290 and 3 Hz), 89.5 (septet of doublets, *J* = 31.5 and 16.5 Hz, *C*(CF_3_)_2_ in P-ring), 80.8 (septet, *J* = 28 Hz, *C*(CF_3_)_2_), 55.3 (OMe), 21.2 (Me). ^19^F NMR (282 MHz, C_6_D_6_):** *δ* −69.5 (m, *J*_FF_ ∼ 8 Hz, *J*_PF_ ∼ 2 Hz, *J*_HF_ ∼ 0.5–2 Hz), −69.6 (m, *J*_FF_ ∼ 8 Hz, *J*_PF_ ∼ 7 Hz, *J*_HF_ ∼ 0.5–2.5 Hz), −74.0 (m, *J*_FF_ ∼ 9 Hz, *J*_HF_ ∼ 0.5–1 Hz), and −76.5 (m, *J*_FF_ ∼ 9 Hz, *J*_HF_ ∼ 1–5 Hz). ^31^P{^1^H} NMR (121 MHz, C_6_D_6_): *δ* 129.4 (septet, *J* = 6 Hz). *In CDCl_3_, the overlapping CF_3_ signals in the ^13^C{^1^H} NMR spectrum are better resolved: *δ* 122.5 (q, *J* = 290 Hz), 122.3 (qd, *J* = 290 and 9 Hz), 122.0 (q, *J* = 290 Hz), 121.6 (qd, *J* = 290 and 2 Hz). **Reported coupling constants in the ^19^F NMR spectrum were determined by simulation with Mestrenova.

### Experimental NMR spectra, simulated ^19^F NMR spectra using MestreNova, and X-ray crystallography

See the ESI† for details.

## Conflicts of interest

There are no conflicts to declare.

## Supplementary Material

RA-011-D1RA05926B-s001

RA-011-D1RA05926B-s002
