# Supplementary material for: Trimerization and cyclization of reactive P-functionalities confined within OCO pincers
Source: RSC Adv. 2021 Aug 25;11(46):28602–13. doi: 10.1039/d1ra05926b (PMC9038090; doi:10.1039/d1ra05926b)
Supplement: RA-011-D1RA05926B-s001 [file RA-011-D1RA05926B-s001.pdf]

Electronic Supplementary Material (ESI) for RSC Advances.
This journal is © The Royal Society of Chemistry 2021

Supporting Information for

Trimerization and Cyclization of Reactive P-Functionalities Confined Within OCO Pincers

Beatrice L. Chinen,[†] Jakub Hyvl,[†] Daniel F. Brayton,[†] Matthew M. Riek,[†] Wesley Y. Yoshida,[†] Timothy W. Chapp,[‡] Arnold L. Rheingold,[£] and Matthew F. Cain[*,†]

[†] Department of Chemistry, University of Hawai‘i at Mānoa, 2545 McCarthy Mall, Honolulu, Hawaii 96822, United States

[‡] Department of Chemistry, Allegheny College, 520 N. Main Street, Meadville, PA, United States 16335

[£] Department of Chemistry, University of California, San Diego, 9500 Gilman Drive, La Jolla, California 92093, United States

1. Experimentals and NMR Spectra for New Compounds (page S1-S43)
2. Simulated ^{19}F NMR Spectra using MestreNova (S44-S49)
3. Experimental Details for Crystal Structure Refinement and Acquisition (S50 – S114)

1. Experimentals and NMR Spectra for New Compounds:

General Experimental Details.

Unless otherwise specified, all reactions were performed under an atmosphere of nitrogen in an MBraun or Vacuum Atmospheres glovebox or using standard Schlenk techniques. All glassware was dried overnight in an oven at 140 °C prior to use. Solvents used in the glove box were purchased directly from chemical suppliers (Aldrich or Acros), pumped directly into the glove box, and stored over oven-activated 4 or 5 Å molecular sieves (Aldrich). Solvents used outside the glove box were purged with N_2 for 30 min and stored over molecular sieves. $TMSCF_3$ was dried by cryogenic transfer. ^1H, $^{13}C\{^1H\}$, ^{19}F, and $^{31}P\{^1H\}$ NMR spectra were recorded on a Varian Mercury-300 (300/75/282/121 MHz), Varian Unity Inova-500 (500/126/470/202 MHz), or Agilent 600 DD2 (600/151/565/243 MHz) spectrometer at ambient temperature. Chemical shifts are reported in ppm downfield of tetramethylsilane using the solvent as internal standard (^1H $CDCl_3$ = 7.27 ppm, ^1H C_6D_6 = 7.16 ppm, ^{13}C $CDCl_3$ = 77.16 ppm, ^{13}C C_6D_6 = 128.06 ppm). Multiplicities are abbreviated as br (broad), s (singlet), d (doublet), t (triplet), q (quartet), or m (multiplet). Coupling constants (*J*) are reported in Hertz (Hz). Flash column chromatography was performed on silica gel (40-63 µm, SiliCycle). High-resolution mass spectrometry (HRMS) was recorded on an Agilent 6545 LC-MS Q-ToF spectrometer (NSF–1532310). Dimethyl 2-bromoisophthalate and $[Me_4N][F]$ were synthesized according to the published procedures listed below.[1,2] All other chemicals were used as received, unless otherwise noted.

[1] Courchay, F.C.; Sworen, J.C.; Ghiviriga, I.; Abboud, K.A.; Wagener, K.B. Understanding Structural Isomerization during Ruthenium-Catalyzed Olefin Metathesis: A Deuterium Labeling Study. *Organometallics* **2006**, *25*, 6074-6086.

[2] Kolomeitsev, A.A.; Seifert, F.U.; Roeschenthaler, G.-V. Simple Preparation of Difluorophosphoranes Using Anhydrous Zinc and Tetramethylammonium Fluorides. *J. Fluorine Chem.* **1995**, *71 (1)*, 47-49.

Synthesis of 1.

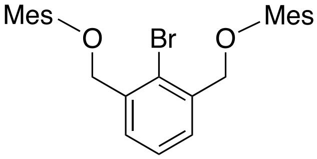

1

In the glovebox, 2,4,6-trimethylphenol (3.00 g, 22.03 mmol, 2.5 equiv.) was dissolved in THF (100 mL), and a 1.6 M solution of *n*-BuLi in hexanes (13.0 mL, 20.8 mmol, 2.3 equiv.) was added dropwise at room temperature. The reaction mixture was stirred for 15 min. Subsequently, tribromide **D** (3.086 g, 9.0 mmol) was added to the stirred solution, and the reaction mixture was transferred to a Schlenk bomb equipped with a Teflon screw cap and heated at 100 °C for 1 d. The volatiles were then removed under reduced pressure, and the crude residue was extracted with a toluene:hexane mixture (1:1, 3 x 15 mL). The combined extracts were filtered through a Celite plug, and the filtrate was concentrated under vacuum then purified by column chromatography (silica gel, toluene:hexane 1:1, R_f = 0.63). The title product (2.64 g, 5.822 mmol) was obtained as white plates in 65% yield. X-Ray quality crystals were obtained by slow evaporation from a concentrated solution of **1** in *n*-hexanes.

Anal. Calcd for $C_{26}H_{29}BrO_2$: C, 68.87; H, 6.45. Found: C, 68.77; H, 6.27. ^{1}H NMR (500 MHz, $CDCl_3$): δ 7.78 (d, J = 7.6 Hz, 2H, Ar), 7.51 (t, J = 7.6 Hz, 1H, Ar), 6.89 (s, 4H, Ar), 4.93 (s, 4H, CH_2), 2.30 (s, 12H, Me), 2.29 (s, 6H, Me). $^{13}C\{^{1}H\}$ NMR (126 MHz, $CDCl_3$): δ 153.5 (Ar), 137.8 (Ar), 133.6 (Ar), 130.9 (Ar), 129.6 (Ar), 127.9 (Ar), 127.7 (Ar), 121.6 (Ar), 73.3 (CH_2), 20.9 (CH_3), 16.4 (CH_3).

^{1}H NMR Spectrum of **1**

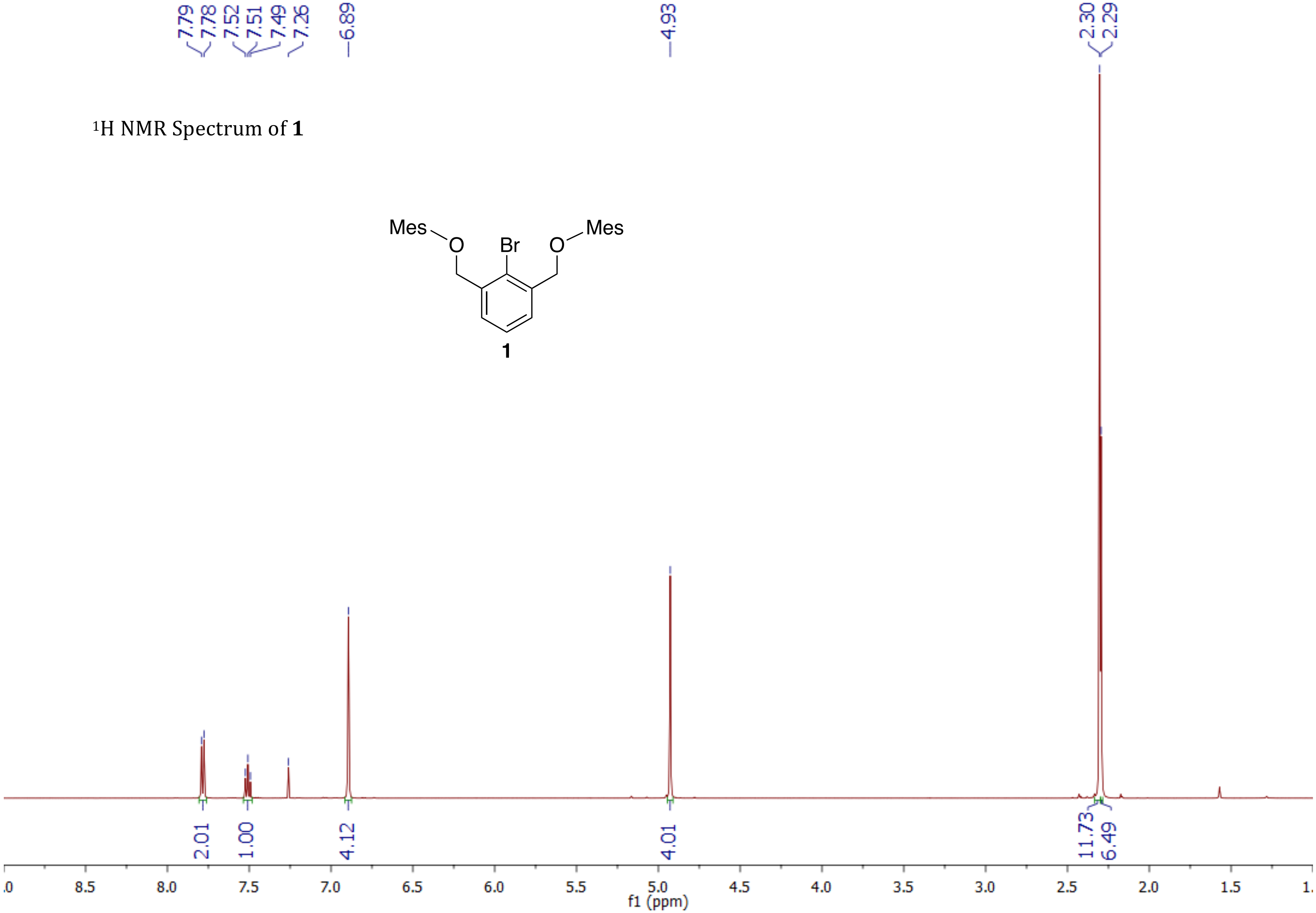

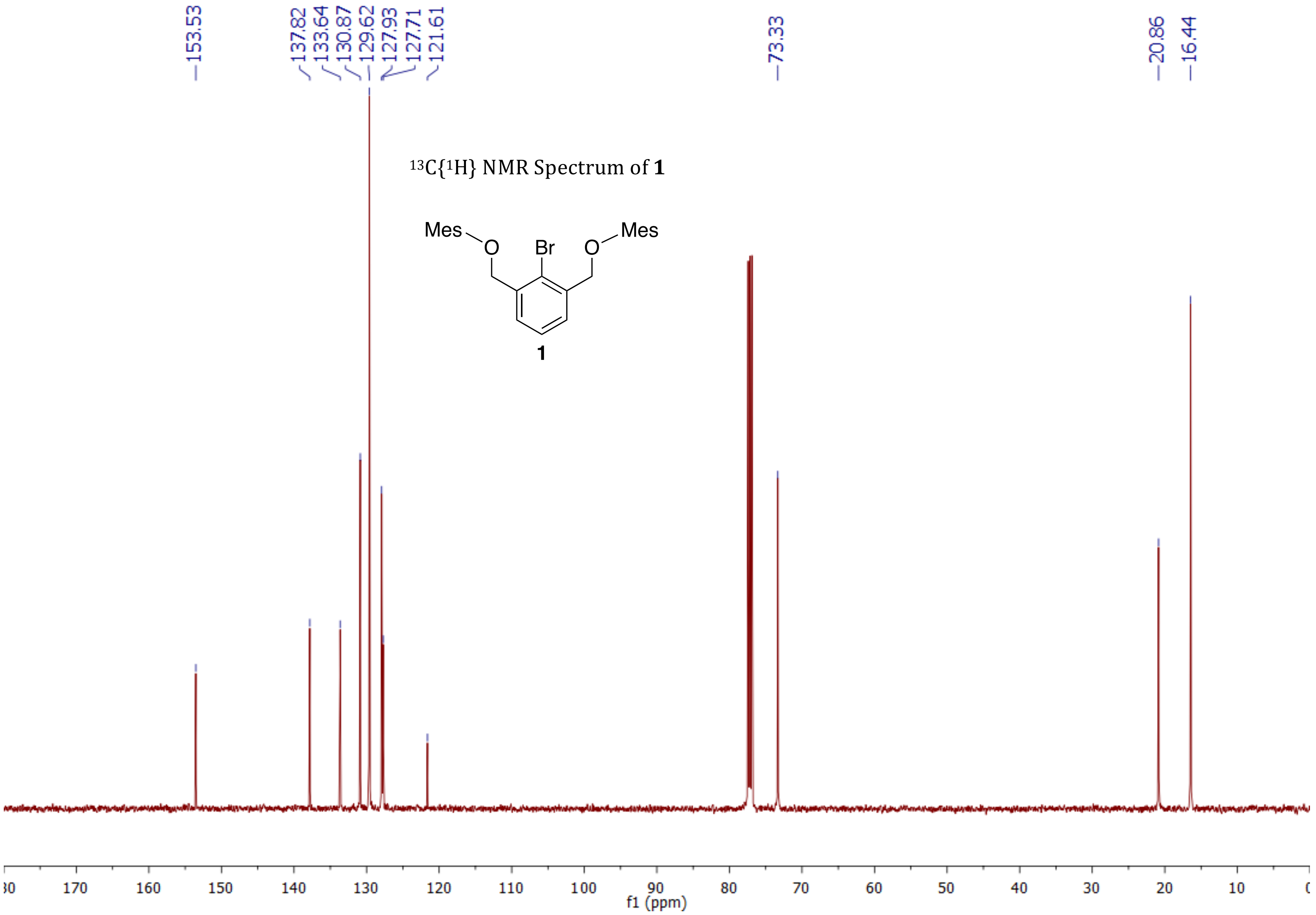
13C{1H} NMR Spectrum of 1
Mes
O
Br
O
Mes
1
153.53
137.82
133.64
130.87
129.62
127.93
127.71
121.61
73.33
20.86
16.44
f1 (ppm)

DEPT of **1**

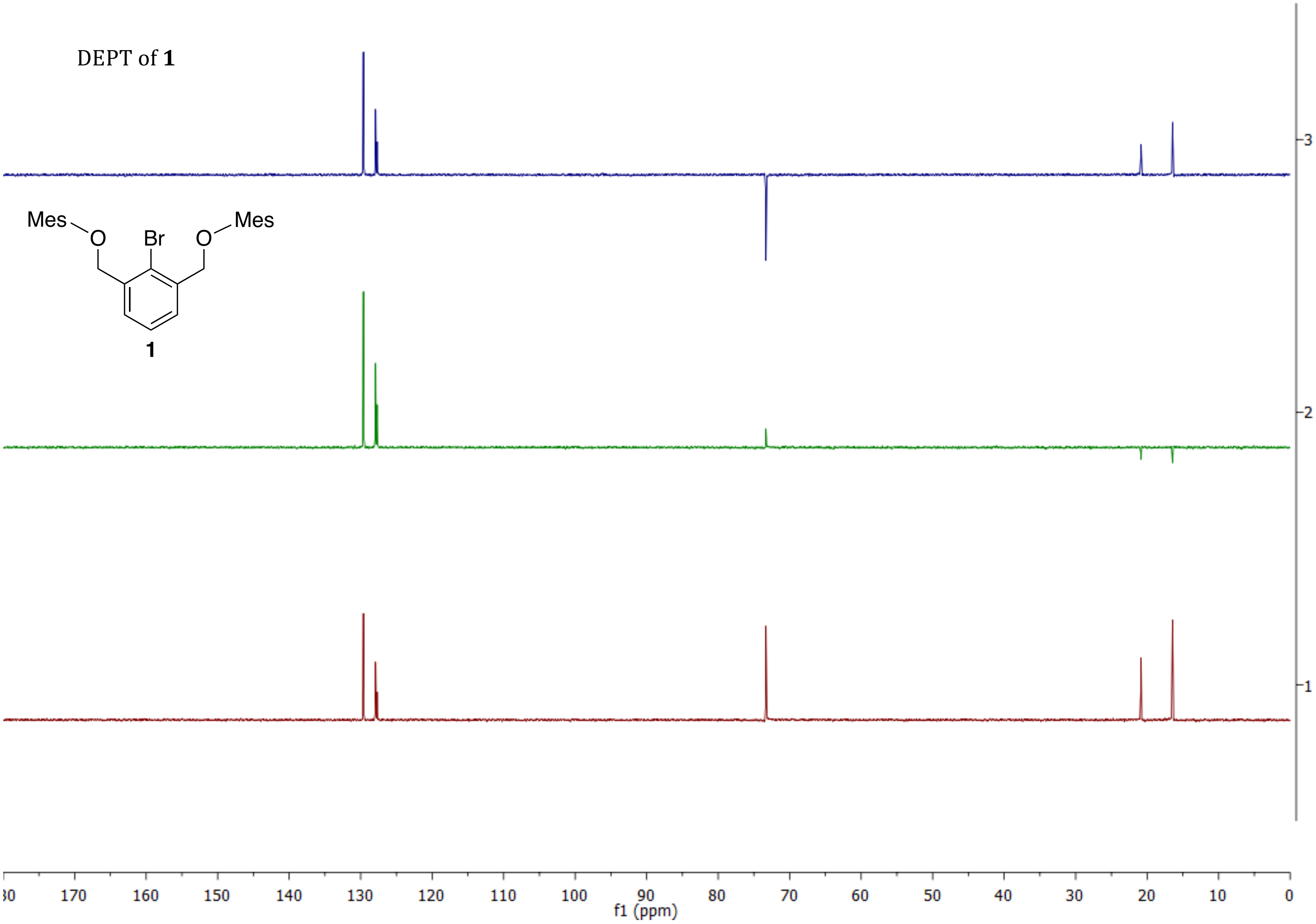

Mes
O
Br
O
Mes
1
3
2
1
30
170
160
150
140
130
120
110
100
90
f1 (ppm)
80
70
60
50
40
30
20
10
0

CENTC Elemental Analysis Facility
University of Rochester
Rochester, NY 14627 USA
Email: ealab@chem.rochester.edu

**Date of report**	6/2/2017 8:22:15PM
**User ID**	Administrator
**Comments**	JH154 [Cain / Hyvl]

DATE & TIME	6/2/2017 1:56:40 PM	P_ID	EA LAB
SAMPLE ID	17305D	USER ID	Administrator
WEIGHT (mg)	2.082	MODE	CHN

CARBON	68.768%
HYDROGEN	6.267%
NITROGEN	0.0%

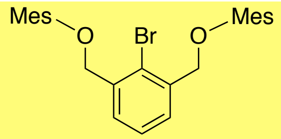

1

Chemical Formula: $C_{26}H_{29}BrO_2$
Exact Mass: 452.1351
Molecular Weight: 453.4200
Elemental Analysis: C, 68.87; H, 6.45; Br, 17.62; [illegible]

DATE & TIME	6/2/2017 1:50:38 PM
SAMPLE ID	17305
WEIGHT (mg)	2.012

CARBON	68.781%
HYDROGEN	6.166%
NITROGEN	0.092%

Acknowledgment

Analytical data were obtained from the CENTC Elemental Analysis Facility at the University of Rochester, funded by NSF CHE-0650456.

Instrumentation

Microanalysis samples were weighed with a PerkinElmer Model AD-6 Autobalance and their compositions were determined with a PerkinElmer 2400 Series II Analyzer.

Synthesis of 2, Its "Half" Trifluoromethylated Derivative, and Diol E:

Synthesis of "Half" Trifluoromethylated.

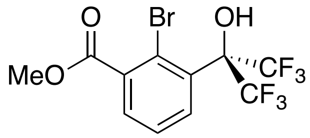

Initial attempts to prepare diol **E** (via its recently reported literature prep[3]) using six equiv of Ruppert's reagent ($TMSCF_3$) and four equiv of $[Me_4N][F]$ yielded a mixture of trifluoromethylated products; the "half" trifluoromethylated derivative could be isolated cleanly in low yield.

In the glovebox, dimethyl 2-bromoisophthalate (4.40 g, 16.1 mmol) and $[Me_4N][F]$ (6.00 g, 64.4 mmol, 4 equiv.) were suspended in DME (65 mL) in a Schlenk bomb with a stir bar. The bomb was taken out of the glove box, placed on the Schlenk line, and cooled to -40 °C in a dry ice/acetonitrile bath. $TMSCF_3$ (9.52 mL, 64.4 mmol, 4 equiv.) was injected *via* syringe through a rubber septum, and the mixture was stirred at -40 °C for 4 h. A second aliquot of $TMSCF_3$ (4.76 mL, 32.2 mmol, 2 equiv.) was injected in a similar fashion, and the mixture was stirred at -40 °C for another 6 h. After warming to room temperature, NH_4Cl (*aq*) was added at 0 °C, and the mixture was extracted with toluene (3 x 50 mL). The combined organic layers were washed with water then brine, dried over $MgSO_4$, filtered, and the filtrate was concentrated under vacuum. The crude product mixture was purified by column chromatography with DCM/hexanes (1:2) as the eluent. The pure fractions were recrystallized from dichloromethane (8 mL) layered with hexanes (50 mL) at 4 °C. The colorless precipitate was filtered and rinsed with cold pentane (40 mL), affording crystals suitable for X-ray diffraction (0.24 g, 0.49 mmol, 3%).

Anal. Calcd for $C_{11}H_7BrF_6O_3$: C, 34.67; H, 1.85. Found: C, 34.76; H, 1.57. ^1H NMR (500 MHz, $CDCl_3$): δ 7.81 (br d, *J* = 15 Hz, 1H, Ar), 7.60 (dd, *J* = 5, 15 Hz, 1H, Ar), 7.50 (t, *J* = 15 Hz, 1H, Ar), 5.34 (br s, 1H, OH), 3.99 (s, 3H, Me). ^{13}C NMR (126 MHz, $CDCl_3$): δ 138.9 (Ar), 132.1 (Ar), 130.9 (Ar), 128.7 (Ar), 127.6 (Ar), 122.4 (q, *J* = 290 Hz, CF_3), 118.0 (Ar), 53.1 (Me), the quaternary carbon and C=O were not observed. ^{19}F NMR (470 MHz, $CDCl_3$): δ -73.2 (s).

[3] Y. Imada, T. Kukita, H. Nakano and Y. Yamamoto, *Bull. Chem. Soc. Jpn.*, 2016, **89**, 546-548.

^{1}H NMR (300 MHz, $CDCl_3$) spectrum

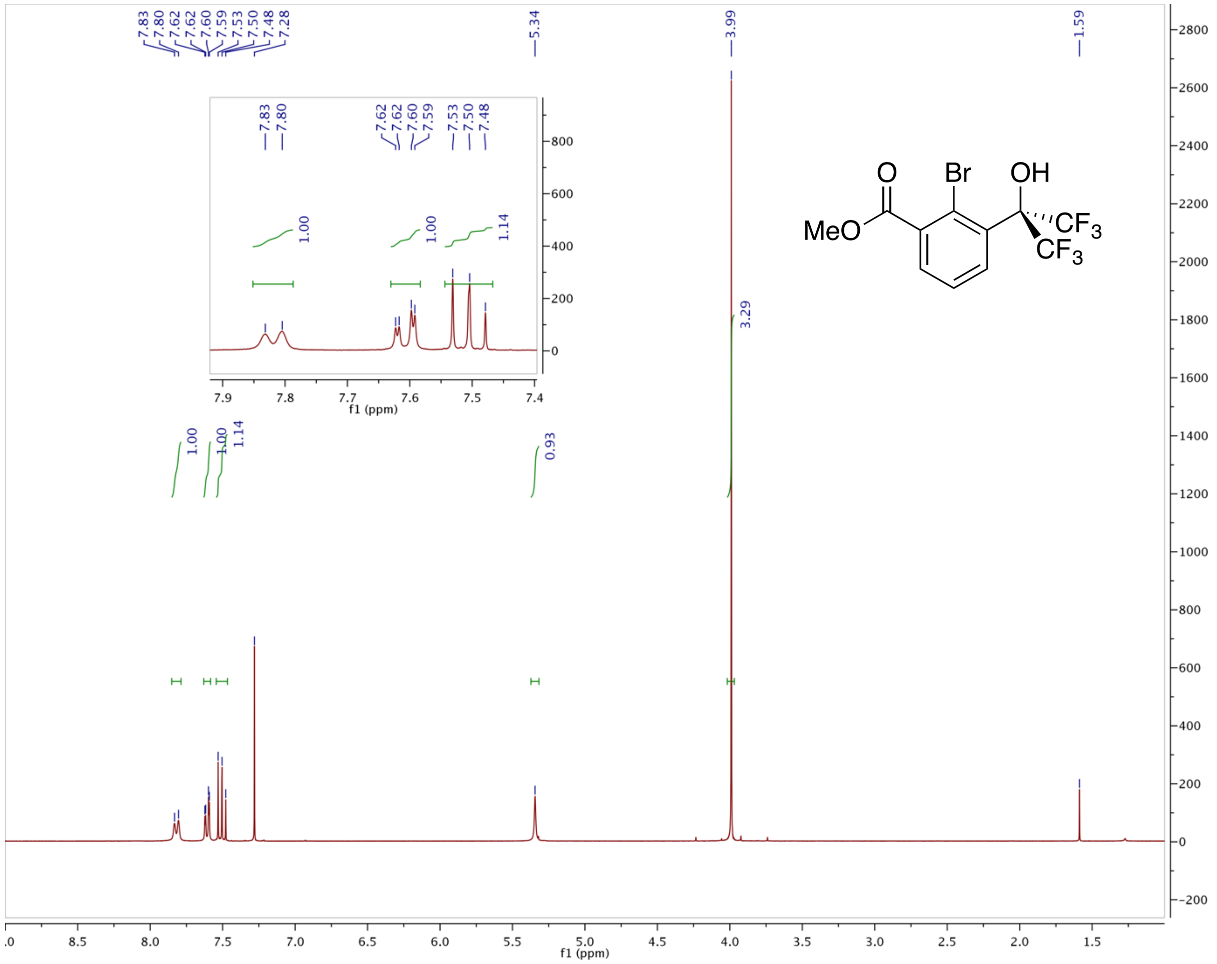

^{13}C NMR (126 MHz, $CDCl_3$) spectrum

(inset displays CF_3 quartet)

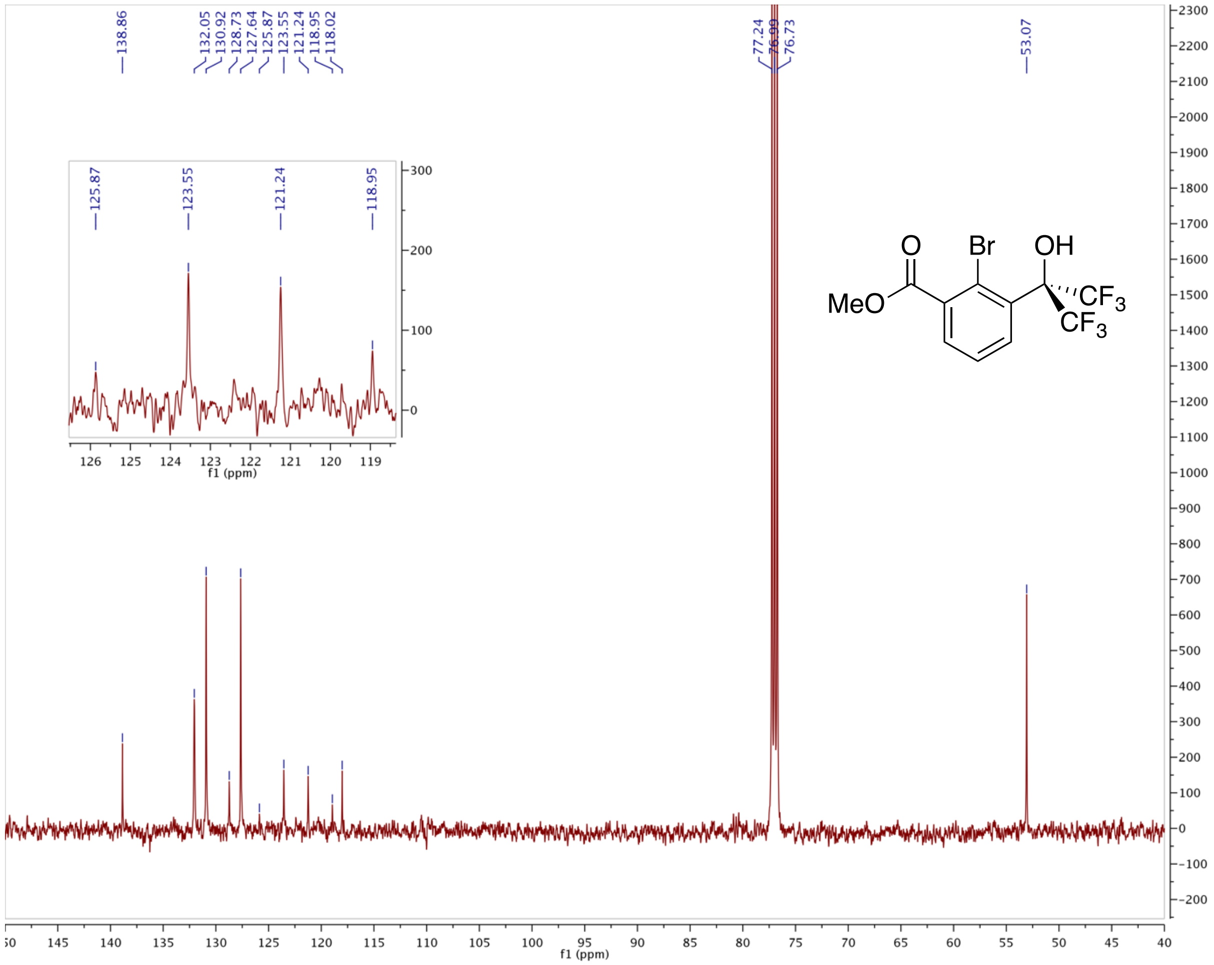

^{19}F NMR (282 MHz, $CDCl_3$) spectrum

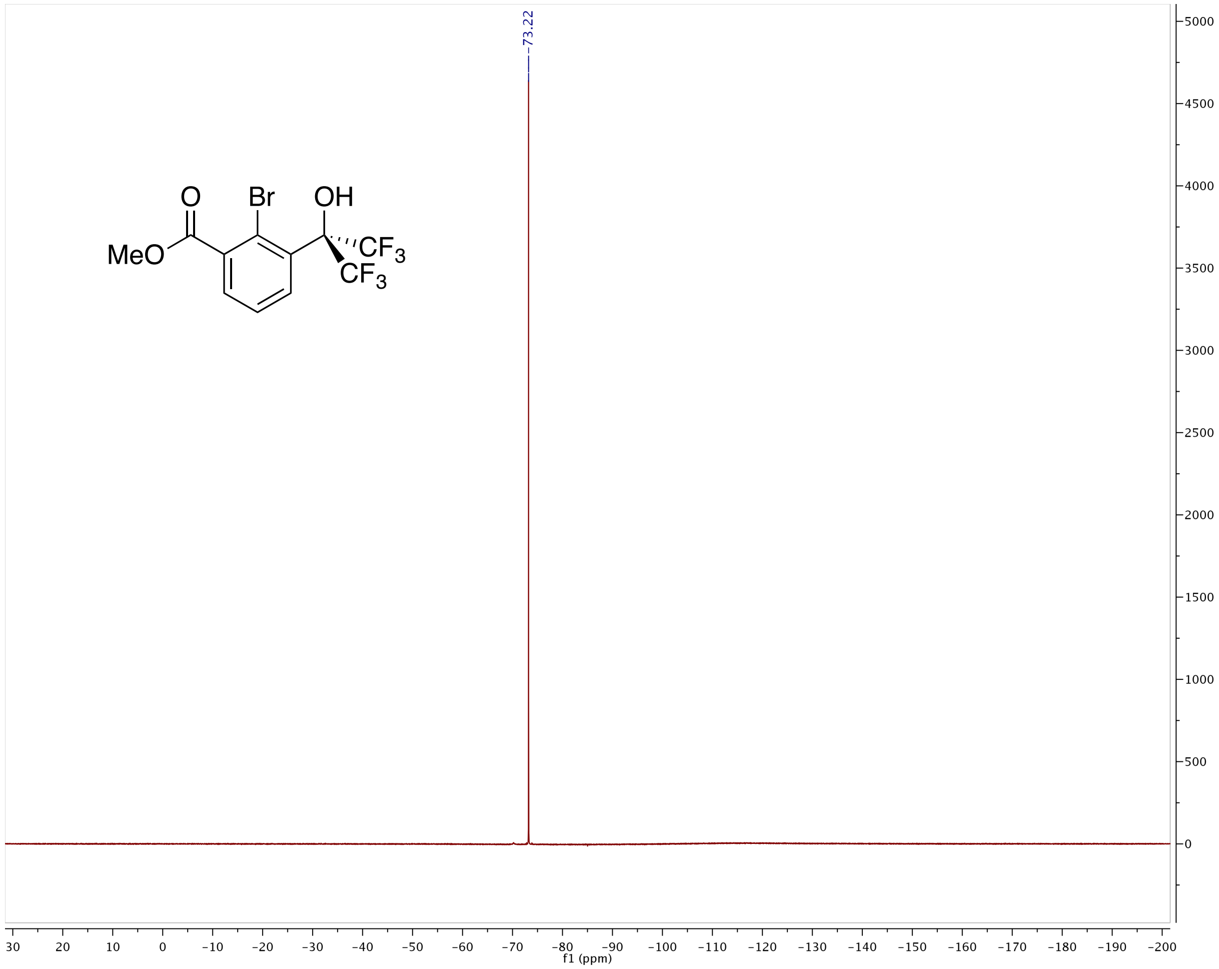

CENTC Elemental Analysis Facility
University of Rochester
Rochester, NY 14627 USA
Email: ealab@chem.rochester.edu

**Date of report**	5/31/2019 4:51:42PM
**User ID**	Administrator

**Comments**	B23 [Cain / Chinen]

DATE & TIME	5/31/2019 2:28:33 PM	P_ID	EA LAB
SAMPLE ID	19329	USER ID	Administrator
WEIGHT (mg)	1.971	MODE	CHN

CARBON	34.759%
HYDROGEN	1.571%
NITROGEN	0.024%

Acknowledgment

Analytical data were obtained from the CENTC Elemental Analysis Facility at the University of Rochester, funded by NSF CHE-0650456.

Instrumentation

Microanalysis samples were weighed with a PerkinElmer Model AD6000 Autobalance and their compositions were determined with a PerkinElmer 2400 Series II Analyzer.

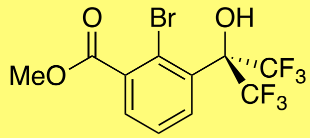

Chemical Formula: $C_{11}H_7BrF_6O_3$
Exact Mass: 379.9483
Molecular Weight: 381.0684
Elemental Analysis: C, 34.67; H, 1.85; Br, 20.97; F, 29.91; O, 12.60

Synthesis of Diol E.

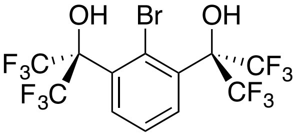

E

In order to access diol **E**, the published prep[4] had to be modified. Specifically, a large excess of $TMSCF_3$ was required, and the solvent was changed from DME to DMF.

In the glovebox, dimethyl 2-bromoisophthalate (3.20 g, 11.7 mmol) and $[Me_4N][F]$ (21.83 g, 234.4 mmol, 20 equiv.) were dissolved in DMF (300 mL) in a Schlenk flask with a stir bar. The flask was taken out of the glove box, placed on the Schlenk line, and fitted with an air-free addition funnel under positive N_2 pressure. The flask was cooled to -40 °C in a dry ice/acetonitrile bath with vigorous stirring. $TMSCF_3$ (34.6 mL, 234 mmol, 20 equiv.) was transferred to the addition funnel *via* cannula and added dropwise to the Schlenk flask over several min. The mixture was stirred at -40 °C for 4 h then another dose of $TMSCF_3$ (17.3 mL, 117 mmol, 10 equiv.) was added in the same manner. The mixture was stirred overnight at -40 °C, warmed to RT, and stirred for an additional 1 h. NH_4Cl (*aq*) was added at 0 °C, and the reaction mixture was extracted with toluene (3 x 50 mL). The combined organic layers were washed with water, brine, dried over $MgSO_4$, filtered, and the filtrate was concentrated under vacuum. The resulting yellow oil was purified by column chromatography using Et_2O/*n*-hexane (1:2) as the eluent to give pale yellow solid **7** (4.67 g, 9.55 mmol, 82%). The ^{1}H,$^{13}C\{^{1}H\}$, and ^{19}F NMR spectra were consistent with the published report,[4] but a slight impurity at 3.99 ppm in the ^{1}H NMR spectrum persisted.

[4] Y. Imada, T. Kukita, H. Nakano and Y. Yamamoto, *Bull. Chem. Soc. Jpn.*, 2016, **89**, 546-548.

Synthesis of 2.

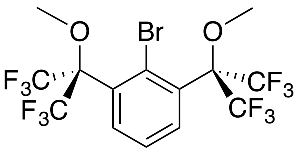

2

Diol **E** (3.61 g, 7.38 mmol) and DMF (180 mL) were combined in a Schlenk flask with a stir bar. The solution was purged with N_2 on the Schlenk line for 30 min, then K_2CO_3 (5.10 g, 36.9 mmol, 5 equiv.) was added with stirring under positive N_2 pressure. Next, MeI (2.30 mL, 36.9 mmol, 5 equiv.) was injected *via* syringe, and the reaction mixture was stirred overnight, then quenched with NH_4Cl (*aq*) and extracted with toluene (3 x 50 mL). The combined organic layers were washed with water, brine, dried over $MgSO_4$, filtered, and the filtrate concentrated under vacuum, resulting in pale yellow solid **2** (2.97 g, 5.74 mmol, 78%). Recrystallization from acetonitrile (8 mL) at 4 °C afforded crystals suitable for X-ray diffraction.

Anal. Calcd for $C_{14}H_9BrO_2F_{12}$: C, 32.52; H, 1.75. Found: C, 32.71; H, 1.77. ^1H NMR (500 MHz, $CDCl_3$): δ 7.76 (d, *J* = 10 Hz, 2H, Ar), 7.51 (t, *J* = 10 Hz, 1H, Ar), 3.52 (s, 6H, Me). $^{13}C\{^1H\}$ NMR (151 MHz, $CDCl_3$): δ 134.1 (Ar), 129.6 (Ar), 127.0 (Ar), 123.5 (Ar), 122.4 (q, *J* = 290 Hz, CF_3), 86.5 (septet, *J* = 29 Hz, $C(CF_3)_2$), 54.8 (OMe). ^{19}F NMR (471 MHz, $CDCl_3$): δ -66.9 (s).

^{1}H NMR (500 MHz, $CDCl_3$) spectrum

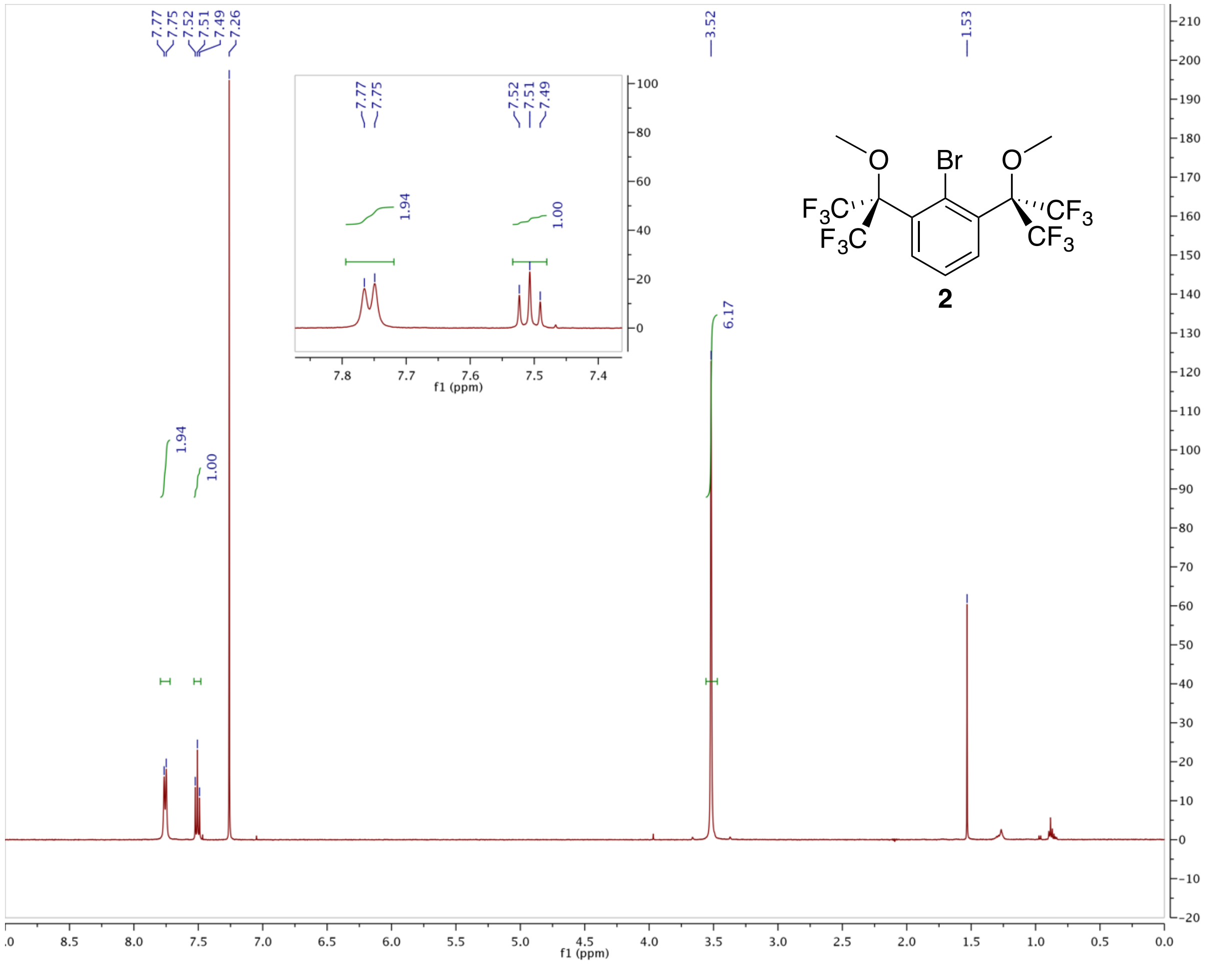

$^{13}C\{^1H\}$ NMR (151 MHz, $CDCl_3$) spectrum
(insets display CF_3 quartet and quaternary carbon atoms)

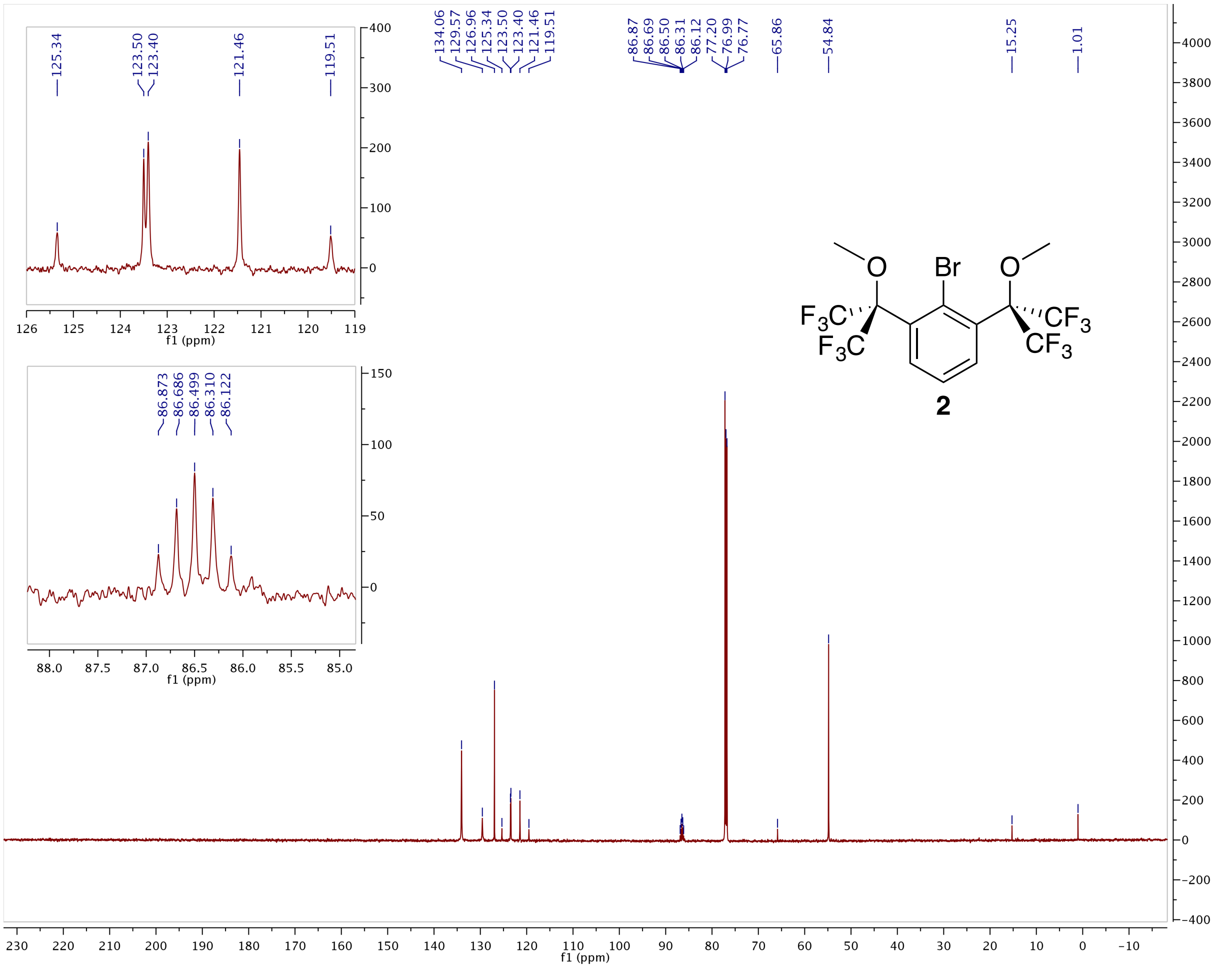

Trace ether and grease are present.

^{19}F NMR (471 MHz, $CDCl_3$) spectrum

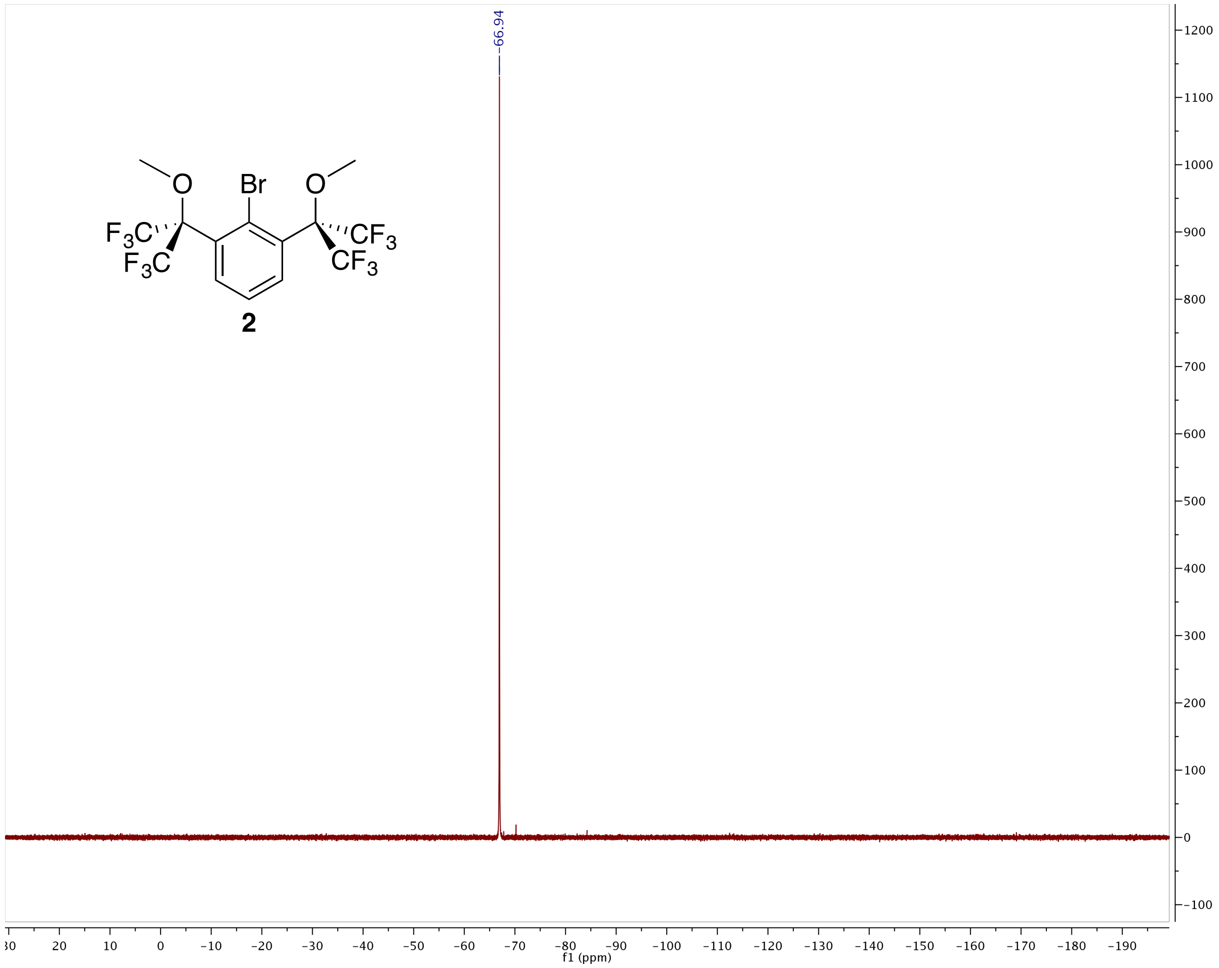

CENTC Elemental Analysis Facility
University of Rochester
Rochester, NY 14627 USA
Email: ealab@chem.rochester.edu

**Date of report**	7/2/2021 4:00:04PM
**User ID**	Administrator
**Comments**	B89 [Cain / Chinen]

DATE & TIME	7/2/2021 10:01:04 AM	P_ID	EA LAB
SAMPLE ID	21372	USER ID	Administrator
WEIGHT (mg)	2.431	MODE	CHN

CARBON	32.708%
HYDROGEN	1.769%
NITROGEN	0.007%

Acknowledgment

Analytical data were obtained from the CENTC Elemental Analysis Facility at the University of Rochester, funded by NSF CHE-0650456.

Instrumentation

Microanalysis samples were weighed with a PerkinElmer Model AD6000 Autobalance and their compositions were determined with a PerkinElmer 2400 Series II Analyzer.

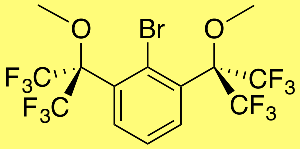

Chemical Formula: $C_{14}H_9BrF_{12}O_2$
Exact Mass: 515.9594
Molecular Weight: 517.1088
m/z: 515.9594 (100.0%), 517.9574 (97.3%), 516.9628 (15.1%), 518.9607 (14.7%), 517.9661 (1.1%), 519.9641 (1.0%)
Elemental Analysis: C, 32.52; H, 1.75; Br, 15.45; F, 44.09; O, 6.19

Synthesis of 3.

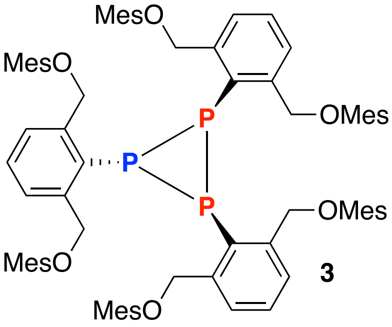

3

OCO-supported aryl bromide **1** (1.134 g, 2.50 mmol) was loaded into a Schlenk flask, dissolved in Et_2O (60 mL), taken outside of the glovebox, and cooled to -78 °C. A 1.6 M solution of *n*-BuLi in hexanes (1.7 mL, 2.72 mmol, 1.1 equiv) was injected (under N_2), and the reaction mixture was stirred at -78 °C for 5 min. A second Schlenk flask containing a solution of PCl_3 (550 mg, 4.01 mmol, 1.6 equiv.) in Et_2O (5 mL) was transferred via cannula to the cooled reaction mixture. The reaction mixture was stirred at -78 °C for 10 min and then at room temperature (RT) for 1 h, leading to the precipitation of a white solid. The volatiles were removed under reduced pressure, the Schlenk flask was brought back into the glovebox, the residue was triturated with Et_2O (10 mL), and the volatiles were again removed. The residue was dissolved in THF (20 mL), and PMe_3 was added (485 mg, 6.375 mmol, 2.6 equiv.). The reaction mixture was stirred at RT for 1 d. The organic volatiles were removed under reduced pressure, and the residue was extracted with toluene (3 x 20 mL). The combined extracts were filtered through a Celite plug, the filtrate was concentrated to dryness, triturated with *n*-pentane (10 mL), and again concentrated to dryness under reduced pressure. The resulting residue was triturated with acetonitrile (10 mL) and stirred at RT for 1 h, generating a white precipitate that was collected by filtration and dried (565 mg, 0.466 mmol, 56%). X-Ray quality crystals of **3** were obtained by recrystallization from a solution of hot acetonitrile.

Anal. Calcd for $C_{78}H_{87}O_6P_3$: C, 77.20; H, 7.23. Found: C, 76.89; H, 7.14. $^{31}P\{^1H\}$ NMR (202 MHz, $CDCl_3$): δ -116.2 (d, $^1J_{PP}$ = 186 Hz), -144.0 (t, $^1J_{PP}$ = 186 Hz). ^1H NMR (500 MHz, $CDCl_3$): δ 7.71 (d, J = 7.7 Hz, 2H, Ar^1), 7.40 (t, J = 7.7 Hz, 1H, Ar^1), 7.33-7.28 (m, 4H, Ar^2), 7.28-7.22 (m, 2H, Ar^2), 6.71 (s, 8H, Ar^2), 6.62 (s, 4H, Ar^1), 5.39 (s, 4H, CH_2-Ar^1), 4.71 (br, 8H, CH_2-Ar^2), 2.25 (s, 12H, Ar^1), 2.20 (s, 6H, Ar^1), 2.02 (s, 12H, Ar^2), 1.95 (br, 24H, Ar^2). ^{13}C NMR (126 MHz, $CDCl_3$): δ 153.46 (Ar), 153.44 (Ar), 143.5 (Ar), 143.4 (d, J_{PC} = 6.9 Hz, Ar), 133.6 (Ar), 133.0 (Ar), 132.9 (Ar), 130.7 (Ar), 130.5 (Ar), 130.4 (Ar), 129.6 (Ar), 129.4 (Ar), 129.0 (Ar), 128.4 (d, J_{PC} = 10.0 Hz, Ar), 127.9 (Ar), 126.4 (Ar), 73.2 (CH_2), 72.8 (d, J_{PC} = 7.6 Hz, CH_2), 20.9 (CH_3), 16.7 (CH_3), 16.4 (CH_3), 16.3 (CH_3).

Ar^1 refers to the aryl groups associated with the Blue P

Ar^2 refers to the aryl groups associated with the Red Ps.

^{1}H NMR Spectrum of **3**

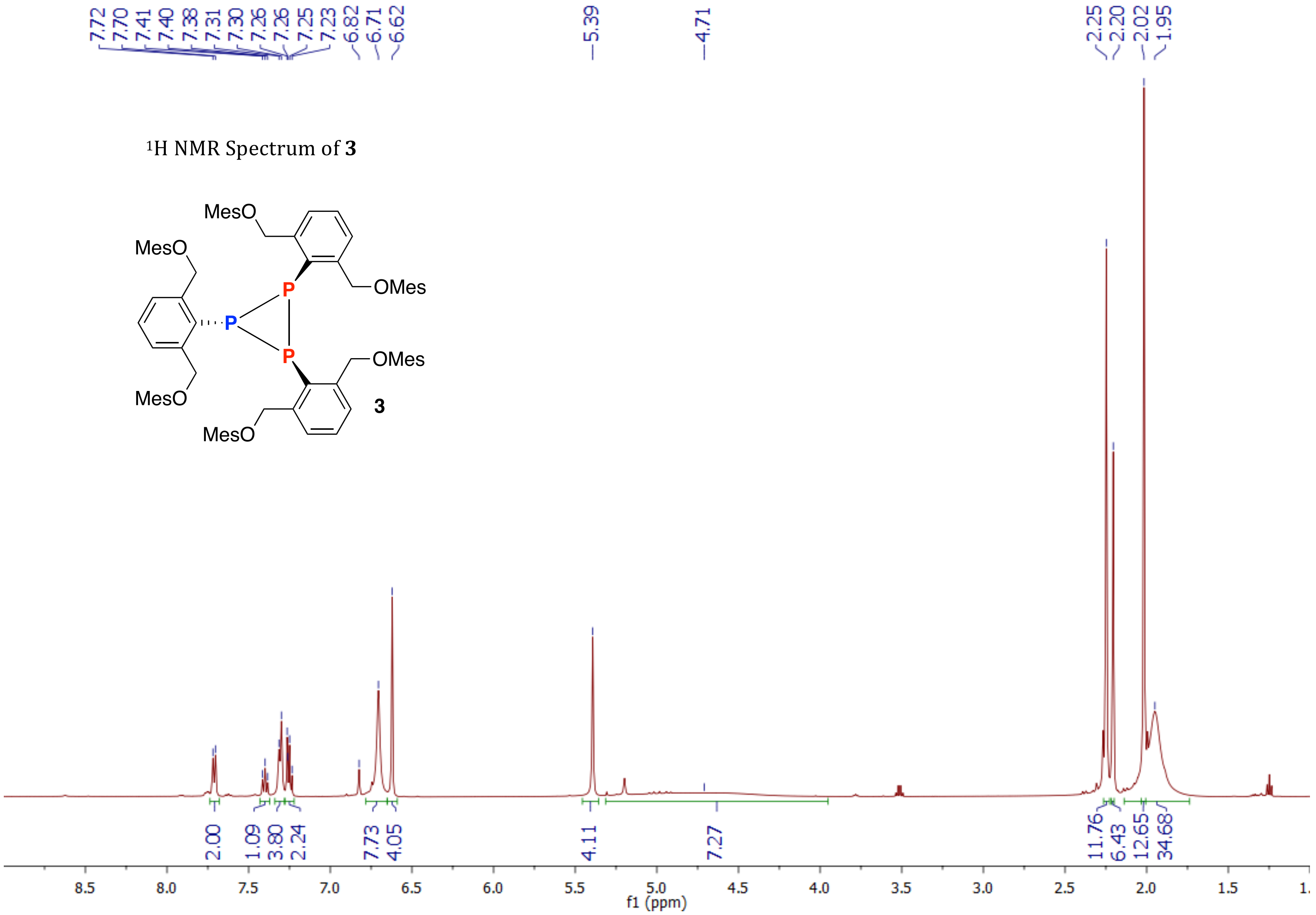

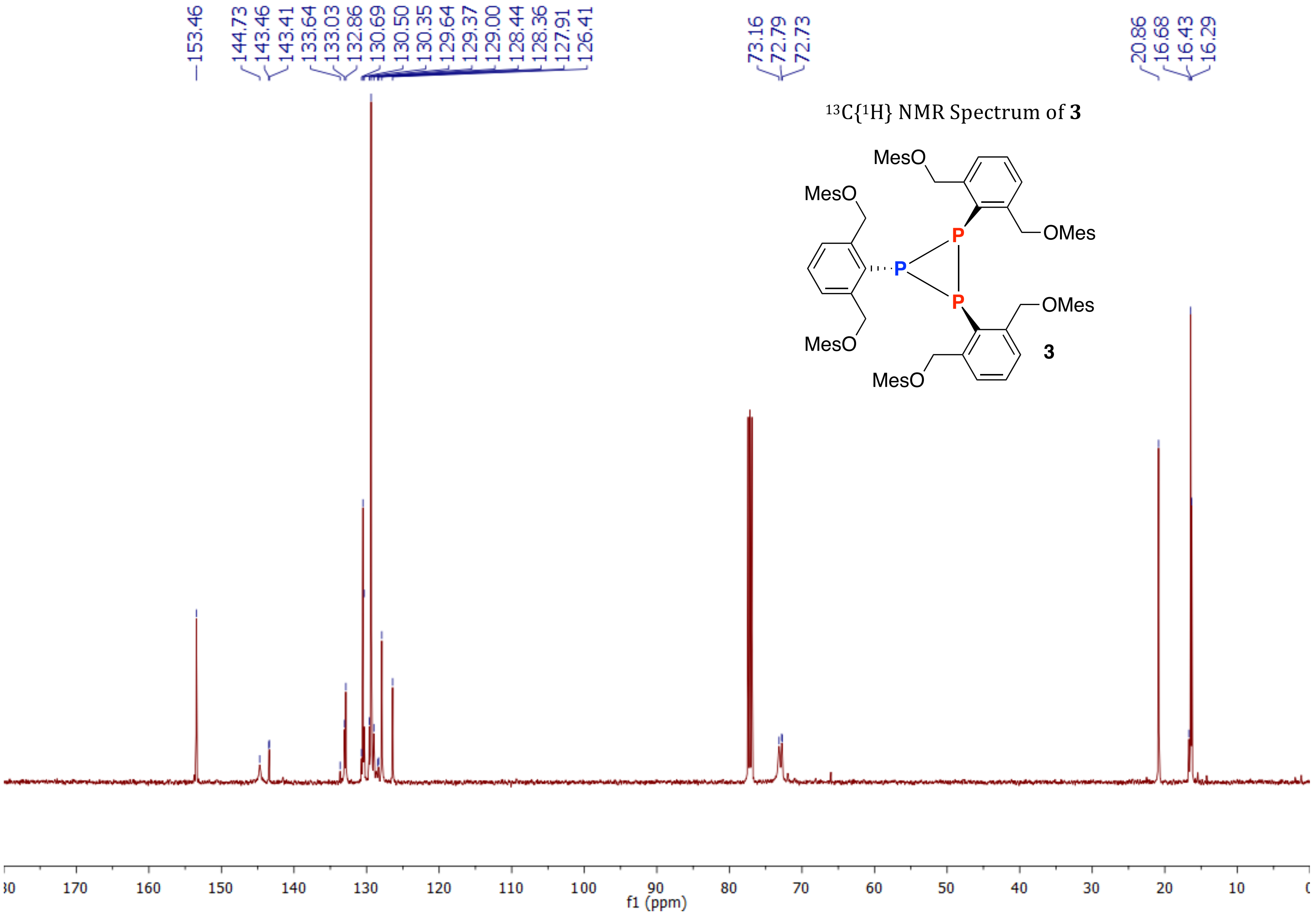
153.46
144.73
143.46
143.41
133.64
133.03
132.86
130.69
130.50
130.35
129.64
129.37
129.00
128.44
128.36
127.91
126.41
73.16
72.79
72.73
20.86
16.68
16.43
16.29
$^{13}C\{^1H\}$ NMR Spectrum of **3**
MesO
MesO
OMes
P
P
P
OMes
MesO
MesO
3
170
160
150
140
130
120
110
100
90
80
70
60
50
40
30
20
10
f1 (ppm)

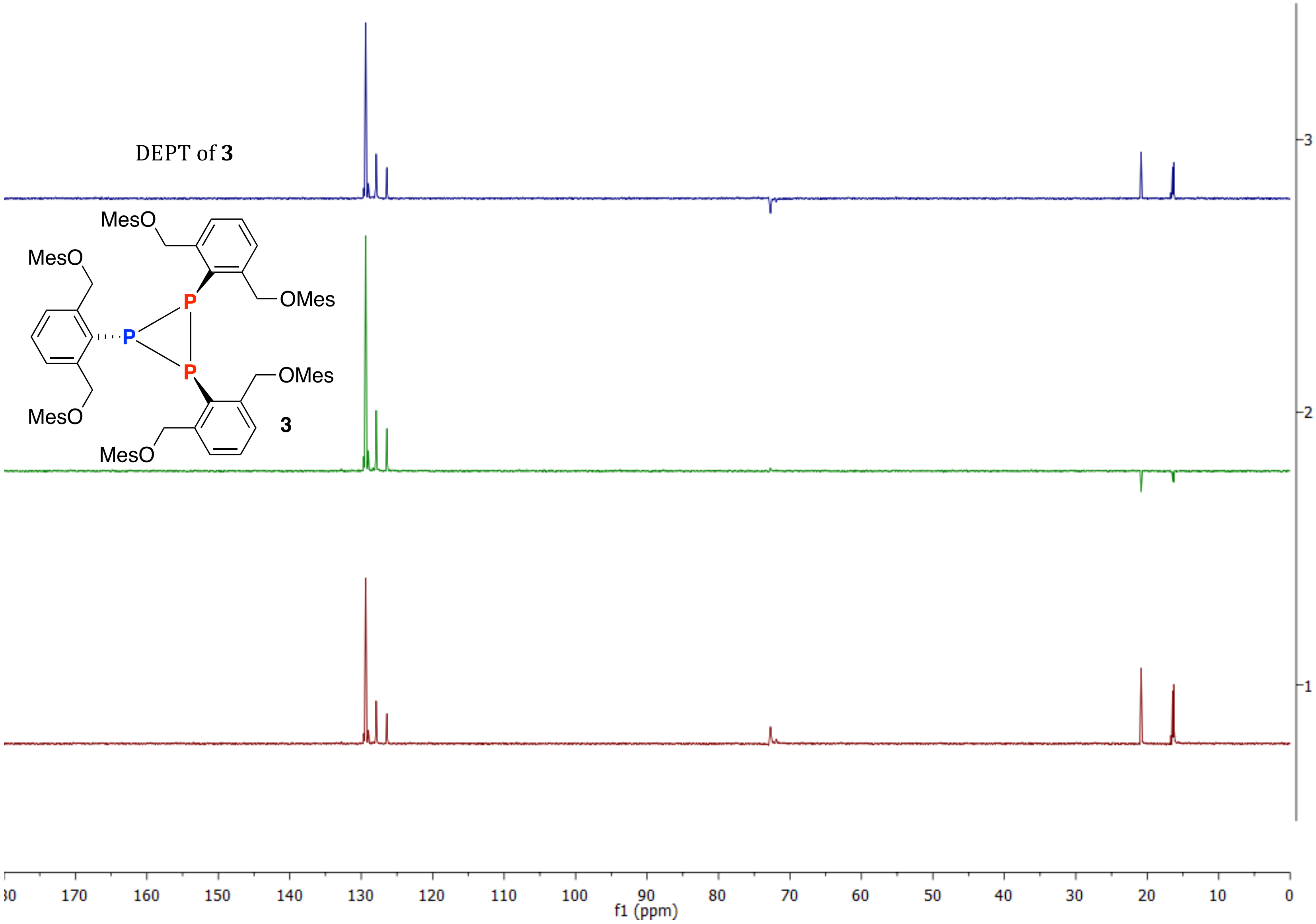
DEPT of 3
MesO
MesO
P
P
P
OMes
OMes
MesO
MesO
3
3
2
1
170
160
150
140
130
120
110
100
90
f1 (ppm)
80
70
60
50
40
30
20
10
0

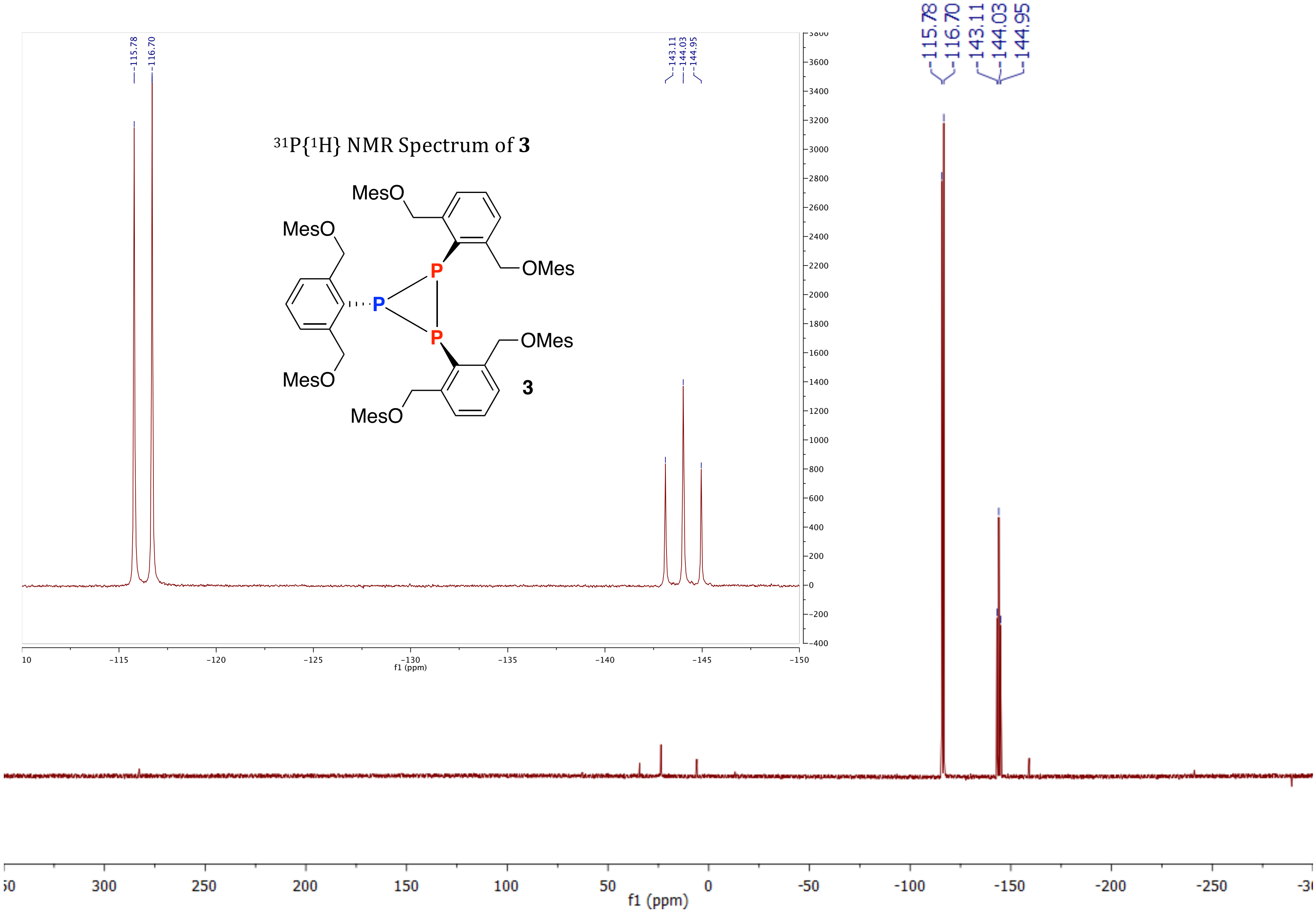
$^{31}P\{^{1}H\}$ NMR Spectrum of **3**
MesO
OMes
P
3
-115.78
-116.70
-143.11
-144.03
-144.95
f1 (ppm)

CENTC Elemental Analysis Facility
University of Rochester
Rochester, NY 14627 USA
Email: ealab@chem.rochester.edu

**Date of report**	6/2/2017 9:04:46PM
**User ID**	Administrator
**Comments**	JH167 [Cain / Hyvl]

DATE & TIME	6/2/2017 9:04:04 PM
SAMPLE ID	17322D
WEIGHT (mg)	2.720

CARBON	76.728%
HYDROGEN	7.258%
NITROGEN	0.131%

DATE & TIME	6/2/2017 8:52:18 PM
SAMPLE ID	17322
WEIGHT (mg)	2.145

CARBON	76.893%
HYDROGEN	7.138%
NITROGEN	-.095%

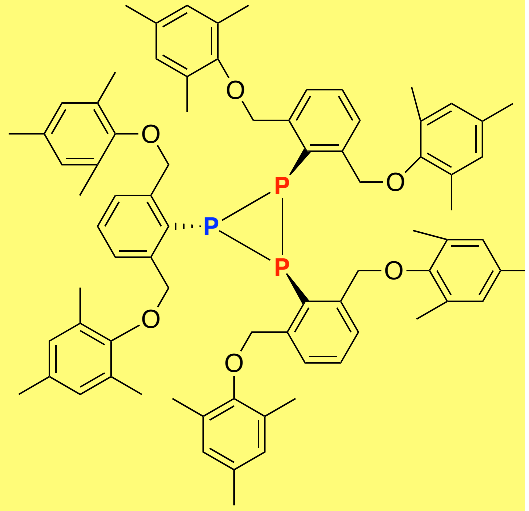

Chemical Formula: $C_{78}H_{87}O_6P_3$
Exact Mass: 1212.5716
Molecular Weight: 1213.4693
Elemental Analysis: C, 77.20; H, 7.23; O, 7.91; P, [illegible]

Special Handling

The sample was transferred under argon and was combusted in a tin capsule that was crimp-sealed wi[illegible] a die apparatus.

Acknowledgment

Analytical data were obtained from the CENTC Elemental Analysis Facility at the University of Rocheste[illegible] funded by NSF CHE-0650456.

Instrumentation

Microanalysis samples were weighed with a PerkinElmer Model AD-6 Autobalance and their compositions were determined with a PerkinElmer 2400 Series II Analyzer. Air-sensitive samples we[illegible] handled in a VAC Atmospheres glovebox.

Generation and Observation of 5 by $^{31}P\{^1H\}$ and ^{19}F NMR Spectroscopy.

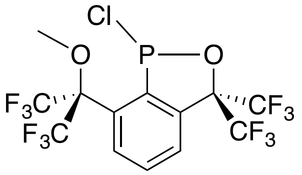

5

Fluorinated aryl bromide **2** (500 mg, 0.967 mmol) was dissolved in 6 mL of THF in a vial with a stir bar inside the glovebox and (*i*-Pr)MgCl·LiCl was added dropwise via syringe (0.82 mL, 1.064 mmol, 1.1 equiv, 1.3 M in THF), resulting in a homogeneous, yellow reaction mixture. The reaction mixture was stirred for 1 h at RT then directly filtered into 2 mL of a pre-cooled solution (-35 °C, 1 h) of PCl_3 (146 mg, 1.064 mmol, 1.1 equiv) in THF. The solution was warmed to RT for 1 h then analyzed by $^{31}P\{^1H\}$ NMR spectroscopy, revealing the presence of unreacted PCl_3 (218 ppm), *i*-$PrPCl_2$ (202 ppm) and the chlorophosphine (171 ppm). The "intermediate" product mixture was then concentrated under vacuum to remove the volatile and unwanted phosphorus byproducts (PCl_3 and *i*-$PrPCl_2$), leaving a pale yellow solid (405 mg, 0.751 mmol, 78%) that was reanalyzed by $^{31}P\{^1H\}$ and ^{19}F NMR spectroscopy in C_6D_6.

^{19}F NMR (282 MHz, C_6D_6):* δ -68.9 (m, J_{FF} ~ 8 Hz, J_{PF} ~ 2 Hz, J_{HF} ~0.5-1.5 Hz), -70.4 (m, J_{FF} ~ 8 Hz, J_{PF} ~ 4 Hz, J_{HF} ~ 0.2-2.6 Hz), 70.9 (s, impurity**), -73.4 (m, J_{FF} ~ 9 Hz, J_{PF} ~ 2 Hz, J_{HH} ~ 1 Hz), -76.3 (m, J_{FF} ~ 9 Hz, J_{PF} ~ 2 Hz, J_{HH} ~0.5-2 Hz). $^{31}P\{^1H\}$ NMR (121 MHz, C_6D_6): δ 166.4 (s).

*Reported coupling constants in the ^{19}F NMR spectrum were determined by simulation with Mestrenova.

**The impurity at 70.9 ppm in the ^{19}F NMR spectrum is the product of protodemetalation.

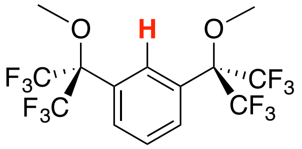

^{19}F NMR (282 MHz, C_6D_6) spectra

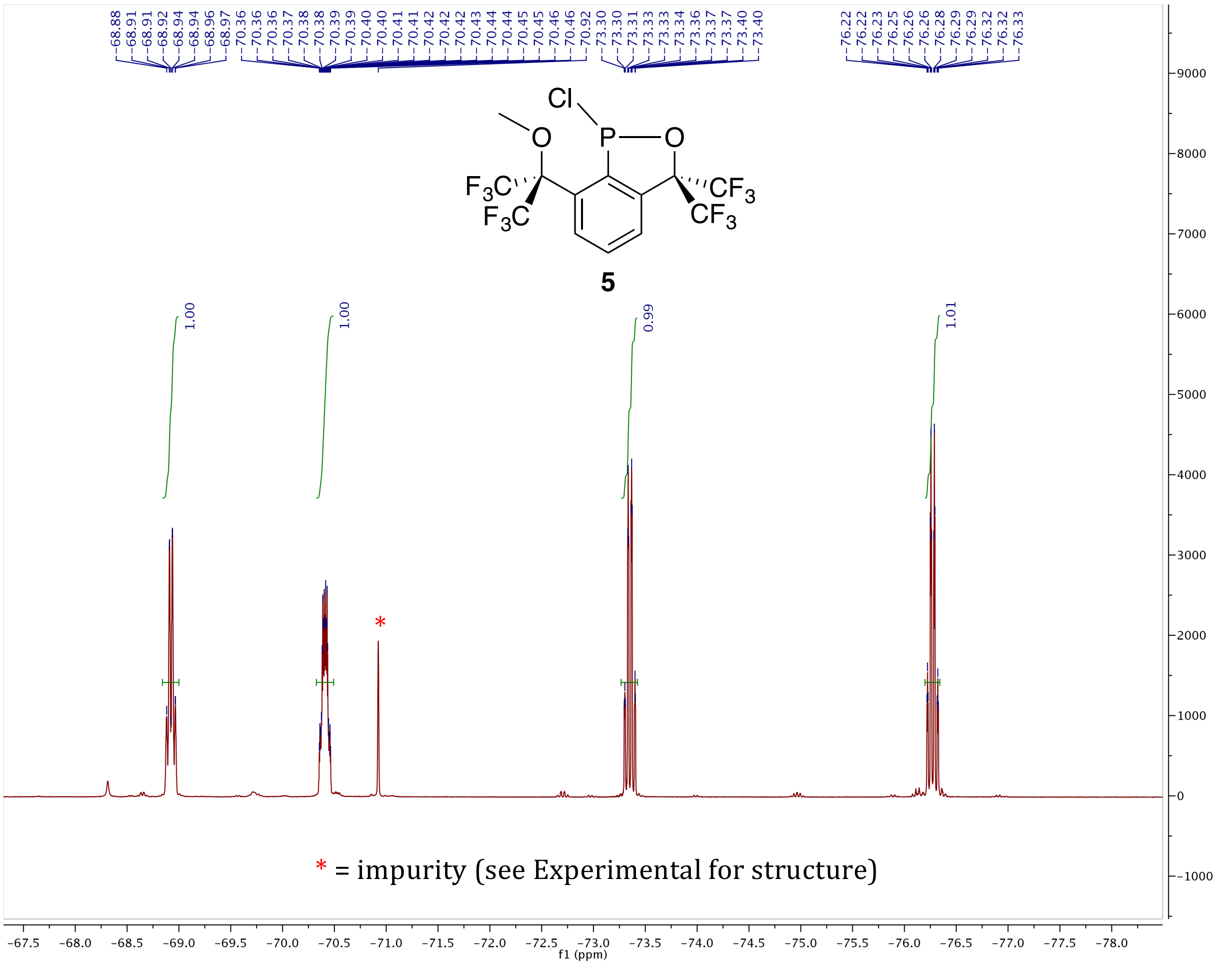

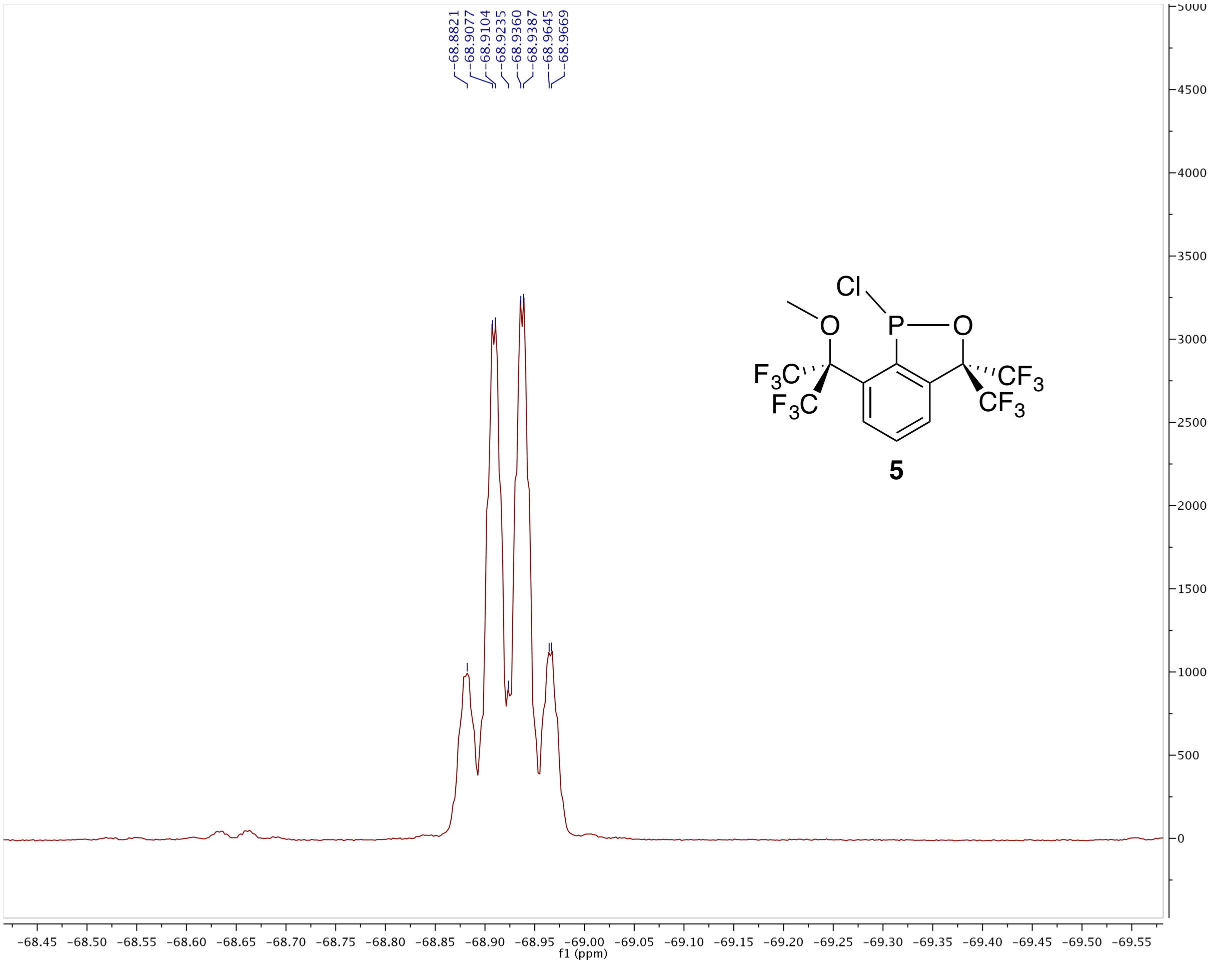
-68.8821
-68.9077
-68.9104
-68.9235
-68.9360
-68.9387
-68.9645
-68.9669
5
f1 (ppm)

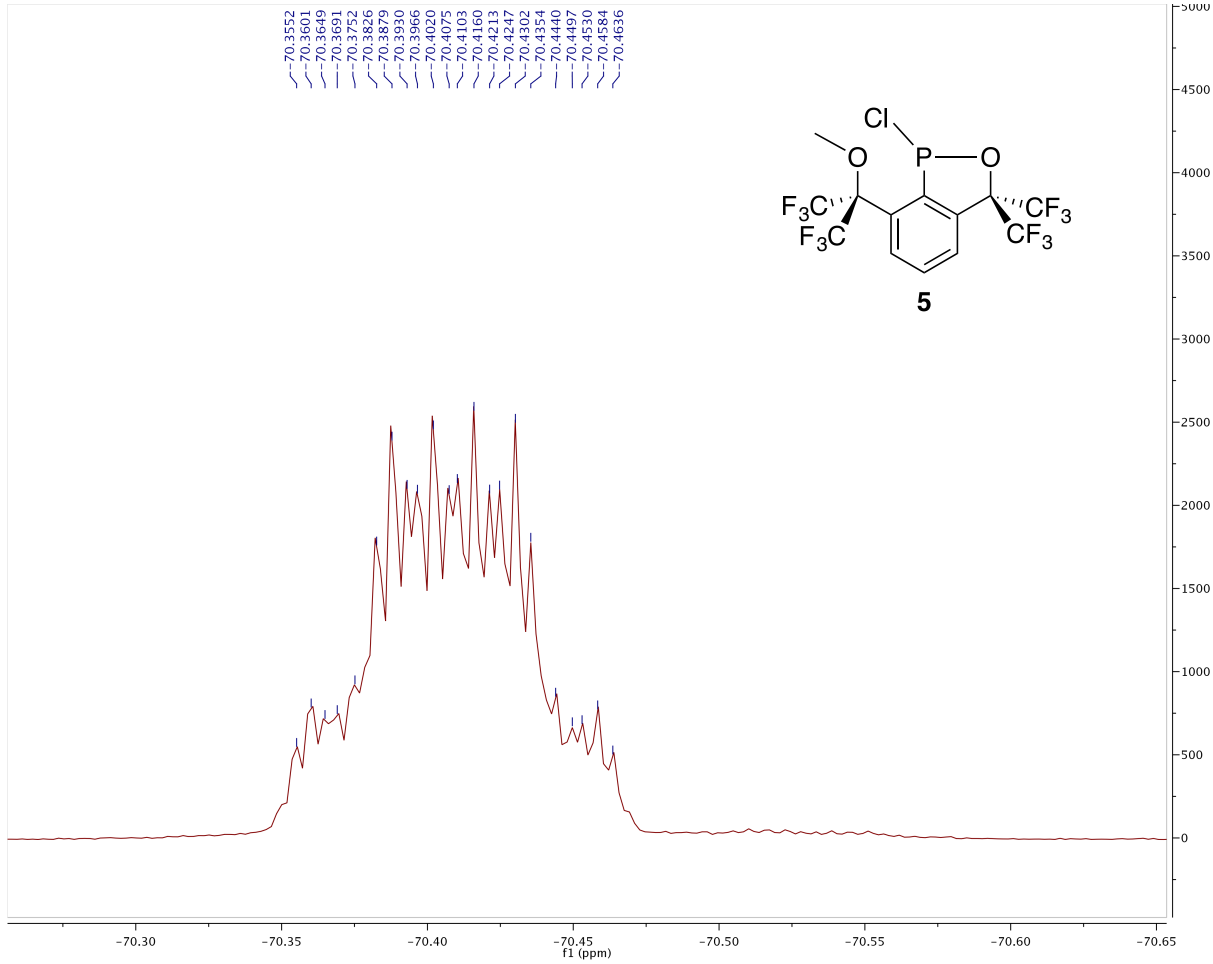

-70.3552
-70.3601
-70.3649
-70.3691
-70.3752
-70.3826
-70.3879
-70.3930
-70.3966
-70.4020
-70.4075
-70.4103
-70.4160
-70.4213
-70.4247
-70.4302
-70.4354
-70.4440
-70.4497
-70.4530
-70.4584
-70.4636
Cl
P
O
O
F3C
F3C
CF3
CF3
5
5000
4500
4000
3500
3000
2500
2000
1500
1000
500
0
-70.30
-70.35
-70.40
-70.45
-70.50
-70.55
-70.60
-70.65
f1 (ppm)

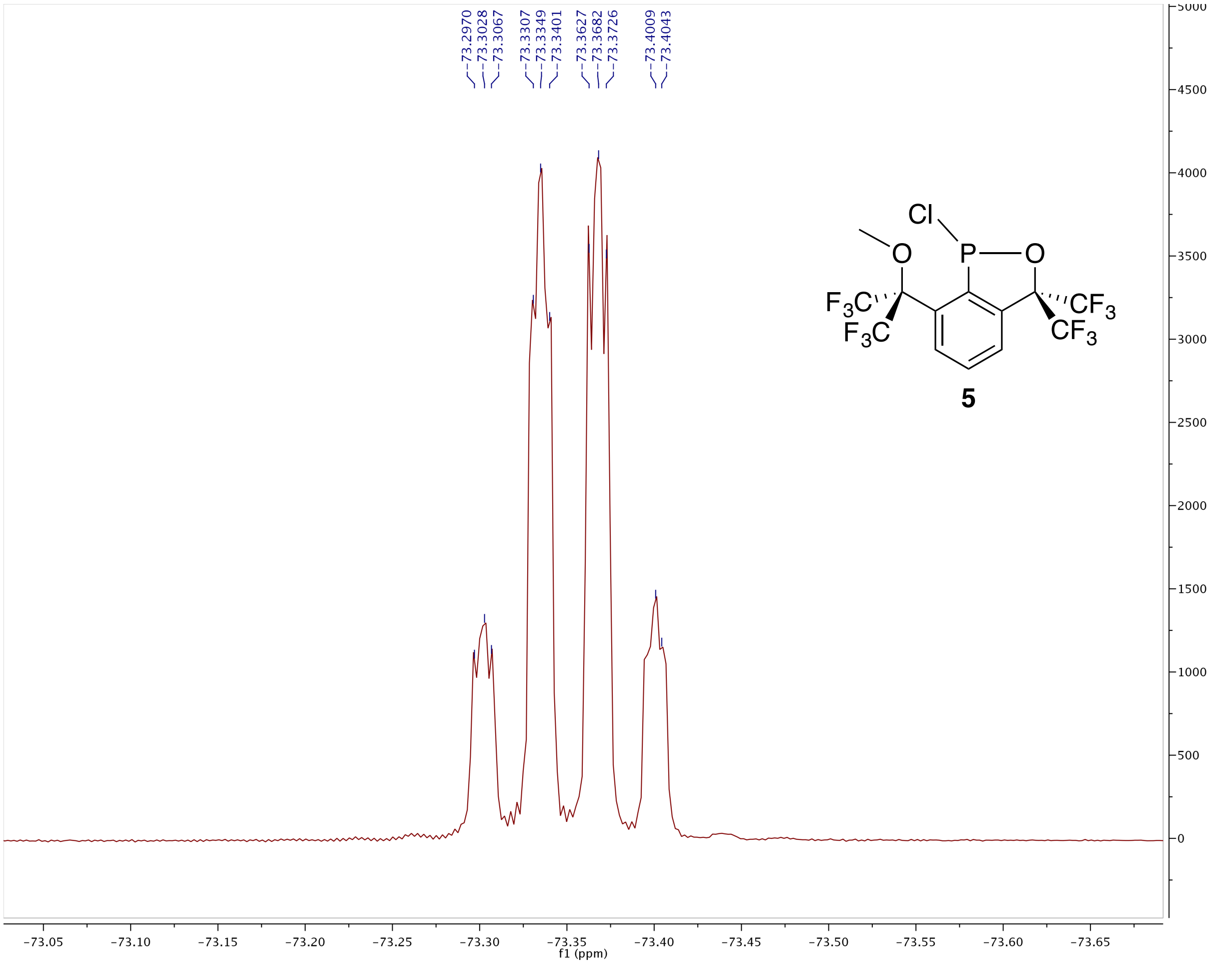
-73.2970
-73.3028
-73.3067
-73.3307
-73.3349
-73.3401
-73.3627
-73.3682
-73.3726
-73.4009
-73.4043
Cl
P
O
O
F3C
F3C
CF3
CF3
5
f1 (ppm)

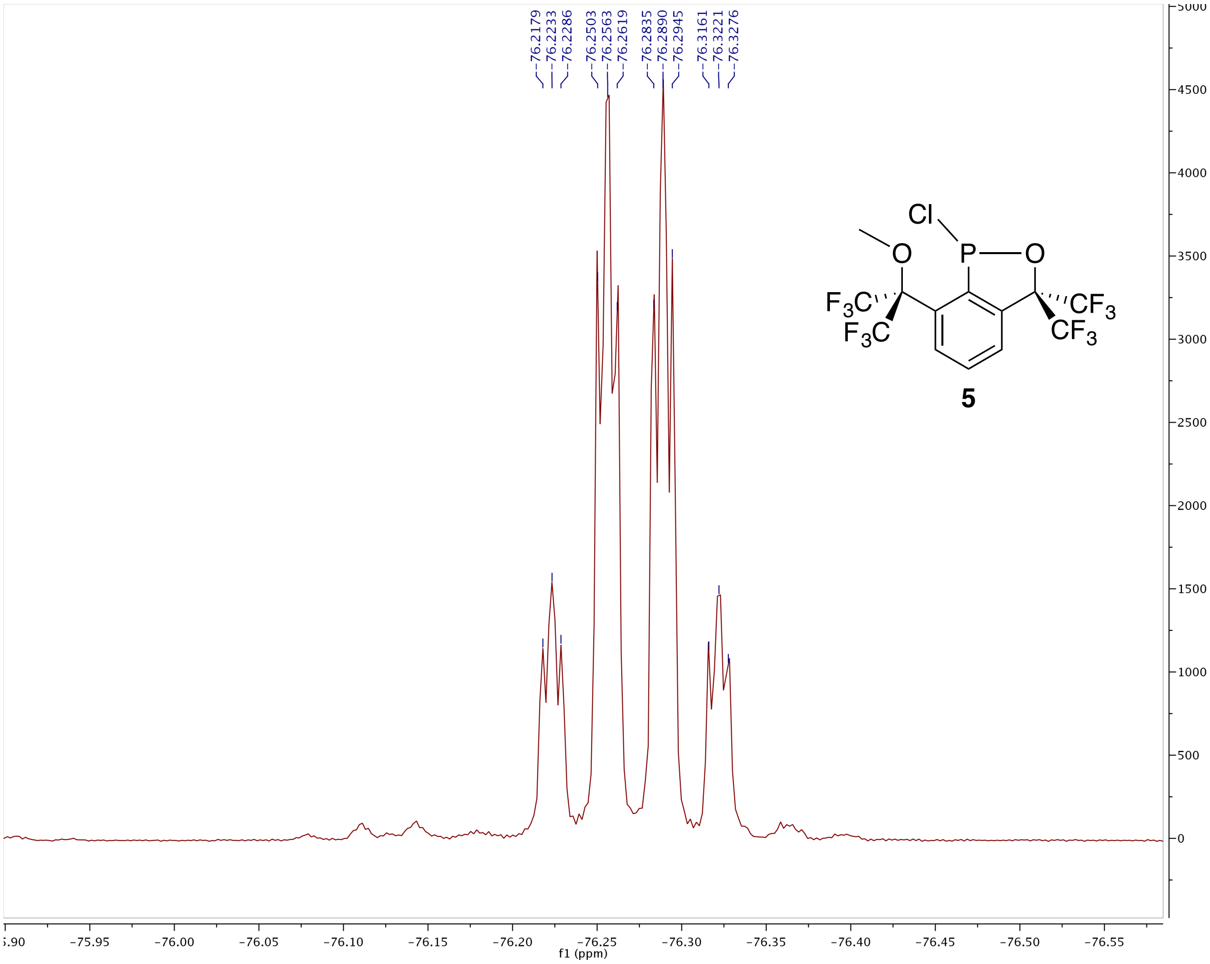

-76.2179
-76.2233
-76.2286
-76.2503
-76.2563
-76.2619
-76.2835
-76.2890
-76.2945
-76.3161
-76.3221
-76.3276
Cl
P
O
O
F3C
F3C
CF3
CF3
5
f1 (ppm)

$^{31}P\{^1H\}$ NMR (121 MHz, C_6D_6) spectrum

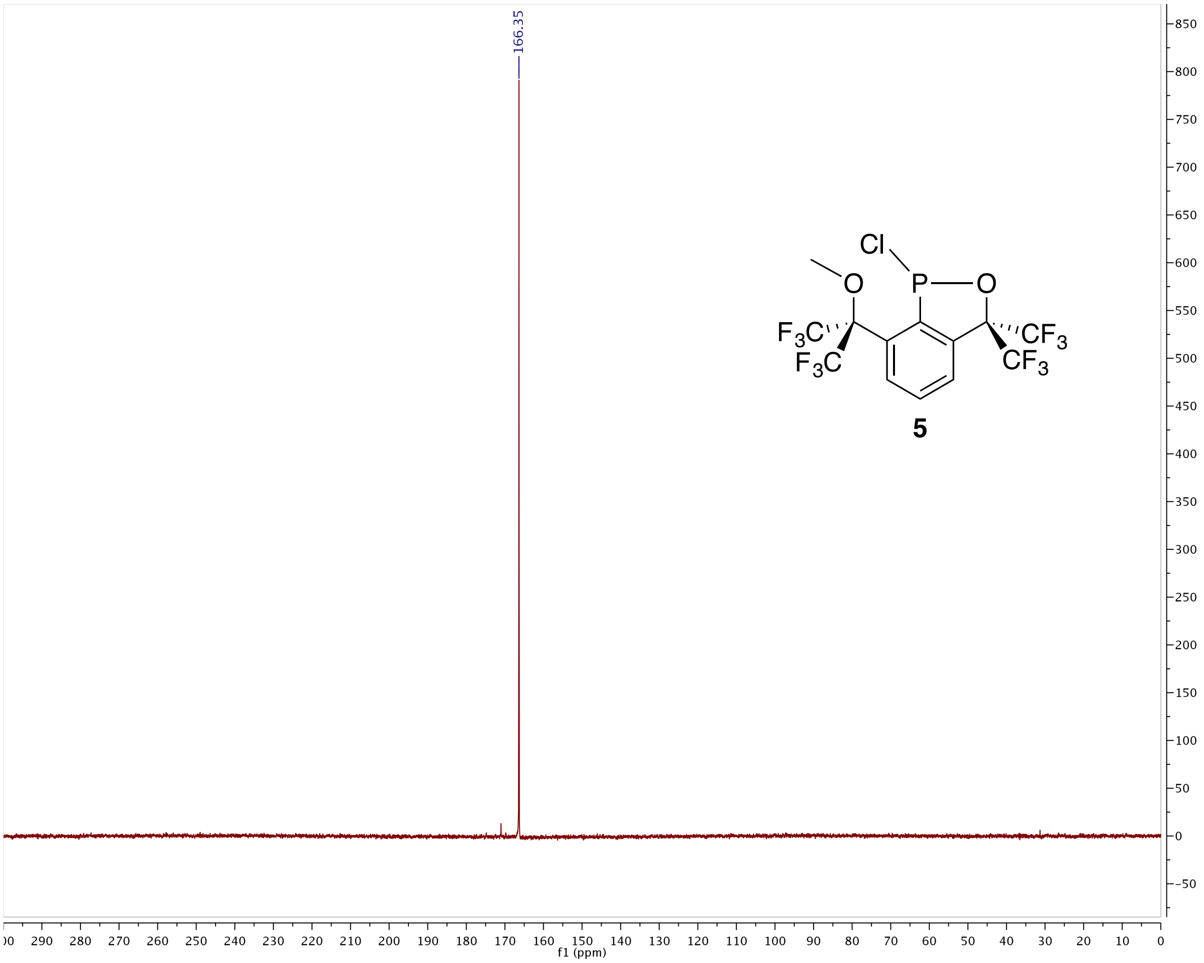

Synthesis of 6.

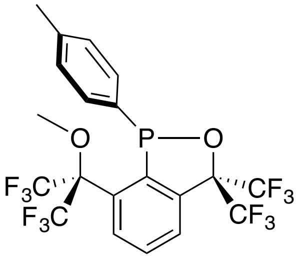

6

Fluorinated aryl bromide **2** (500 mg, 0.967 mmol) was dissolved in 6 mL of THF in a vial with a stir bar inside the glovebox and (*i*-Pr)MgCl·LiCl was added dropwise via syringe (0.82 mL, 1.064 mmol, 1.1 equiv, 1.3 M in THF), resulting in a homogeneous, yellow reaction mixture. The reaction mixture was stirred for 1 h at room temperature (RT) then directly filtered into 2 mL of a pre-cooled solution (-35 °C, 1 h) of PCl_3 (146 mg, 1.064 mmol, 1.1 equiv) in THF. The solution was warmed to RT for 1 h then analyzed by $^{31}P\{^1H\}$ NMR spectroscopy, revealing the presence of unreacted PCl_3 (218 ppm), *i*-$PrPCl_2$ (202 ppm) and the chlorophosphine (170 ppm). The "intermediate" product mixture was then concentrated under vacuum to remove the volatile and unwanted phosphorus byproducts (PCl_3 and *i*-$PrPCl_2$), leaving a pale yellow residue that was dissolved in 6 mL of THF and transferred to a Schlenk bomb fitted with a screw-top Teflon cap. The bomb was taken outside of the glovebox, cooled to 0 °C, and (*p*-CH_3)C_6H_4MgBr (1.06 mL, 1.064 mmol, 1.1 equiv, 1.0 M in THF) was injected via syringe under positive N_2 pressure, affording a light orange reaction mixture, which was subsequently warmed to RT. The Schlenk bomb was resealed (under positive N_2 pressure), brought back inside the glove box, and an aliquot was analyzed by $^{31}P\{^1H\}$ NMR spectroscopy displaying a prominent signal at 129 ppm with slight impurities at -2 and -8 ppm. The entire reaction mixture was concentrated under vacuum, extracted with pentane (3 x 50 mL), and filtered through a Kimwipe plug. The filtrate was concentrated under vacuum, dissolved in ether (2 mL) and cooled to -35 °C overnight, resulting in large white/colorless blocks suitable for X-ray diffraction (202 mg, 0.371 mmol, 38% yield).

Anal. Calcd for $C_{20}H_{13}F_{12}O_2P$: C, 44.14; H, 2.41. Found: C, 44.09; H, 2.41. ^1H NMR (600 MHz, C_6D_6): δ 7.57 (br d, J = 8 Hz, 1H, Ar), 7.52 (d, J = 8 Hz, 1H, Ar), 7.10 (apparent t, J = 8 Hz, 2H, Ar), 6.88 (t, J = 8 Hz, 1H, Ar), 6.81 (d, J = 8 Hz, 2H, Ar), 3.11 (s, 3H, OMe), 1.92 (s, 3H, *p*-Me). $^{13}C\{^1H\}$ NMR (151 MHz, C_6D_6): δ 143.4 (d, J = 33 Hz, Ar), 141.2 (Ar), 137.8 (d, J = 41 Hz, Ar), 135.7 (d, J = 6 Hz, Ar), 133.0 (d, J = 5 Hz, Ar), 132.2 (d, J = 25 Hz, Ar), 131.1 (Ar), 130.0 (Ar), 129.0 (d, J = 8 Hz, Ar), 126.9 (Ar), four overlapping CF_3 signals*: 123.1 (qd, J ~ 290 and 9 Hz), 123.0 (q, J ~ 290 Hz), 122.5 (q, J ~ 290 Hz), and 122.4 (qd, J ~ 290 and 3 Hz), 89.5 (septet of doublets, J = 31.5 and 16.5 Hz, $C(CF_3)_2$ in P-ring), 80.8 (septet, J = 28 Hz, $C(CF_3)_2$), 55.3 (OMe), 21.2 (Me). ^{19}F NMR (282 MHz, C_6D_6):** δ -69.5 (m, J_{FF} ~ 8 Hz, J_{PF} ~ 2 Hz, J_{HF} ~ 0.5-2 Hz), -69.6 (m, J_{FF} ~ 8 Hz, J_{PF} ~ 7 Hz, J_{HF} ~ 0.5-2.5 Hz), -74.0 (m, J_{FF} ~ 9 Hz, J_{HF} ~0.5-1 Hz), and -76.5 (m, J_{FF} ~ 9 Hz, J_{HF} ~1-5 Hz). $^{31}P\{^1H\}$ NMR (121 MHz, C_6D_6): δ 129.4 (septet, J = 6 Hz). *In $CDCl_3$, the overlapping CF_3 signals in the $^{13}C\{^1H\}$ NMR spectrum are better resolved: δ 122.5 (q, J = 290 Hz), 122.3 (qd, J = 290 and 9 Hz), 122.0 (q, J = 290 Hz), 121.6 (qd, J = 290 and 2 Hz). **Reported coupling constants in the ^{19}F NMR spectrum were determined by simulation with Mestrenova.

^{1}H NMR (600 MHz, C_6D_6) spectrum

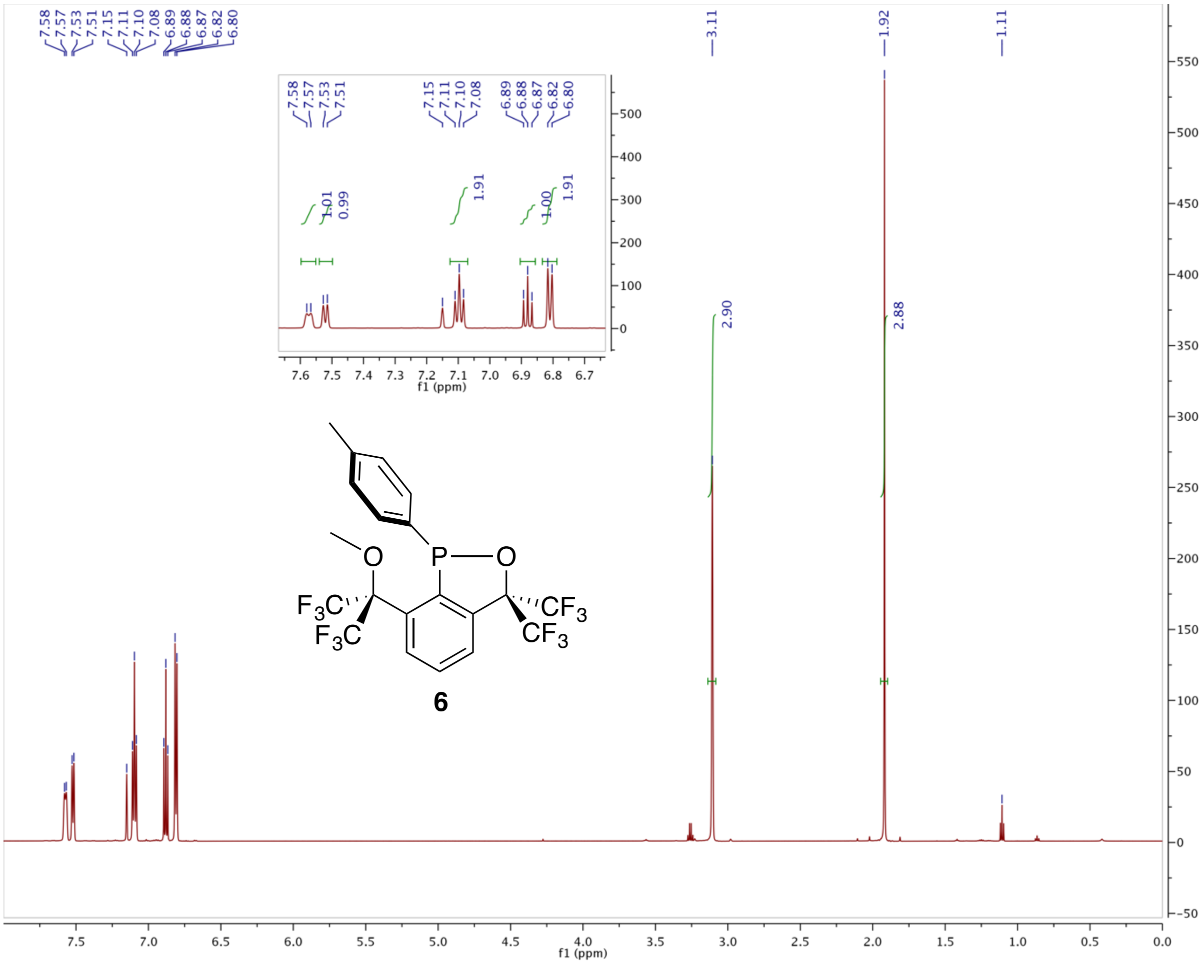

$^{13}C\{^1H\}$ NMR (151 MHz, C_6D_6) spectra

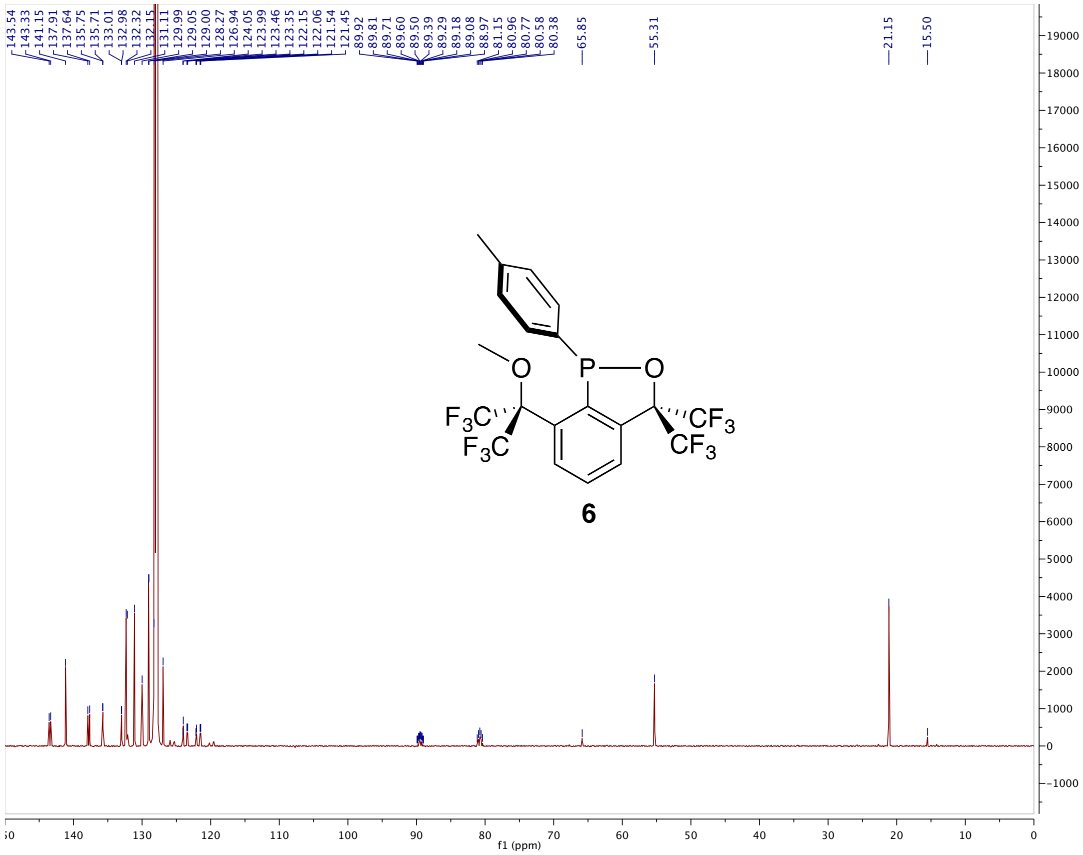

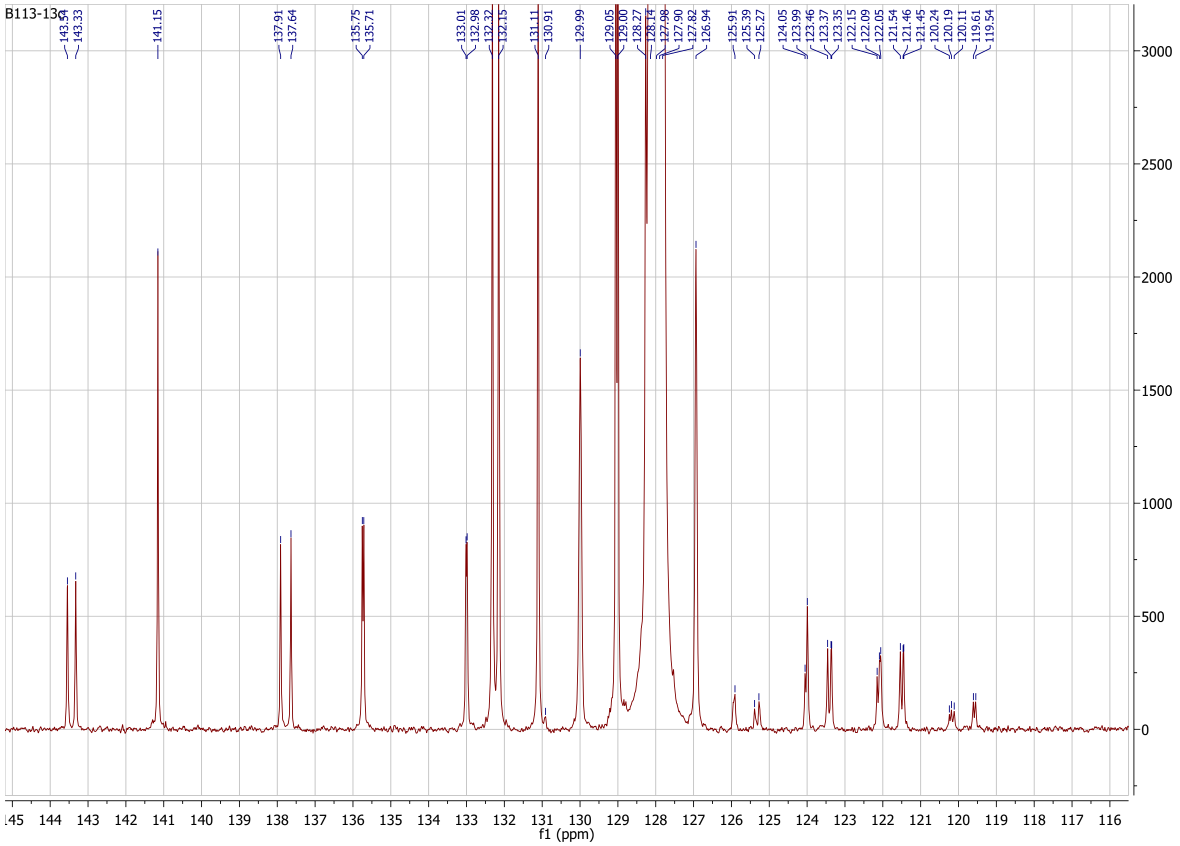
B113-13C
f1 (ppm)

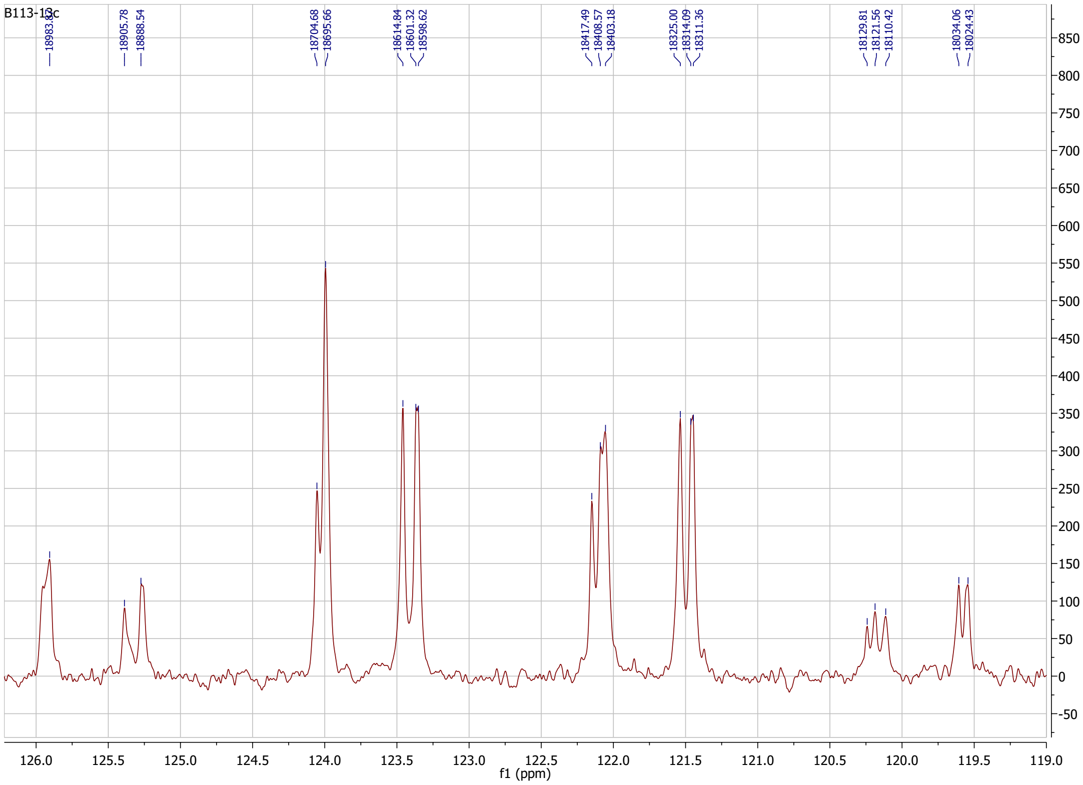

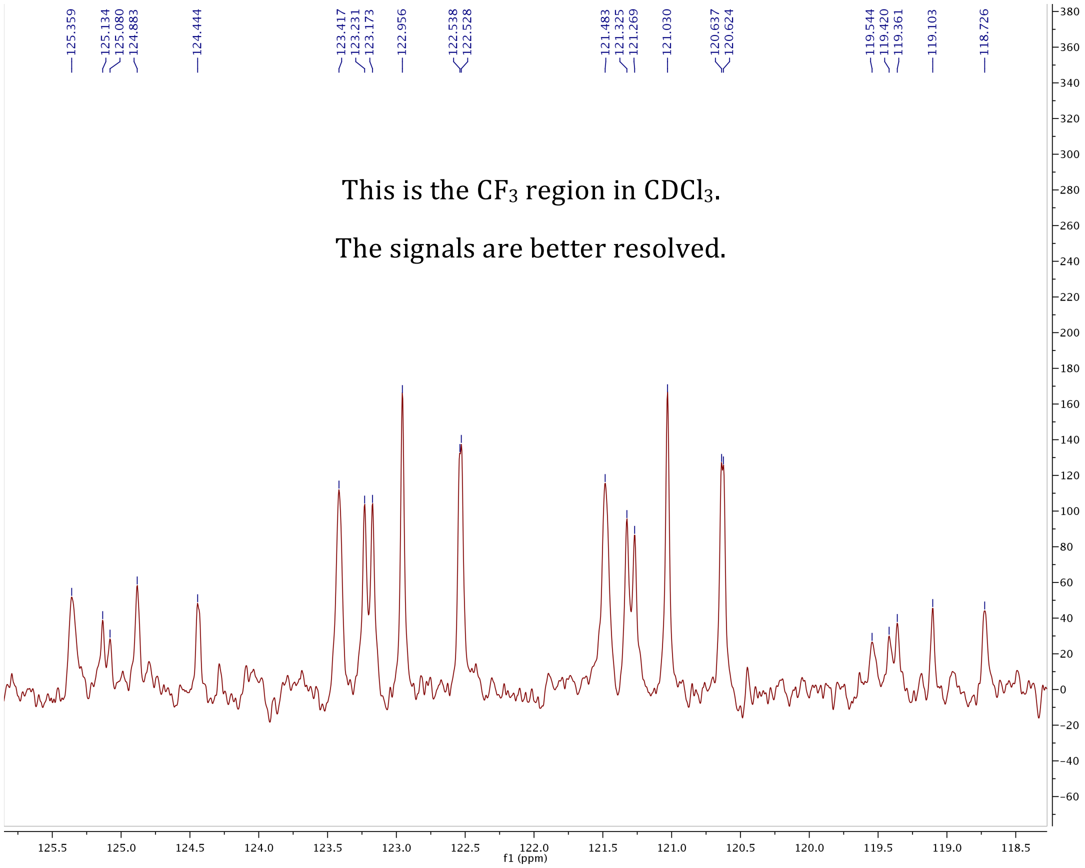

This is the CF_3 region in $CDCl_3$.

The signals are better resolved.

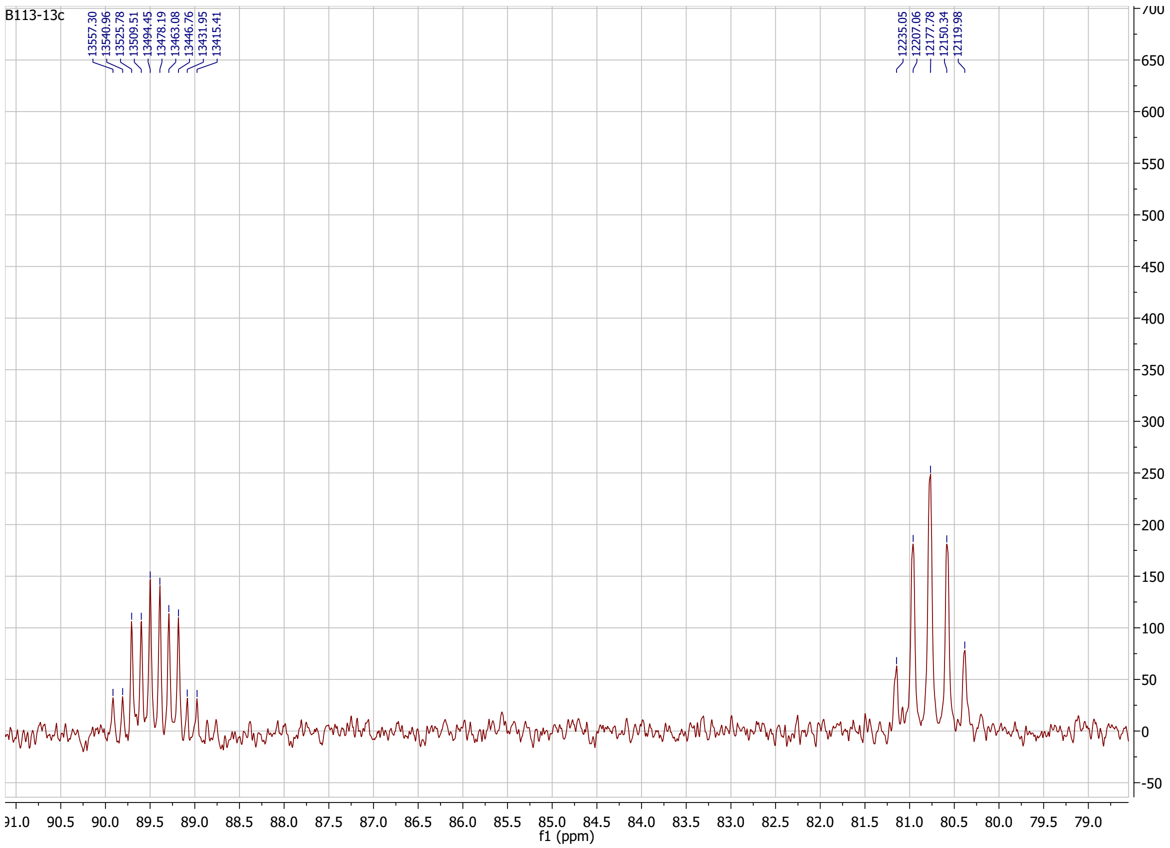
B113-13c
13557.30
13540.96
13525.78
13509.51
13494.45
13478.19
13463.08
13446.76
13431.95
13415.41
12235.05
12207.06
12177.78
12150.34
12119.98
f1 (ppm)

DEPT-135

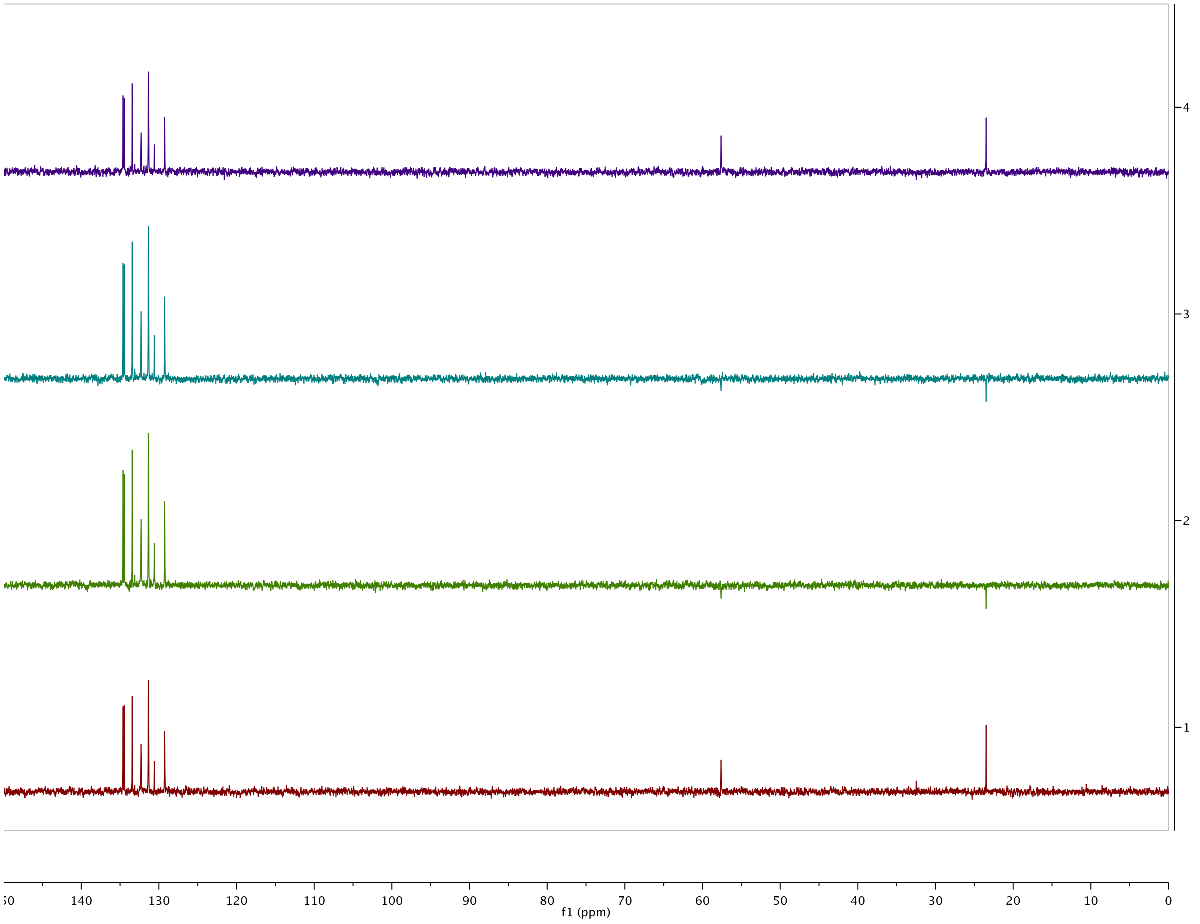

^{19}F NMR (282 MHz, C_6D_6) spectrum

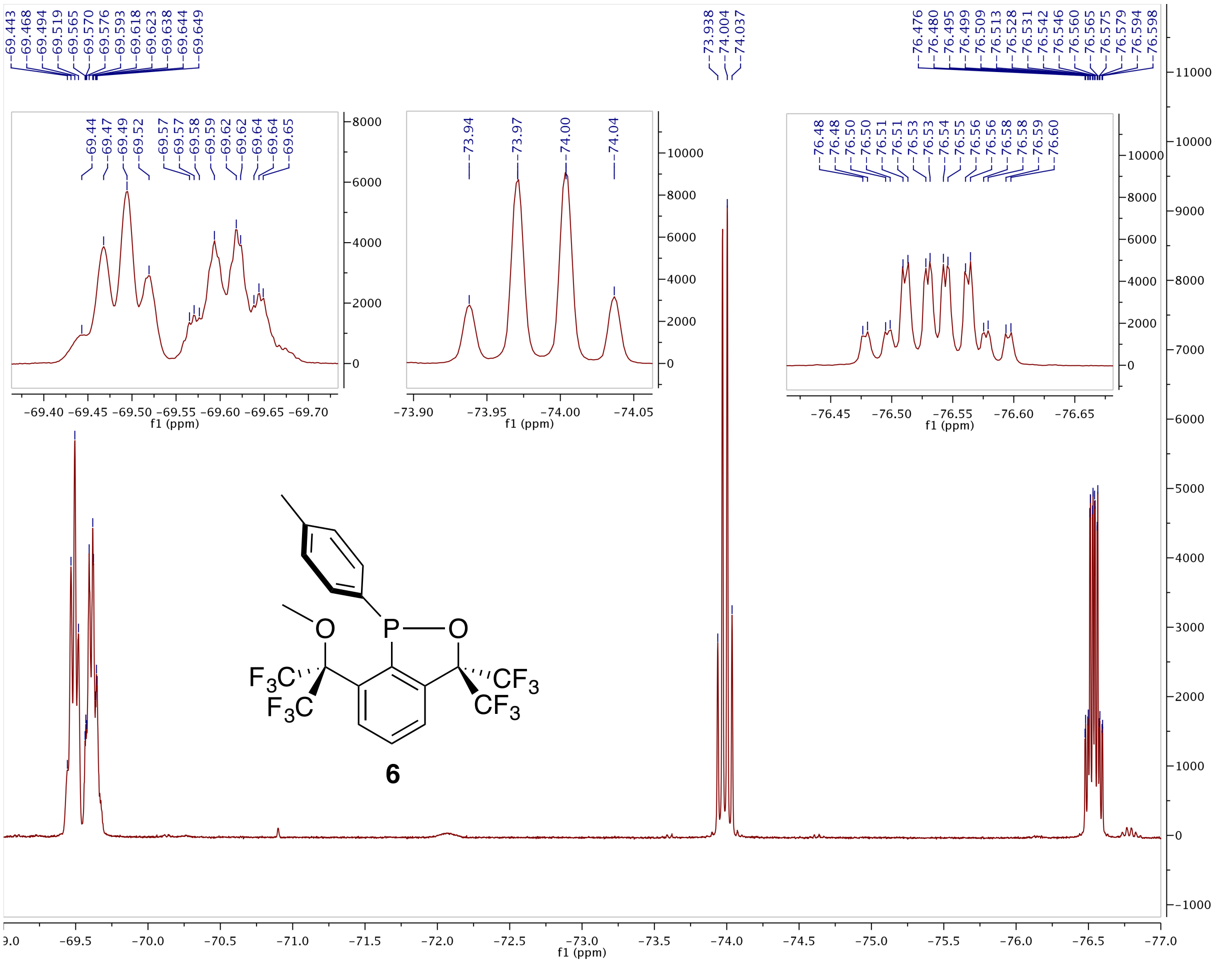

$^{31}P\{^{1}H\}$ NMR (121 MHz, C_6D_6) spectrum

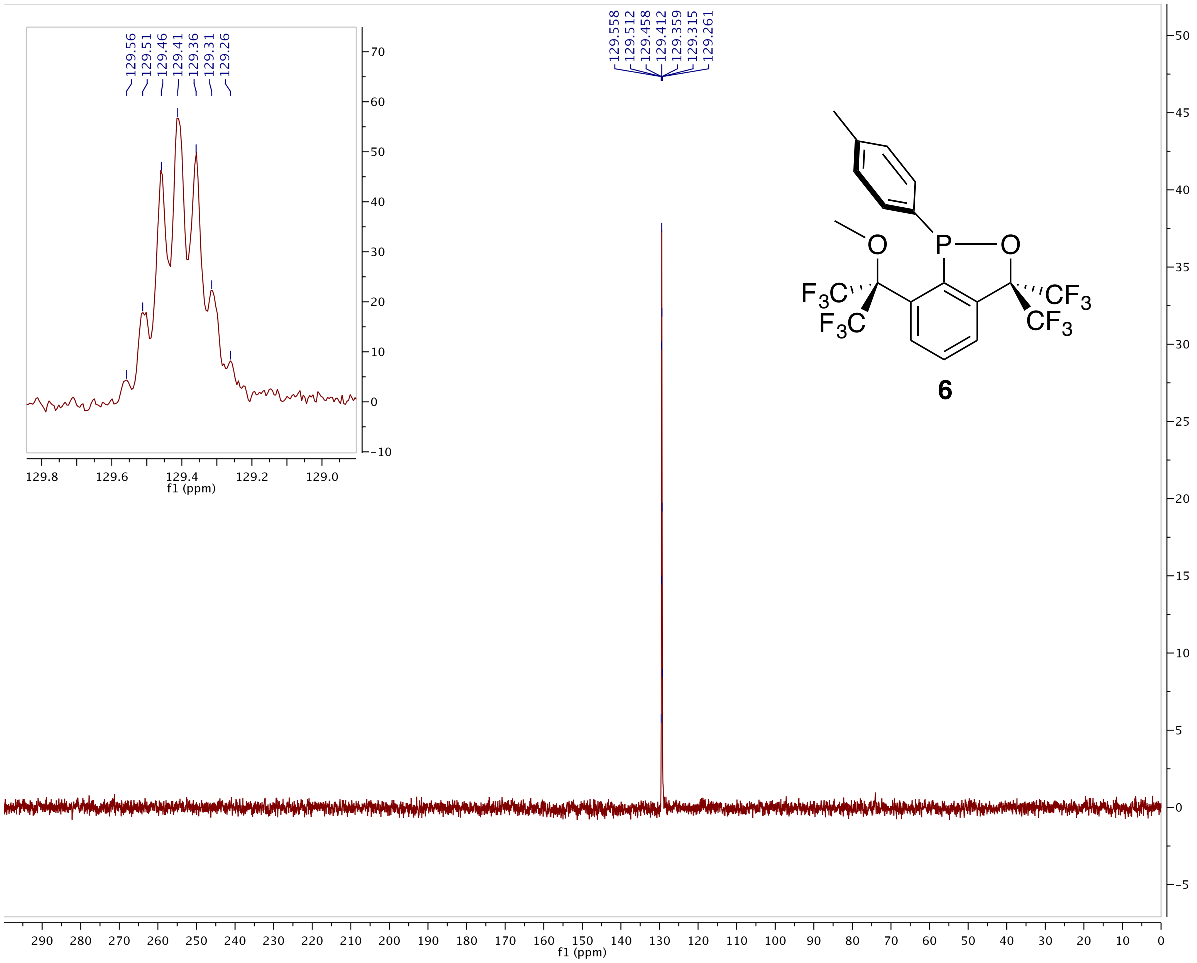

CENTC Elemental Analysis Facility
University of Rochester
Rochester, NY 14627 USA
Email: ealab@chem.rochester.edu

**Date of report**	6/11/2021 4:30:53PM
**User ID**	Administrator
**Comments**	RC B111 [Cain / Chinen]

DATE & TIME	6/11/2021 2:21:07 PM	P_ID	EA LAB
SAMPLE ID	21331	USER ID	Administrator
WEIGHT (mg)	2.006	MODE	CHN

CARBON	44.088%
HYDROGEN	2.405%
NITROGEN	-.095%

Special Handling

The sample was transferred under argon and was combusted in a tin capsule that was crimp-sealed with a die apparatus.

Acknowledgment

Analytical data were obtained from the CENTC Elemental Analysis Facility at the University of Rochester, funded by NSF CHE-0650456.

Instrumentation

Microanalysis samples were weighed with a PerkinElmer Model AD6000 Autobalance and their compositions were determined with a PerkinElmer 2400 Series II Analyzer. Air-sensitive samples were handled in a VAC Atmospheres glovebox.

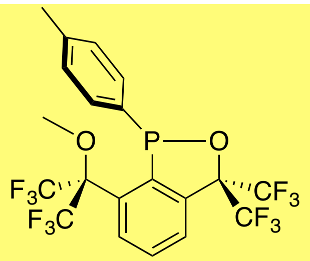

Chemical Formula: $C_{20}H_{13}F_{12}O_2P$
Exact Mass: 544.0462
Molecular Weight: 544.2766
Elemental Analysis: C, 44.14; H, 2.41; F, 41.89; O, 5.88; P, 5.69

2. Simulated ^{19}F NMR Spectra using MestreNova

The ^{19}F spectra for **5** and **6** were simulated with MestreNova. All ^{19}F and ^{31}P spins were included in the simulation along with the aromatic protons. Number of points was matched to the experimental spectrum (16 K to 512 K depending on the experiment). Line widths were set at 0.9 Hz for spectra collected at 282 MHz and 1.1 Hz for spectra collected at 564 MHz. ^{31}P and ^{1}H chemical shifts were set to zero in the ^{19}F simulations. Initial chemical shifts and coupling constants were estimated from the experimental spectrum then iteratively refined until the simulated pattern matched the experimental pattern. After simulating the ^{19}F spectra at 282 MHz the resultant coupling constants were tested against the experimental ^{19}F spectra collected at 564 MHz. The spectra were refined further through slight adjustments to the coupling constants until a suitable match was obtained for the low and high field spectrum.

Table S1. Simulated ^{19}F chemical shifts and coupling constants for **5** at 282 MHz**.**

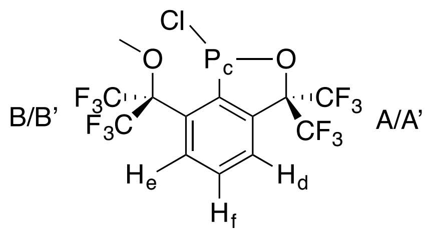

5

^{19}F (ppm)	Label	CF_3 A	CF_3 A'	CF_3 B	CF_3 B'	P_c	H_d	H_e	H_f
-68.9237	CF_3 A								
-70.4093	CF_3 A'	7.93							
-73.3518	CF_3 B								
-76.2722	CF_3 B'			9.34					
	P_c	1.65	3.86	1.9	1.77				
	H_d	1.25	2.58						
	H_e	0.60	0.17	1.22	1.60				
	H_f	1.35	2.25	0.25	0.35		0.10	0.70	

Figure S1. Experimental (red) and simulated (blue) ^{19}F signals at -68.9237 (A), -70.4093 (B), -73.3518 (C) and -76.2722 (D) for **5** at 282 MHz.

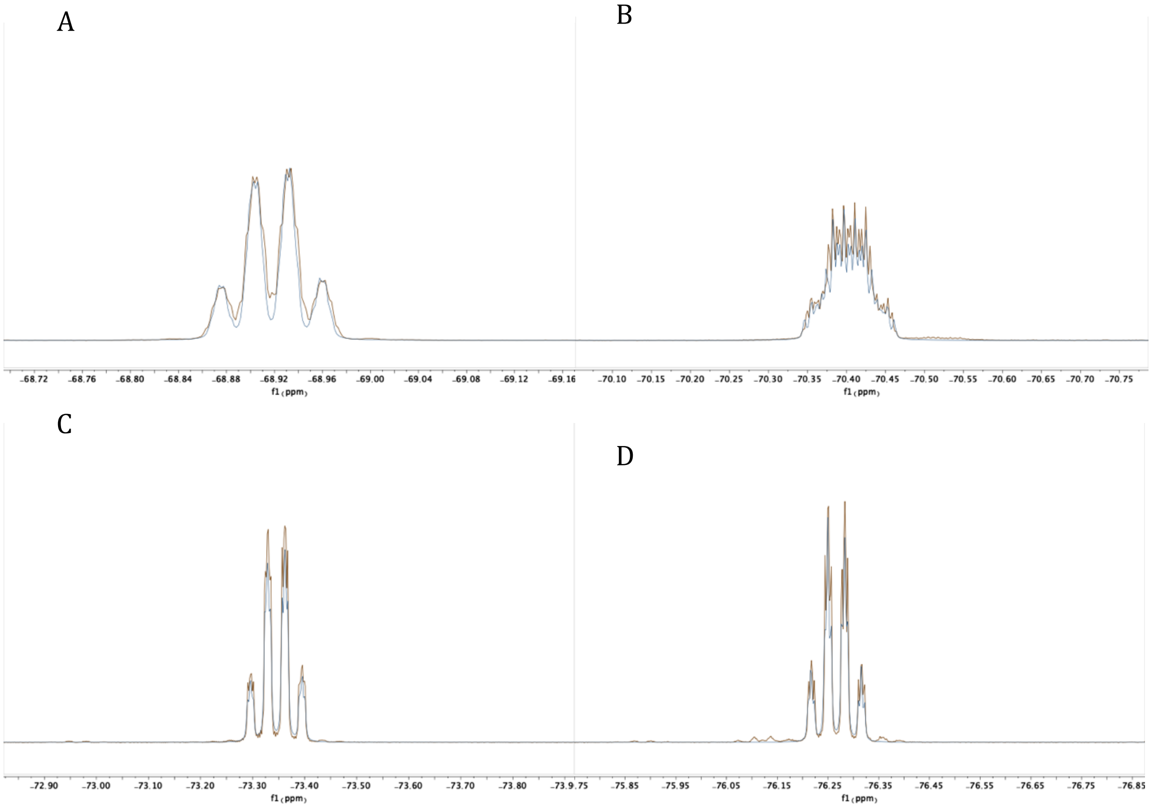

Figure S2. Experimental (red) and simulated (blue) ^{19}F signals at -68.9460 (A), -70.4300 (B), -73.3746 (C) and -76.2950 (D) for **5** at 564 MHz.

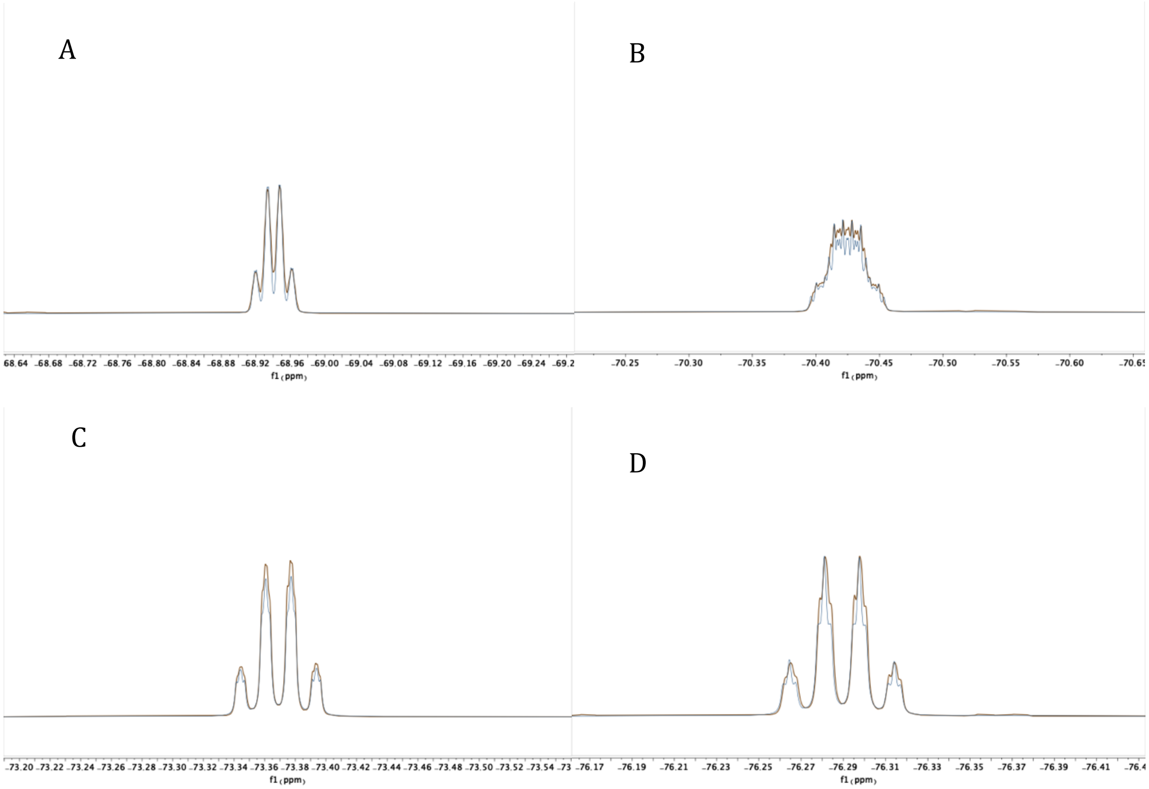

Table S2. Simulated ^{19}F chemical shifts and coupling constants for **6** at 282 MHz.

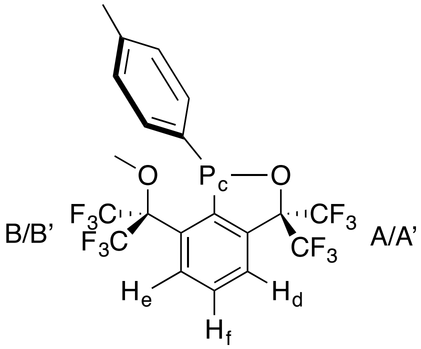

6

^{19}F (ppm)	Label	CF_3 A	CF_3 A'	CF_3 B	CF_3 B'	P_c	H_d	H_e	H_f
-68.4819	CF_3 A								
-69.6176	CF_3 A'	7.90							
-73.9876	CF_3 B								
-76.5368	CF_3 B'			9.30					
	P_c	2.00	7.28						
	H_d	1.65	2.36						
	H_e	0.45	0.54	0.88	5.00				
	H_f	0.9	1.35	0.4	1.30	0.1	0.5	0.5	

Figure S3. Experimental (red) and simulated (blue) ^{19}F signals at -69.4819 and -69.6176 (A), -73.9876 (B), and -76.5368 (C) for **6** at 282 MHz.

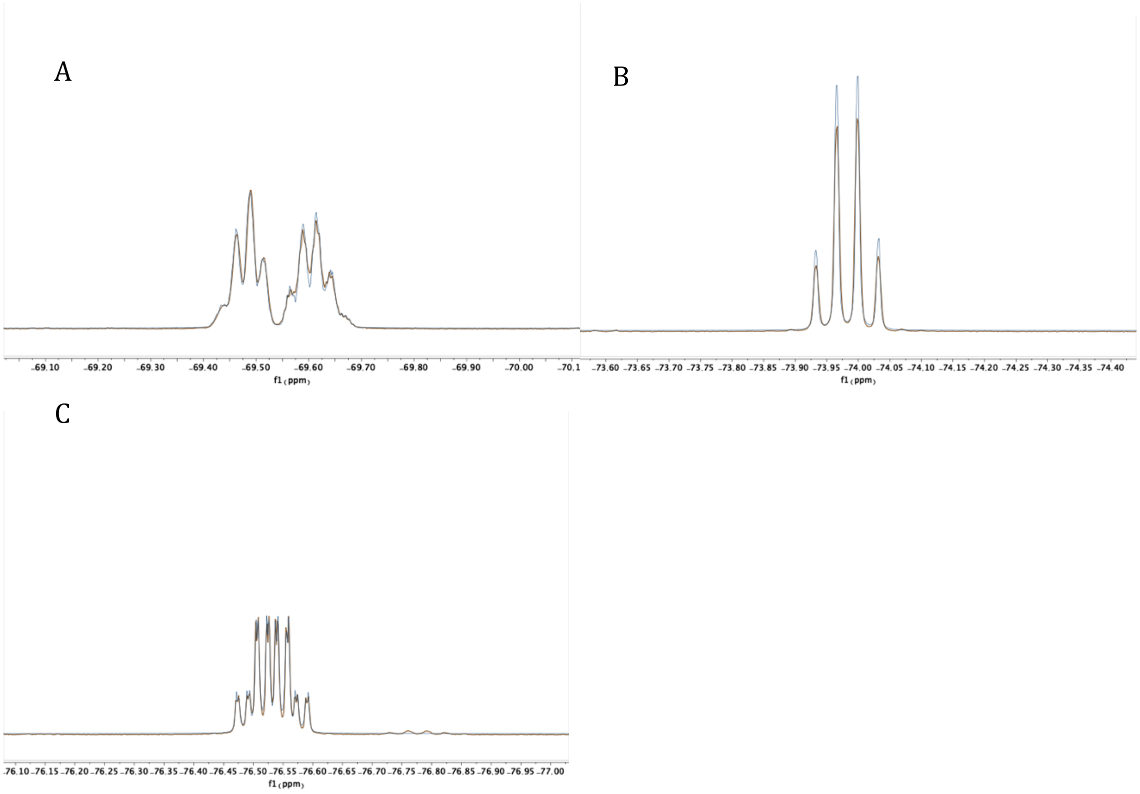

Figure S4. Experimental (red) and simulated (blue) ^{19}F signals at -71.988 and -72.1280 (A), -76.4970 (B), and -79.0490 (C) for **6** at 564 MHz.

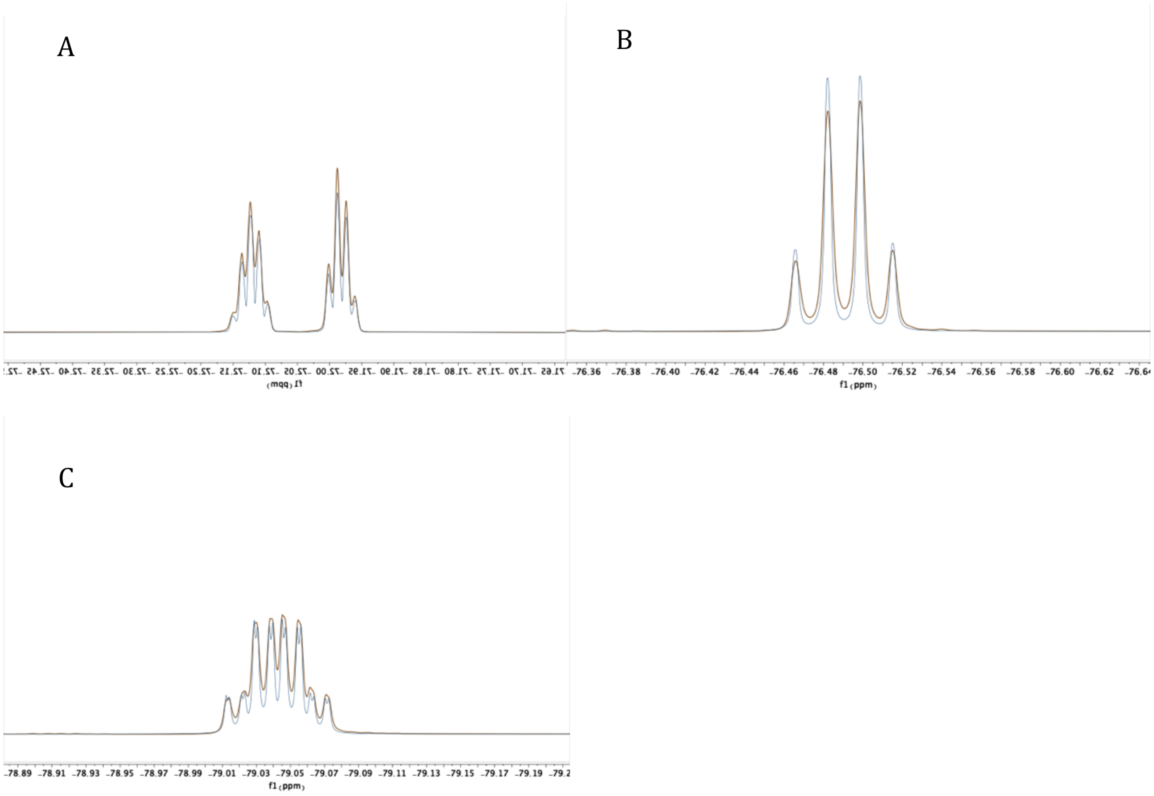

3. Experimental Details for Crystal Structure Refinement and Acquisition

All data were collected on Bruker Micro-Star rotating anode systems using micro-focus optics and APEX detectors. All, except CAIN100 (**6**), were collected using Mo-Kα radiation. Cu-Kα radiation was used for CAIN100 (**6**). CAIN58 (**3**) diffracted only weakly and no high-angle data were available (resolution = 0.86Å). All data sets were processed using current Bruker software and refinement was completed using OLEX2 programs. (1) Pseudo-mirror plane disorder was found in CAIN100 (**6**).

(1) Dolomanov, O.V.; Bourhis, L.J.; Gildea, R.J.; Howard, J.A.K.; Puschmann, H., OLEX2: A complete structure solution, refinement and analysis program (2009). J. Appl. Cryst., 42, 339-341.

X-RAY OF 1

Table 1. Crystal data and structure refinement for cain57_0m_a (**1**).

Identification code	JH154	
Empirical formula	C26 H29 Br O2	
Formula weight	453.40	
Temperature	100.0 K	
Wavelength	0.71073 Å	
Crystal system	Triclinic	
Space group	P-1	
Unit cell dimensions	a = 7.4093(13) Å	α= 101.293(4)°.
	b = 8.2707(14) Å	β= 100.471(5)°.
	c = 18.706(3) Å	γ = 94.032(6)°.
Volume	1098.7(3) $Å^3$	
Z	2	
Density (calculated)	1.371 Mg/m^3	
Absorption coefficient	1.890 mm^{-1}	
F(000)	472	
Crystal size	0.32 x 0.3 x 0.28 mm^3	
Theta range for data collection	2.813 to 25.372°.	
Index ranges	-8<=h<=8, -8<=k<=9, -22<=l<=22	
Reflections collected	11445	
Independent reflections	3990 [R(int) = 0.0341]	
Completeness to theta = 25.242°	99.5 %	
Absorption correction	Semi-empirical from equivalents	
Max. and min. transmission	0.2590 and 0.2112	
Refinement method	Full-matrix least-squares on F^2	
Data / restraints / parameters	3990 / 0 / 268	
Goodness-of-fit on F^2	1.092	
Final R indices [I>2sigma(I)]	R1 = 0.0465, wR2 = 0.1194	
R indices (all data)	R1 = 0.0553, wR2 = 0.1231	
Extinction coefficient	n/a	
Largest diff. peak and hole	2.092 and -0.456 e.$Å^{-3}$	

Table 2. Atomic coordinates (x 10^4) and equivalent isotropic displacement parameters ($Å^2$x 10^3) for cain57_0m_a (**1**). U(eq) is defined as one third of the trace of the orthogonalized U^{ij} tensor.

	x	y	z	U(eq)
Br(1)	2058(1)	1429(1)	5060(1)	22(1)
O(1)	3198(3)	4135(3)	6829(1)	17(1)
O(2)	1719(3)	2549(3)	3248(1)	18(1)
C(9)	4142(5)	3949(5)	7521(2)	17(1)
C(18)	838(5)	1614(4)	2553(2)	17(1)
C(13)	5742(5)	5178(5)	8762(2)	24(1)
C(24)	386(5)	4310(5)	2127(2)	23(1)
C(14)	4820(5)	5373(5)	8068(2)	19(1)
C(10)	4363(5)	2357(5)	7652(2)	20(1)
C(17)	4616(5)	7078(5)	7915(2)	24(1)
C(19)	193(5)	2451(5)	1989(2)	19(1)
C(2)	3472(5)	4811(4)	5661(2)	14(1)
C(12)	6002(5)	3620(5)	8905(2)	26(1)
C(21)	-882(5)	-224(5)	1154(2)	24(1)
C(6)	1519(5)	4236(4)	4410(2)	15(1)
C(5)	1739(5)	5923(4)	4417(2)	18(1)
C(20)	-663(5)	1495(5)	1296(2)	23(1)
C(23)	678(5)	-109(5)	2425(2)	20(1)
C(8)	438(5)	3069(4)	3723(2)	18(1)
C(11)	5307(5)	2227(5)	8346(2)	25(1)
C(7)	2378(5)	3714(4)	5038(2)	14(1)
C(3)	3678(5)	6474(4)	5627(2)	17(1)
C(15)	3635(6)	824(5)	7058(2)	27(1)
C(1)	4457(5)	4257(4)	6333(2)	16(1)
C(4)	2820(5)	7032(4)	5021(2)	20(1)
C(22)	-183(6)	-1003(5)	1723(2)	26(1)
C(26)	1386(6)	-1006(5)	3031(2)	28(1)
C(25)	-1889(6)	-1229(6)	403(2)	37(1)
C(16)	7060(6)	3434(6)	9649(2)	37(1)

Table 3. Bond lengths [Å] and angles [°] for cain57_0m_a (**1**).

Br(1)-C(7)	1.897(3)
O(1)-C(9)	1.397(4)
O(1)-C(1)	1.443(4)
O(2)-C(18)	1.393(4)
O(2)-C(8)	1.445(4)
C(9)-C(14)	1.395(5)
C(9)-C(10)	1.401(5)
C(18)-C(19)	1.403(5)
C(18)-C(23)	1.391(5)
C(13)-H(13)	0.9500
C(13)-C(14)	1.396(5)
C(13)-C(12)	1.386(6)
C(24)-H(24A)	0.9800
C(24)-H(24B)	0.9800
C(24)-H(24C)	0.9800
C(24)-C(19)	1.501(5)
C(14)-C(17)	1.506(5)
C(10)-C(11)	1.387(5)
C(10)-C(15)	1.506(5)
C(17)-H(17A)	0.9800
C(17)-H(17B)	0.9800
C(17)-H(17C)	0.9800
C(19)-C(20)	1.391(5)
C(2)-C(7)	1.406(5)
C(2)-C(3)	1.389(5)
C(2)-C(1)	1.503(5)
C(12)-C(11)	1.391(6)
C(12)-C(16)	1.510(5)
C(21)-C(20)	1.387(5)
C(21)-C(22)	1.389(6)
C(21)-C(25)	1.516(5)
C(6)-C(5)	1.390(5)
C(6)-C(8)	1.502(5)
C(6)-C(7)	1.392(5)

C(5)-H(5)	0.9500
C(5)-C(4)	1.386(5)
C(20)-H(20)	0.9500
C(23)-C(22)	1.388(5)
C(23)-C(26)	1.510(5)
C(8)-H(8A)	0.9900
C(8)-H(8B)	0.9900
C(11)-H(11)	0.9500
C(3)-H(3)	0.9500
C(3)-C(4)	1.374(5)
C(15)-H(15A)	0.9800
C(15)-H(15B)	0.9800
C(15)-H(15C)	0.9800
C(1)-H(1A)	0.9900
C(1)-H(1B)	0.9900
C(4)-H(4)	0.9500
C(22)-H(22)	0.9500
C(26)-H(26A)	0.9800
C(26)-H(26B)	0.9800
C(26)-H(26C)	0.9800
C(25)-H(25A)	0.9800
C(25)-H(25B)	0.9800
C(25)-H(25C)	0.9800
C(16)-H(16A)	0.9800
C(16)-H(16B)	0.9800
C(16)-H(16C)	0.9800

C(9)-O(1)-C(1)	110.7(2)
C(18)-O(2)-C(8)	112.6(3)
O(1)-C(9)-C(10)	119.7(3)
C(14)-C(9)-O(1)	118.4(3)
C(14)-C(9)-C(10)	121.8(3)
O(2)-C(18)-C(19)	118.5(3)
C(23)-C(18)-O(2)	119.8(3)
C(23)-C(18)-C(19)	121.7(3)
C(14)-C(13)-H(13)	119.3

C(12)-C(13)-H(13)	119.3
C(12)-C(13)-C(14)	121.4(4)
H(24A)-C(24)-H(24B)	109.5
H(24A)-C(24)-H(24C)	109.5
H(24B)-C(24)-H(24C)	109.5
C(19)-C(24)-H(24A)	109.5
C(19)-C(24)-H(24B)	109.5
C(19)-C(24)-H(24C)	109.5
C(9)-C(14)-C(13)	118.1(3)
C(9)-C(14)-C(17)	121.3(3)
C(13)-C(14)-C(17)	120.6(3)
C(9)-C(10)-C(15)	121.6(3)
C(11)-C(10)-C(9)	117.9(3)
C(11)-C(10)-C(15)	120.4(3)
C(14)-C(17)-H(17A)	109.5
C(14)-C(17)-H(17B)	109.5
C(14)-C(17)-H(17C)	109.5
H(17A)-C(17)-H(17B)	109.5
H(17A)-C(17)-H(17C)	109.5
H(17B)-C(17)-H(17C)	109.5
C(18)-C(19)-C(24)	121.5(3)
C(20)-C(19)-C(18)	117.7(3)
C(20)-C(19)-C(24)	120.8(3)
C(7)-C(2)-C(1)	123.3(3)
C(3)-C(2)-C(7)	116.9(3)
C(3)-C(2)-C(1)	119.7(3)
C(13)-C(12)-C(11)	118.9(4)
C(13)-C(12)-C(16)	120.7(4)
C(11)-C(12)-C(16)	120.4(4)
C(20)-C(21)-C(22)	118.4(3)
C(20)-C(21)-C(25)	120.8(4)
C(22)-C(21)-C(25)	120.8(4)
C(5)-C(6)-C(8)	119.0(3)
C(5)-C(6)-C(7)	117.6(3)
C(7)-C(6)-C(8)	123.4(3)
C(6)-C(5)-H(5)	119.5

C(4)-C(5)-C(6)	121.0(3)
C(4)-C(5)-H(5)	119.5
C(19)-C(20)-H(20)	118.9
C(21)-C(20)-C(19)	122.1(4)
C(21)-C(20)-H(20)	118.9
C(18)-C(23)-C(26)	121.6(3)
C(22)-C(23)-C(18)	118.3(3)
C(22)-C(23)-C(26)	120.1(3)
O(2)-C(8)-C(6)	107.5(3)
O(2)-C(8)-H(8A)	110.2
O(2)-C(8)-H(8B)	110.2
C(6)-C(8)-H(8A)	110.2
C(6)-C(8)-H(8B)	110.2
H(8A)-C(8)-H(8B)	108.5
C(10)-C(11)-C(12)	121.8(4)
C(10)-C(11)-H(11)	119.1
C(12)-C(11)-H(11)	119.1
C(2)-C(7)-Br(1)	118.3(3)
C(6)-C(7)-Br(1)	118.9(3)
C(6)-C(7)-C(2)	122.8(3)
C(2)-C(3)-H(3)	119.2
C(4)-C(3)-C(2)	121.7(3)
C(4)-C(3)-H(3)	119.2
C(10)-C(15)-H(15A)	109.5
C(10)-C(15)-H(15B)	109.5
C(10)-C(15)-H(15C)	109.5
H(15A)-C(15)-H(15B)	109.5
H(15A)-C(15)-H(15C)	109.5
H(15B)-C(15)-H(15C)	109.5
O(1)-C(1)-C(2)	109.0(3)
O(1)-C(1)-H(1A)	109.9
O(1)-C(1)-H(1B)	109.9
C(2)-C(1)-H(1A)	109.9
C(2)-C(1)-H(1B)	109.9
H(1A)-C(1)-H(1B)	108.3
C(5)-C(4)-H(4)	120.0

C(3)-C(4)-C(5)	120.1(3)
C(3)-C(4)-H(4)	120.0
C(21)-C(22)-H(22)	119.1
C(23)-C(22)-C(21)	121.8(4)
C(23)-C(22)-H(22)	119.1
C(23)-C(26)-H(26A)	109.5
C(23)-C(26)-H(26B)	109.5
C(23)-C(26)-H(26C)	109.5
H(26A)-C(26)-H(26B)	109.5
H(26A)-C(26)-H(26C)	109.5
H(26B)-C(26)-H(26C)	109.5
C(21)-C(25)-H(25A)	109.5
C(21)-C(25)-H(25B)	109.5
C(21)-C(25)-H(25C)	109.5
H(25A)-C(25)-H(25B)	109.5
H(25A)-C(25)-H(25C)	109.5
H(25B)-C(25)-H(25C)	109.5
C(12)-C(16)-H(16A)	109.5
C(12)-C(16)-H(16B)	109.5
C(12)-C(16)-H(16C)	109.5
H(16A)-C(16)-H(16B)	109.5
H(16A)-C(16)-H(16C)	109.5
H(16B)-C(16)-H(16C)	109.5

Symmetry transformations used to generate equivalent atoms:

Table 4. Anisotropic displacement parameters ($Å^2 x 10^3$) for cain57_0m_a (**1**). The anisotropic displacement factor exponent takes the form: $-2\pi^2[h^2 a^{*2}U^{11} + ... + 2 h k a^* b^* U^{12}]$

	U^{11}	U^{22}	U^{33}	U^{23}	U^{13}	U^{12}
Br(1)	33(1)	12(1)	20(1)	4(1)	1(1)	-1(1)
O(1)	15(1)	24(1)	13(1)	7(1)	3(1)	2(1)
O(2)	14(1)	24(1)	14(1)	0(1)	3(1)	4(1)
C(9)	13(2)	26(2)	13(2)	4(1)	3(1)	2(1)
C(18)	12(2)	25(2)	11(2)	-2(1)	1(1)	-1(1)
C(13)	20(2)	35(2)	16(2)	2(2)	4(2)	2(2)
C(24)	24(2)	24(2)	22(2)	6(2)	4(2)	2(2)
C(14)	16(2)	24(2)	17(2)	3(2)	6(2)	4(2)
C(10)	20(2)	23(2)	20(2)	5(2)	7(2)	3(2)
C(17)	25(2)	21(2)	24(2)	0(2)	6(2)	2(2)
C(19)	16(2)	24(2)	17(2)	5(2)	6(2)	3(2)
C(2)	12(2)	16(2)	14(2)	1(1)	5(1)	2(1)
C(12)	22(2)	41(2)	17(2)	9(2)	4(2)	5(2)
C(21)	23(2)	29(2)	18(2)	-4(2)	6(2)	-3(2)
C(6)	10(2)	21(2)	16(2)	3(1)	6(1)	3(1)
C(5)	20(2)	24(2)	15(2)	8(2)	6(2)	8(2)
C(20)	24(2)	31(2)	14(2)	5(2)	3(2)	5(2)
C(23)	21(2)	23(2)	20(2)	6(2)	8(2)	4(2)
C(8)	14(2)	25(2)	14(2)	4(2)	2(1)	3(1)
C(11)	28(2)	27(2)	24(2)	12(2)	7(2)	7(2)
C(7)	15(2)	10(2)	17(2)	2(1)	7(1)	2(1)
C(3)	20(2)	15(2)	18(2)	0(1)	7(2)	1(1)
C(15)	31(2)	21(2)	28(2)	5(2)	6(2)	-1(2)
C(1)	14(2)	18(2)	16(2)	3(1)	4(1)	1(1)
C(4)	24(2)	14(2)	25(2)	6(2)	10(2)	4(2)
C(22)	33(2)	19(2)	24(2)	-2(2)	10(2)	0(2)
C(26)	36(2)	28(2)	23(2)	9(2)	6(2)	12(2)
C(25)	39(3)	42(3)	21(2)	-7(2)	1(2)	-5(2)
C(16)	37(3)	51(3)	23(2)	16(2)	-1(2)	6(2)

Table 5. Hydrogen coordinates (x 10^4) and isotropic displacement parameters ($Å^2$x 10^3) for cain57_0m_a (**1**).

	x	y	z	U(eq)
H(13)	6202	6133	9143	29
H(24A)	-582	4731	2383	35
H(24B)	264	4661	1651	35
H(24C)	1600	4753	2436	35
H(17A)	5573	7379	7651	36
H(17B)	4746	7883	8386	36
H(17C)	3395	7082	7608	36
H(5)	1139	6320	4002	22
H(20)	-1112	2038	907	27
H(8A)	-164	2095	3857	21
H(8B)	-530	3634	3463	21
H(11)	5483	1158	8443	30
H(3)	4431	7246	6033	21
H(15A)	2684	1098	6676	40
H(15B)	3100	-26	7280	40
H(15C)	4648	403	6830	40
H(1A)	4900	3166	6178	19
H(1B)	5540	5066	6590	19
H(4)	2967	8180	5015	24
H(22)	-298	-2181	1629	31
H(26A)	2231	-233	3438	42
H(26B)	2042	-1924	2830	42
H(26C)	343	-1444	3219	42
H(25A)	-2900	-640	202	55
H(25B)	-2391	-2314	459	55
H(25C)	-1026	-1381	61	55
H(16A)	8368	3818	9700	56
H(16B)	6932	2264	9682	56
H(16C)	6566	4098	10049	56

X-RAY OF "HALF TRIFLUOROMETHYLATED"

Table 1. Crystal data and structure refinement for cain80_0m_a (**"HALF"**).

Identification code	RC.BIZ	
Empirical formula	C11 H7 Br F6 O3	
Formula weight	381.08	
Temperature	100.0 K	
Wavelength	0.71073 Å	
Crystal system	Monoclinic	
Space group	C c	
Unit cell dimensions	a = 13.9233(7) Å	α= 90°.
	b = 14.4415(9) Å	β= 100.826(2)°.
	c = 12.9349(7) Å	γ = 90°.
Volume	2554.6(2) $Å^3$	
Z	8	
Density (calculated)	1.982 Mg/m^3	
Absorption coefficient	3.300 mm^{-1}	
F(000)	1488	
Crystal size	0.32 x 0.31 x 0.28 mm^3	
Theta range for data collection	2.051 to 28.287°.	
Index ranges	-16<=h<=18, -19<=k<=16, -16<=l<=17	
Reflections collected	8847	
Independent reflections	4616 [R(int) = 0.0348]	
Completeness to theta = 25.000°	99.9 %	
Absorption correction	Semi-empirical from equivalents	
Max. and min. transmission	0.7457 and 0.5761	
Refinement method	Full-matrix least-squares on F^2	
Data / restraints / parameters	4616 / 2 / 386	
Goodness-of-fit on F^2	0.833	
Final R indices [I>2sigma(I)]	R1 = 0.0230, wR2 = 0.0533	
R indices (all data)	R1 = 0.0251, wR2 = 0.0541	
Absolute structure parameter	0.007(5)	
Extinction coefficient	n/a	
Largest diff. peak and hole	0.414 and -0.312 e.$Å^{-3}$	

Table 2. Atomic coordinates (x 10^4) and equivalent isotropic displacement parameters (Å^{2}x 10^3) for cain80_0m_a (**“HALF”**). U(eq) is defined as one third of the trace of the orthogonalized U^{ij} tensor.

	x	y	z	U(eq)
Br(1')	5669(1)	5628(1)	6811(1)	14(1)
Br(1)	5256(1)	1266(1)	6661(1)	14(1)
F(1)	5475(2)	-755(2)	5767(2)	19(1)
F(4')	2685(2)	6101(2)	4523(2)	23(1)
F(2')	2736(2)	4670(2)	3218(2)	23(1)
F(4)	3386(2)	-766(2)	3017(2)	23(1)
F(6')	3417(2)	5943(2)	6139(2)	23(1)
O(1')	4166(2)	4280(2)	5760(2)	14(1)
F(6)	2601(2)	365(2)	3590(2)	21(1)
F(3)	4336(2)	-1719(2)	5146(2)	23(1)
O(1)	3687(2)	-25(2)	5679(2)	14(1)
F(1')	4032(2)	3812(2)	3564(2)	22(1)
O(3)	5924(2)	3525(2)	5804(2)	20(1)
F(2)	5198(2)	-1092(2)	4116(2)	23(1)
O(3')	7750(2)	6609(2)	5833(2)	18(1)
F(3')	2821(2)	3569(2)	4340(2)	28(1)
F(5)	2584(2)	-996(2)	4257(2)	24(1)
O(2)	7081(2)	2437(2)	6001(2)	16(1)
O(2')	6683(2)	7612(2)	6297(2)	15(1)
F(5')	2288(2)	4985(2)	5464(2)	32(1)
C(10)	6211(2)	2765(2)	5604(2)	12(1)
C(11')	7487(2)	8046(3)	7012(3)	19(1)
C(11)	7693(2)	3018(3)	6772(3)	19(1)
C(10')	6923(2)	6889(2)	5762(2)	12(1)
C(5')	5403(2)	5886(2)	5345(2)	11(1)
C(9)	4632(2)	886(3)	3376(3)	14(1)
C(8)	5130(2)	1629(3)	3045(3)	15(1)
C(3)	4774(3)	-937(3)	4945(3)	16(1)
C(4')	4622(2)	5516(2)	4612(3)	10(1)
C(5)	5130(2)	1355(2)	5178(2)	10(1)
C(4)	4602(2)	738(2)	4441(2)	10(1)

C(6)	5633(2)	2104(2)	4833(3)	12(1)
C(9')	4529(2)	5805(2)	3567(3)	14(1)
C(2')	3835(2)	4872(2)	4916(3)	12(1)
C(7')	5938(2)	6781(3)	3974(3)	15(1)
C(6')	6061(2)	6507(2)	5014(3)	11(1)
C(8')	5169(3)	6434(3)	3242(3)	16(1)
C(3')	3339(3)	4235(3)	3989(3)	18(1)
C(1')	3041(3)	5484(3)	5266(3)	18(1)
C(2)	4052(2)	-115(2)	4749(2)	12(1)
C(1)	3148(3)	-381(3)	3887(3)	17(1)
C(7)	5624(2)	2247(2)	3776(3)	14(1)

Table 3. Bond lengths [Å] and angles [°] for cain80_0m_a (**"HALF"**).

Br(1')-C(5')	1.900(3)
Br(1)-C(5)	1.898(3)
F(1)-C(3)	1.327(4)
F(4')-C(1')	1.336(4)
F(2')-C(3')	1.334(4)
F(4)-C(1)	1.352(4)
F(6')-C(1')	1.330(4)
O(1')-H(1')	0.8400
O(1')-C(2')	1.395(4)
F(6)-C(1)	1.333(4)
F(3)-C(3)	1.332(4)
O(1)-C(2)	1.396(4)
O(1)-H(1)	0.78(4)
F(1')-C(3')	1.344(4)
O(3)-C(10)	1.211(4)
F(2)-C(3)	1.336(4)
O(3')-C(10')	1.208(4)
F(3')-C(3')	1.333(4)
F(5)-C(1)	1.332(4)
O(2)-C(10)	1.312(4)
O(2)-C(11)	1.451(4)
O(2')-C(11')	1.453(4)
O(2')-C(10')	1.329(4)
F(5')-C(1')	1.336(4)
C(10)-C(6)	1.502(5)
C(11')-H(11A)	0.9800
C(11')-H(11B)	0.9800
C(11')-H(11C)	0.9800
C(11)-H(11D)	0.9800
C(11)-H(11E)	0.9800
C(11)-H(11F)	0.9800
C(10')-C(6')	1.498(5)
C(5')-C(4')	1.407(5)
C(5')-C(6')	1.405(4)

C(9)-H(9)	0.9500
C(9)-C(8)	1.388(5)
C(9)-C(4)	1.403(4)
C(8)-H(8)	0.9500
C(8)-C(7)	1.385(5)
C(3)-C(2)	1.546(5)
C(4')-C(9')	1.396(4)
C(4')-C(2')	1.544(4)
C(5)-C(4)	1.408(5)
C(5)-C(6)	1.406(5)
C(4)-C(2)	1.542(4)
C(6)-C(7)	1.380(4)
C(9')-H(9')	0.9500
C(9')-C(8')	1.392(5)
C(2')-C(3')	1.565(5)
C(2')-C(1')	1.547(5)
C(7')-H(7')	0.9500
C(7')-C(6')	1.383(5)
C(7')-C(8')	1.383(5)
C(8')-H(8')	0.9500
C(2)-C(1)	1.565(5)
C(7)-H(7)	0.9500

C(2')-O(1')-H(1')	109.5
C(2)-O(1)-H(1)	108(3)
C(10)-O(2)-C(11)	116.1(3)
C(10')-O(2')-C(11')	115.5(3)
O(3)-C(10)-O(2)	124.1(3)
O(3)-C(10)-C(6)	124.1(3)
O(2)-C(10)-C(6)	111.7(3)
O(2')-C(11')-H(11A)	109.5
O(2')-C(11')-H(11B)	109.5
O(2')-C(11')-H(11C)	109.5
H(11A)-C(11')-H(11B)	109.5
H(11A)-C(11')-H(11C)	109.5
H(11B)-C(11')-H(11C)	109.5

O(2)-C(11)-H(11D)	109.5
O(2)-C(11)-H(11E)	109.5
O(2)-C(11)-H(11F)	109.5
H(11D)-C(11)-H(11E)	109.5
H(11D)-C(11)-H(11F)	109.5
H(11E)-C(11)-H(11F)	109.5
O(3')-C(10')-O(2')	123.6(3)
O(3')-C(10')-C(6')	124.2(3)
O(2')-C(10')-C(6')	112.1(3)
C(4')-C(5')-Br(1')	125.4(2)
C(6')-C(5')-Br(1')	114.4(2)
C(6')-C(5')-C(4')	120.2(3)
C(8)-C(9)-H(9)	119.0
C(8)-C(9)-C(4)	121.9(3)
C(4)-C(9)-H(9)	119.0
C(9)-C(8)-H(8)	120.0
C(7)-C(8)-C(9)	120.1(3)
C(7)-C(8)-H(8)	120.0
F(1)-C(3)-F(3)	107.9(3)
F(1)-C(3)-F(2)	107.9(3)
F(1)-C(3)-C(2)	109.7(3)
F(3)-C(3)-F(2)	107.6(3)
F(3)-C(3)-C(2)	112.0(3)
F(2)-C(3)-C(2)	111.6(3)
C(5')-C(4')-C(2')	123.6(3)
C(9')-C(4')-C(5')	117.3(3)
C(9')-C(4')-C(2')	119.0(3)
C(4)-C(5)-Br(1)	125.4(2)
C(6)-C(5)-Br(1)	114.6(2)
C(6)-C(5)-C(4)	120.0(3)
C(9)-C(4)-C(5)	117.4(3)
C(9)-C(4)-C(2)	118.9(3)
C(5)-C(4)-C(2)	123.5(3)
C(5)-C(6)-C(10)	121.0(3)
C(7)-C(6)-C(10)	117.8(3)
C(7)-C(6)-C(5)	121.2(3)

C(4')-C(9')-H(9')	118.7
C(8')-C(9')-C(4')	122.6(3)
C(8')-C(9')-H(9')	118.7
O(1')-C(2')-C(4')	115.0(3)
O(1')-C(2')-C(3')	105.8(3)
O(1')-C(2')-C(1')	105.6(3)
C(4')-C(2')-C(3')	113.0(3)
C(4')-C(2')-C(1')	108.1(3)
C(1')-C(2')-C(3')	108.9(3)
C(6')-C(7')-H(7')	120.0
C(6')-C(7')-C(8')	120.0(3)
C(8')-C(7')-H(7')	120.0
C(5')-C(6')-C(10')	121.9(3)
C(7')-C(6')-C(10')	117.4(3)
C(7')-C(6')-C(5')	120.7(3)
C(9')-C(8')-H(8')	120.4
C(7')-C(8')-C(9')	119.2(3)
C(7')-C(8')-H(8')	120.4
F(2')-C(3')-F(1')	107.9(3)
F(2')-C(3')-C(2')	114.9(3)
F(1')-C(3')-C(2')	109.5(3)
F(3')-C(3')-F(2')	106.8(3)
F(3')-C(3')-F(1')	106.7(3)
F(3')-C(3')-C(2')	110.6(3)
F(4')-C(1')-F(5')	107.4(3)
F(4')-C(1')-C(2')	111.1(3)
F(6')-C(1')-F(4')	108.1(3)
F(6')-C(1')-F(5')	107.7(3)
F(6')-C(1')-C(2')	110.2(3)
F(5')-C(1')-C(2')	112.2(3)
O(1)-C(2)-C(3)	105.1(3)
O(1)-C(2)-C(4)	115.3(3)
O(1)-C(2)-C(1)	105.2(2)
C(3)-C(2)-C(1)	109.9(3)
C(4)-C(2)-C(3)	108.4(2)
C(4)-C(2)-C(1)	112.6(3)

F(4)-C(1)-C(2)	113.8(3)
F(6)-C(1)-F(4)	107.9(3)
F(6)-C(1)-C(2)	110.3(3)
F(5)-C(1)-F(4)	106.1(3)
F(5)-C(1)-F(6)	107.4(3)
F(5)-C(1)-C(2)	110.9(3)
C(8)-C(7)-H(7)	120.3
C(6)-C(7)-C(8)	119.4(3)
C(6)-C(7)-H(7)	120.3

Symmetry transformations used to generate equivalent atoms:

Table 4. Anisotropic displacement parameters ($Å^2$x 10^3) for cain80_0m_a (**"HALF"**). The anisotropic displacement factor exponent takes the form: $-2\pi^2[h^2 a^{*2}U^{11} + ... + 2 h k a^* b^* U^{12}]$

	U^{11}	U^{22}	U^{33}	U^{23}	U^{13}	U^{12}
Br(1')	18(1)	13(1)	10(1)	1(1)	1(1)	-2(1)
Br(1)	17(1)	17(1)	9(1)	-1(1)	2(1)	-5(1)
F(1)	17(1)	17(1)	23(1)	3(1)	2(1)	5(1)
F(4')	22(1)	28(1)	20(1)	4(1)	3(1)	15(1)
F(2')	17(1)	30(1)	18(1)	-1(1)	-4(1)	-1(1)
F(4)	27(1)	23(1)	18(1)	-9(1)	4(1)	-7(1)
F(6')	22(1)	31(1)	17(1)	-5(1)	3(1)	14(1)
O(1')	12(1)	15(1)	16(1)	5(1)	4(1)	1(1)
F(6)	14(1)	22(1)	25(1)	-1(1)	-4(1)	2(1)
F(3)	28(1)	10(1)	34(1)	2(1)	11(1)	0(1)
O(1)	16(1)	13(1)	14(1)	-1(1)	6(1)	-1(1)
F(1')	23(1)	17(1)	26(1)	-9(1)	3(1)	1(1)
O(3)	18(1)	13(1)	28(1)	-6(1)	3(1)	2(1)
F(2)	30(1)	19(1)	23(1)	1(1)	14(1)	11(1)
O(3')	12(1)	18(1)	23(1)	-3(1)	2(1)	4(1)
F(3')	27(1)	28(1)	27(1)	3(1)	-1(1)	-17(1)
F(5)	22(1)	25(1)	25(1)	-2(1)	4(1)	-12(1)
O(2)	10(1)	14(1)	23(1)	-6(1)	-1(1)	2(1)
O(2')	13(1)	11(1)	21(1)	-6(1)	2(1)	1(1)
F(5')	15(1)	40(2)	45(1)	6(1)	16(1)	2(1)
C(10)	12(1)	12(2)	14(2)	-2(1)	5(1)	-2(1)
C(11')	16(2)	16(2)	23(2)	-7(1)	1(1)	-3(1)
C(11)	14(2)	21(2)	22(2)	-5(2)	-1(1)	-4(1)
C(10')	15(2)	11(2)	11(1)	1(1)	3(1)	1(1)
C(5')	14(2)	11(2)	7(1)	-1(1)	2(1)	3(1)
C(9)	13(2)	13(2)	13(2)	-1(1)	1(1)	3(1)
C(8)	15(2)	20(2)	9(2)	4(1)	2(1)	2(1)
C(3)	22(2)	12(2)	15(2)	1(1)	8(1)	2(1)
C(4')	10(1)	7(2)	14(2)	0(1)	4(1)	3(1)
C(5)	9(1)	11(2)	9(1)	1(1)	1(1)	5(1)
C(4)	9(1)	10(2)	13(2)	-1(1)	1(1)	2(1)

C(6)	7(1)	12(2)	16(2)	-2(1)	2(1)	3(1)
C(9')	16(2)	12(2)	14(2)	0(1)	3(1)	3(1)
C(2')	9(1)	12(2)	16(2)	1(1)	3(1)	-1(1)
C(7')	17(2)	12(2)	16(2)	0(1)	7(1)	-1(1)
C(6')	10(1)	9(2)	16(2)	-3(1)	3(1)	2(1)
C(8')	22(2)	14(2)	13(2)	-1(1)	6(1)	-1(1)
C(3')	18(2)	16(2)	17(2)	2(1)	0(1)	-4(1)
C(1')	14(2)	20(2)	18(2)	1(1)	3(1)	5(1)
C(2)	12(1)	10(2)	13(2)	-1(1)	3(1)	0(1)
C(1)	17(2)	16(2)	16(2)	-2(1)	4(1)	-1(1)
C(7)	12(2)	14(2)	16(2)	2(1)	4(1)	1(1)

Table 5. Hydrogen coordinates (x 10^4) and isotropic displacement parameters ($Å^2$x 10^3) for cain80_0m_a (**"HALF"**).

	x	y	z	U(eq)
H(1')	4705	4050	5694	21
H(11A)	7985	8249	6617	28
H(11B)	7775	7598	7552	28
H(11C)	7243	8582	7348	28
H(11D)	7844	3594	6436	29
H(11E)	8302	2690	7055	29
H(11F)	7347	3161	7346	29
H(9)	4301	465	2865	16
H(8)	5132	1713	2317	18
H(9')	4008	5563	3059	17
H(7')	6381	7209	3761	18
H(8')	5078	6622	2527	19
H(7)	5953	2764	3552	17
H(1)	3450(30)	460(30)	5680(30)	12(11)

X-RAY OF 2

Table 1. Crystal data and structure refinement for cain84_0m_a (**2**).

Identification code	B31Cs	
Empirical formula	C14 H9 Br F12 O2	
Formula weight	517.12	
Temperature	100.0 K	
Wavelength	0.71073 Å	
Crystal system	Monoclinic	
Space group	P 21/n	
Unit cell dimensions	a = 11.9324(10) Å	α= 90°.
	b = 12.4694(12) Å	β= 116.905(4)°.
	c = 12.4948(10) Å	γ = 90°.
Volume	1657.9(3) $Å^3$	
Z	4	
Density (calculated)	2.072 Mg/m^3	
Absorption coefficient	2.618 mm^{-1}	
F(000)	1008	
Crystal size	0.33 x 0.32 x 0.25 mm^3	
Theta range for data collection	1.959 to 26.386°.	
Index ranges	-14<=h<=14, -13<=k<=15, -15<=l<=11	
Reflections collected	8685	
Independent reflections	3370 [R(int) = 0.0365]	
Completeness to theta = 25.242°	99.7 %	
Absorption correction	Semi-empirical from equivalents	
Max. and min. transmission	0.7454 and 0.6400	
Refinement method	Full-matrix least-squares on F^2	
Data / restraints / parameters	3370 / 0 / 264	
Goodness-of-fit on F^2	1.075	
Final R indices [I>2sigma(I)]	R1 = 0.0464, wR2 = 0.1265	
R indices (all data)	R1 = 0.0573, wR2 = 0.1341	
Extinction coefficient	n/a	
Largest diff. peak and hole	1.690 and -0.525 e.$Å^{-3}$	

Table 2. Atomic coordinates (x 10^4) and equivalent isotropic displacement parameters ($Å^2$x 10^3) for cain84_0m_a (**2**). U(eq) is defined as one third of the trace of the orthogonalized U^{ij} tensor.

	x	y	z	U(eq)
Br(1)	5171(1)	2633(1)	2871(1)	17(1)
F(12)	8013(2)	3404(2)	4264(2)	24(1)
F(11)	9253(2)	3791(2)	3493(2)	25(1)
F(8)	8153(2)	5668(2)	1954(3)	27(1)
F(7)	6437(2)	5126(2)	449(2)	22(1)
F(10)	8458(2)	5052(2)	4111(2)	25(1)
F(9)	8120(2)	4180(2)	1070(2)	26(1)
O(2)	6912(3)	3082(2)	1916(2)	14(1)
F(5)	1598(3)	5632(2)	2774(3)	36(1)
F(1)	4091(3)	3270(2)	4573(2)	32(1)
O(1)	2554(3)	3103(3)	2224(3)	21(1)
F(3)	3582(3)	4911(2)	4645(3)	33(1)
F(6)	636(3)	4208(3)	1895(3)	39(1)
F(4)	1446(3)	5233(2)	1045(3)	36(1)
F(2)	2202(3)	3658(3)	4177(3)	37(1)
C(1)	5002(3)	4135(3)	2566(3)	10(1)
C(6)	5938(3)	4689(3)	2399(3)	9(1)
C(4)	4934(4)	6364(3)	2454(4)	15(1)
C(10)	2187(4)	2988(4)	949(4)	20(1)
C(5)	5899(4)	5808(3)	2377(4)	13(1)
C(2)	3971(4)	4685(3)	2578(3)	11(1)
C(12)	8208(4)	4099(3)	3547(4)	17(1)
C(3)	3967(4)	5803(3)	2528(4)	13(1)
C(13)	7449(4)	4792(3)	1437(4)	18(1)
C(11)	7061(3)	4152(3)	2295(3)	10(1)
C(14)	5956(4)	2851(4)	723(4)	21(1)
C(7)	2861(4)	4132(3)	2683(4)	13(1)
C(8)	1632(4)	4819(4)	2112(5)	27(1)
C(9)	3192(4)	3993(4)	4042(4)	25(1)

Table 3. Bond lengths [Å] and angles [°] for cain84_0m_a (**2**).

Br(1)-C(1)	1.905(4)
F(12)-C(12)	1.340(5)
F(11)-C(12)	1.336(5)
F(8)-C(13)	1.351(5)
F(7)-C(13)	1.344(5)
F(10)-C(12)	1.345(5)
F(9)-C(13)	1.328(5)
O(2)-C(11)	1.400(5)
O(2)-C(14)	1.438(5)
F(5)-C(8)	1.320(6)
F(1)-C(9)	1.326(6)
O(1)-C(10)	1.455(5)
O(1)-C(7)	1.386(5)
F(3)-C(9)	1.333(5)
F(6)-C(8)	1.332(5)
F(4)-C(8)	1.351(6)
F(2)-C(9)	1.334(5)
C(1)-C(6)	1.408(5)
C(1)-C(2)	1.414(5)
C(6)-C(5)	1.396(5)
C(6)-C(11)	1.554(5)
C(4)-H(4)	0.9500
C(4)-C(5)	1.384(6)
C(4)-C(3)	1.388(6)
C(10)-H(10A)	0.9800
C(10)-H(10B)	0.9800
C(10)-H(10C)	0.9800
C(5)-H(5)	0.9500
C(2)-C(3)	1.395(5)
C(2)-C(7)	1.552(5)
C(12)-C(11)	1.545(5)
C(3)-H(3)	0.9500
C(13)-C(11)	1.566(5)
C(14)-H(14A)	0.9800

C(14)-H(14B)	0.9800
C(14)-H(14C)	0.9800
C(7)-C(8)	1.564(6)
C(7)-C(9)	1.569(6)

C(11)-O(2)-C(14)	117.5(3)
C(7)-O(1)-C(10)	115.9(3)
C(6)-C(1)-Br(1)	119.5(3)
C(6)-C(1)-C(2)	121.1(4)
C(2)-C(1)-Br(1)	119.3(3)
C(1)-C(6)-C(11)	124.9(3)
C(5)-C(6)-C(1)	118.1(3)
C(5)-C(6)-C(11)	116.8(3)
C(5)-C(4)-H(4)	120.1
C(5)-C(4)-C(3)	119.7(4)
C(3)-C(4)-H(4)	120.1
O(1)-C(10)-H(10A)	109.5
O(1)-C(10)-H(10B)	109.5
O(1)-C(10)-H(10C)	109.5
H(10A)-C(10)-H(10B)	109.5
H(10A)-C(10)-H(10C)	109.5
H(10B)-C(10)-H(10C)	109.5
C(6)-C(5)-H(5)	119.3
C(4)-C(5)-C(6)	121.4(4)
C(4)-C(5)-H(5)	119.3
C(1)-C(2)-C(7)	124.4(3)
C(3)-C(2)-C(1)	118.0(4)
C(3)-C(2)-C(7)	117.5(3)
F(12)-C(12)-F(10)	106.6(3)
F(12)-C(12)-C(11)	111.3(3)
F(11)-C(12)-F(12)	107.3(3)
F(11)-C(12)-F(10)	106.8(3)
F(11)-C(12)-C(11)	112.4(3)
F(10)-C(12)-C(11)	112.2(3)
C(4)-C(3)-C(2)	121.3(4)
C(4)-C(3)-H(3)	119.3

C(2)-C(3)-H(3)	119.3
F(8)-C(13)-C(11)	113.4(3)
F(7)-C(13)-F(8)	107.1(3)
F(7)-C(13)-C(11)	111.4(3)
F(9)-C(13)-F(8)	106.7(3)
F(9)-C(13)-F(7)	107.0(3)
F(9)-C(13)-C(11)	110.8(3)
O(2)-C(11)-C(6)	117.2(3)
O(2)-C(11)-C(12)	102.7(3)
O(2)-C(11)-C(13)	106.5(3)
C(6)-C(11)-C(13)	111.8(3)
C(12)-C(11)-C(6)	109.7(3)
C(12)-C(11)-C(13)	108.3(3)
O(2)-C(14)-H(14A)	109.5
O(2)-C(14)-H(14B)	109.5
O(2)-C(14)-H(14C)	109.5
H(14A)-C(14)-H(14B)	109.5
H(14A)-C(14)-H(14C)	109.5
H(14B)-C(14)-H(14C)	109.5
O(1)-C(7)-C(2)	116.9(3)
O(1)-C(7)-C(8)	107.5(3)
O(1)-C(7)-C(9)	103.7(3)
C(2)-C(7)-C(8)	112.2(3)
C(2)-C(7)-C(9)	109.5(3)
C(8)-C(7)-C(9)	106.3(3)
F(5)-C(8)-F(6)	106.8(4)
F(5)-C(8)-F(4)	106.7(4)
F(5)-C(8)-C(7)	115.0(4)
F(6)-C(8)-F(4)	106.5(4)
F(6)-C(8)-C(7)	110.2(4)
F(4)-C(8)-C(7)	111.2(4)
F(1)-C(9)-F(3)	107.8(4)
F(1)-C(9)-F(2)	106.6(4)
F(1)-C(9)-C(7)	111.5(4)
F(3)-C(9)-F(2)	107.5(4)
F(3)-C(9)-C(7)	111.6(4)

F(2)-C(9)-C(7)	111.5(4)

Symmetry transformations used to generate equivalent atoms:

Table 4. Anisotropic displacement parameters ($Å^2 x 10^3$) for cain84_0m_a (**2**). The anisotropic displacement factor exponent takes the form: $-2\pi^2[h^2 a^{*2}U^{11} + ... + 2 h k a^* b^* U^{12}]$

	U^{11}	U^{22}	U^{33}	U^{23}	U^{13}	U^{12}
Br(1)	16(1)	9(1)	29(1)	5(1)	13(1)	2(1)
F(12)	20(1)	31(2)	15(1)	8(1)	3(1)	0(1)
F(11)	10(1)	39(2)	23(1)	0(1)	5(1)	4(1)
F(8)	22(1)	24(1)	40(2)	1(1)	18(1)	-10(1)
F(7)	24(1)	28(1)	16(1)	8(1)	11(1)	5(1)
F(10)	18(1)	25(1)	22(1)	-9(1)	0(1)	-4(1)
F(9)	22(1)	36(2)	30(2)	5(1)	20(1)	6(1)
O(2)	14(1)	13(1)	12(1)	-1(1)	3(1)	1(1)
F(5)	31(2)	24(2)	66(2)	-2(1)	32(2)	8(1)
F(1)	44(2)	36(2)	16(1)	9(1)	12(1)	11(1)
O(1)	22(2)	21(2)	20(2)	-4(1)	9(1)	-5(1)
F(3)	47(2)	32(2)	28(2)	-14(1)	25(2)	-14(1)
F(6)	18(1)	41(2)	56(2)	1(2)	16(1)	-1(1)
F(4)	23(2)	39(2)	36(2)	15(1)	5(1)	5(1)
F(2)	43(2)	45(2)	38(2)	-6(2)	31(2)	-15(2)
C(1)	9(2)	9(2)	7(2)	-1(1)	1(2)	0(1)
C(6)	9(2)	9(2)	6(2)	0(1)	1(2)	0(1)
C(4)	24(2)	8(2)	15(2)	1(2)	10(2)	1(2)
C(10)	27(2)	20(2)	9(2)	-4(2)	5(2)	-6(2)
C(5)	17(2)	13(2)	11(2)	1(2)	7(2)	-3(2)
C(2)	10(2)	14(2)	7(2)	-1(2)	4(2)	0(2)
C(12)	12(2)	19(2)	18(2)	-2(2)	5(2)	-1(2)
C(3)	18(2)	9(2)	13(2)	0(2)	8(2)	6(2)
C(13)	17(2)	19(2)	20(2)	1(2)	11(2)	-1(2)
C(11)	8(2)	12(2)	8(2)	2(2)	3(2)	1(1)
C(14)	18(2)	21(2)	15(2)	-7(2)	1(2)	1(2)
C(7)	10(2)	12(2)	18(2)	0(2)	8(2)	1(2)
C(8)	19(2)	27(3)	35(3)	3(2)	13(2)	1(2)
C(9)	25(2)	30(2)	24(2)	-3(2)	14(2)	-3(2)

Table 5. Hydrogen coordinates (x 10^4) and isotropic displacement parameters ($Å^2$x 10^3) for cain84_0m_a (**2**).

	x	y	z	U(eq)
H(4)	4934	7126	2456	18
H(10A)	1349	3296	482	30
H(10B)	2172	2225	752	30
H(10C)	2793	3364	755	30
H(5)	6548	6196	2307	16
H(3)	3288	6188	2544	16
H(14A)	6270	3022	142	31
H(14B)	5208	3284	552	31
H(14C)	5737	2088	664	31

X-RAY OF 3

Table 1. Crystal data and structure refinement for Cain58 (**3**).

Identification code	JH167	
Empirical formula	C78 H87 O6 P3	
Formula weight	1213.38	
Temperature	100.0 K	
Wavelength	0.71073 Å	
Crystal system	Triclinic	
Space group	P-1	
Unit cell dimensions	a = 10.515(5) Å	α= 100.80(3)°.
	b = 15.037(8) Å	β= 94.78(2)°.
	c = 22.719(11) Å	γ = 103.351(19)°.
Volume	3403(3) Å^3	
Z	2	
Density (calculated)	1.184 Mg/m^3	
Absorption coefficient	0.140 mm^{-1}	
F(000)	1296	
Crystal size	0.28 x 0.08 x 0.04 mm^3	
Theta range for data collection	1.425 to 23.409°.	
Index ranges	-11<=h<=11, -16<=k<=16, -25<=l<=25	
Reflections collected	45744	
Independent reflections	9821 [R(int) = 0.0824]	
Completeness to theta = 23.409°	99.0 %	
Absorption correction	Semi-empirical from equivalents	
Max. and min. transmission	0.2571 and 0.2038	
Refinement method	Full-matrix least-squares on F^2	
Data / restraints / parameters	9821 / 0 / 803	
Goodness-of-fit on F^2	1.096	
Final R indices [I>2sigma(I)]	R1 = 0.0788, wR2 = 0.1660	
R indices (all data)	R1 = 0.1154, wR2 = 0.1799	
Extinction coefficient	n/a	
Largest diff. peak and hole	0.408 and -0.375 e.Å^{-3}	

Table 2. Atomic coordinates (x 10^4) and equivalent isotropic displacement parameters (Å^{2}x 10^3) for Cain58 (**3**). U(eq) is defined as one third of the trace of the orthogonalized U^{ij} tensor.

	x	y	z	U(eq)
P(2)	2753(1)	7331(1)	7046(1)	23(1)
P(3)	1107(1)	6059(1)	6916(1)	23(1)
P(1)	2046(1)	6854(1)	7843(1)	23(1)
O(6)	-511(3)	3706(2)	7037(2)	24(1)
O(2)	5561(3)	7449(2)	7824(2)	25(1)
O(1)	898(3)	5570(2)	9336(2)	27(1)
O(4)	1390(3)	7449(3)	5688(2)	33(1)
O(3)	4426(3)	10065(2)	8127(2)	34(1)
O(5)	3542(3)	5448(3)	5367(2)	32(1)
C(6)	2497(5)	5803(4)	8649(2)	24(1)
C(75)	-2555(5)	3940(4)	7362(2)	24(1)
C(58)	1750(5)	4409(4)	7056(2)	23(1)
C(57)	2456(5)	3728(4)	6975(2)	25(1)
C(17)	5027(5)	6476(4)	7585(2)	24(1)
C(3)	4848(5)	5459(4)	8333(2)	26(1)
C(28)	942(5)	8331(4)	6632(2)	29(1)
C(1)	3045(4)	6163(3)	8166(2)	19(1)
C(2)	4265(5)	6012(3)	8025(2)	22(1)
C(53)	2001(4)	5136(3)	6746(2)	20(1)
C(18)	6811(4)	7648(3)	8159(2)	21(1)
C(27)	1925(4)	8299(4)	7089(2)	22(1)
C(7)	1280(5)	6038(4)	8869(2)	27(1)
C(60)	3811(5)	6111(4)	5010(2)	26(1)
C(23)	7901(5)	7646(3)	7849(2)	22(1)
C(55)	3564(5)	4428(4)	6238(2)	28(1)
C(59)	3093(5)	5827(4)	5915(2)	30(1)
C(39)	6619(5)	9907(4)	8017(2)	28(1)
C(20)	8191(5)	8114(3)	9097(2)	26(1)
C(19)	6942(5)	7892(3)	8783(2)	26(1)
C(70)	-1604(5)	3427(4)	7332(2)	25(1)
C(65)	5060(5)	6697(4)	5086(2)	29(1)

C(22)	9142(5)	7891(4)	8187(3)	30(1)
C(32)	2440(5)	9083(4)	7557(2)	28(1)
C(54)	2880(5)	5118(4)	6306(2)	25(1)
C(35)	6034(5)	10509(4)	8999(2)	32(1)
C(74)	-3683(5)	3586(4)	7614(2)	29(1)
C(72)	-2897(5)	2291(4)	7796(2)	32(1)
C(76)	-2388(5)	4816(4)	7134(2)	30(1)
C(44)	1583(5)	8097(4)	5317(2)	31(1)
C(71)	-1767(5)	2599(4)	7538(2)	29(1)
C(34)	5710(5)	10146(3)	8389(2)	27(1)
C(38)	7912(5)	10052(3)	8288(3)	31(1)
C(69)	643(5)	4260(4)	7437(2)	25(1)
C(33)	3593(5)	9139(4)	8021(2)	29(1)
C(8)	154(5)	6008(4)	9724(2)	29(1)
C(21)	9309(5)	8120(4)	8811(2)	30(1)
C(5)	3102(5)	5243(4)	8938(2)	31(1)
C(73)	-3870(5)	2778(4)	7835(2)	28(1)
C(56)	3381(5)	3748(4)	6576(2)	31(1)
C(61)	2816(5)	6148(4)	4585(2)	34(1)
C(9)	-1204(5)	5853(4)	9544(3)	34(1)
C(49)	2738(5)	8801(4)	5427(2)	32(1)
C(24)	5755(5)	7929(4)	9116(2)	34(1)
C(64)	5312(5)	7364(4)	4730(2)	33(1)
C(4)	4280(5)	5081(4)	8778(3)	33(1)
C(31)	1936(5)	9864(4)	7601(3)	38(2)
C(37)	8271(5)	10414(4)	8905(3)	34(1)
C(45)	634(5)	7981(5)	4822(3)	41(2)
C(26)	7757(5)	7437(4)	7167(2)	34(1)
C(63)	4327(6)	7431(4)	4305(2)	35(1)
C(13)	788(6)	6547(4)	10271(2)	33(1)
C(62)	3089(6)	6820(4)	4239(2)	38(2)
C(36)	7336(6)	10647(4)	9252(3)	35(1)
C(43)	410(5)	7563(4)	6086(2)	34(1)
C(29)	479(5)	9137(4)	6693(3)	43(2)
C(48)	2907(5)	9418(5)	5046(3)	42(2)
C(50)	3800(5)	8880(4)	5938(2)	36(1)

C(10)	-1902(6)	6281(5)	9961(3)	48(2)
C(40)	6242(6)	9554(4)	7349(2)	39(2)
C(25)	10672(5)	8394(4)	9172(3)	38(2)
C(66)	6150(5)	6626(5)	5536(3)	42(2)
C(78)	-760(5)	2026(4)	7466(3)	39(2)
C(16)	2250(6)	6728(4)	10431(3)	43(2)
C(77)	-5102(5)	2428(4)	8110(3)	43(2)
C(11)	-1315(7)	6814(4)	10516(3)	44(2)
C(68)	1465(6)	5471(5)	4502(3)	51(2)
C(41)	9665(6)	10547(4)	9186(3)	47(2)
C(67)	4589(7)	8155(4)	3920(3)	48(2)
C(30)	944(6)	9881(5)	7166(3)	47(2)
C(12)	39(7)	6950(4)	10670(3)	47(2)
C(47)	1982(6)	9362(5)	4566(3)	55(2)
C(14)	-1846(5)	5290(5)	8933(3)	49(2)
C(42)	5015(6)	10786(5)	9380(3)	52(2)
C(46)	867(6)	8643(5)	4462(3)	54(2)
C(52)	-590(6)	7177(5)	4662(3)	54(2)
C(15)	-2133(8)	7242(5)	10962(4)	74(2)
C(51)	2196(7)	10080(7)	4175(4)	107(4)

Table 3. Bond lengths [Å] and angles [°] for Cain58 (**3**).

P(2)-P(3)	2.217(2)
P(2)-P(1)	2.194(2)
P(2)-C(27)	1.853(5)
P(3)-P(1)	2.237(2)
P(3)-C(53)	1.850(5)
P(1)-C(1)	1.839(5)
O(6)-C(70)	1.399(6)
O(6)-C(69)	1.434(6)
O(2)-C(17)	1.422(6)
O(2)-C(18)	1.400(6)
O(1)-C(7)	1.415(6)
O(1)-C(8)	1.400(6)
O(4)-C(44)	1.395(6)
O(4)-C(43)	1.445(6)
O(3)-C(34)	1.398(6)
O(3)-C(33)	1.428(6)
O(5)-C(60)	1.393(6)
O(5)-C(59)	1.432(6)
C(6)-C(1)	1.420(7)
C(6)-C(7)	1.504(7)
C(6)-C(5)	1.388(7)
C(75)-C(70)	1.395(7)
C(75)-C(74)	1.398(7)
C(75)-C(76)	1.483(7)
C(58)-C(57)	1.392(7)
C(58)-C(53)	1.396(7)
C(58)-C(69)	1.508(7)
C(57)-H(57)	0.9500
C(57)-C(56)	1.384(7)
C(17)-H(17A)	0.9900
C(17)-H(17B)	0.9900
C(17)-C(2)	1.505(7)
C(3)-H(3)	0.9500
C(3)-C(2)	1.391(7)

C(3)-C(4)	1.365(7)
C(28)-C(27)	1.418(7)
C(28)-C(43)	1.494(8)
C(28)-C(29)	1.395(8)
C(1)-C(2)	1.406(7)
C(53)-C(54)	1.417(7)
C(18)-C(23)	1.395(7)
C(18)-C(19)	1.385(7)
C(27)-C(32)	1.398(7)
C(7)-H(7A)	0.9900
C(7)-H(7B)	0.9900
C(60)-C(65)	1.379(7)
C(60)-C(61)	1.382(7)
C(23)-C(22)	1.391(7)
C(23)-C(26)	1.509(7)
C(55)-H(55)	0.9500
C(55)-C(54)	1.386(7)
C(55)-C(56)	1.377(7)
C(59)-H(59A)	0.9900
C(59)-H(59B)	0.9900
C(59)-C(54)	1.502(7)
C(39)-C(34)	1.391(7)
C(39)-C(38)	1.397(7)
C(39)-C(40)	1.496(7)
C(20)-H(20)	0.9500
C(20)-C(19)	1.378(7)
C(20)-C(21)	1.389(7)
C(19)-C(24)	1.518(7)
C(70)-C(71)	1.390(7)
C(65)-C(64)	1.397(7)
C(65)-C(66)	1.507(7)
C(22)-H(22)	0.9500
C(22)-C(21)	1.380(7)
C(32)-C(33)	1.515(7)
C(32)-C(31)	1.387(7)
C(35)-C(34)	1.374(7)

C(35)-C(36)	1.394(7)
C(35)-C(42)	1.515(8)
C(74)-H(74)	0.9500
C(74)-C(73)	1.379(7)
C(72)-H(72)	0.9500
C(72)-C(71)	1.390(7)
C(72)-C(73)	1.389(7)
C(76)-H(76A)	0.9800
C(76)-H(76B)	0.9800
C(76)-H(76C)	0.9800
C(44)-C(49)	1.384(7)
C(44)-C(45)	1.397(7)
C(71)-C(78)	1.513(7)
C(38)-H(38)	0.9500
C(38)-C(37)	1.389(8)
C(69)-H(69A)	0.9900
C(69)-H(69B)	0.9900
C(33)-H(33A)	0.9900
C(33)-H(33B)	0.9900
C(8)-C(9)	1.404(7)
C(8)-C(13)	1.372(8)
C(21)-C(25)	1.517(7)
C(5)-H(5)	0.9500
C(5)-C(4)	1.380(7)
C(73)-C(77)	1.513(7)
C(56)-H(56)	0.9500
C(61)-C(62)	1.388(8)
C(61)-C(68)	1.519(8)
C(9)-C(10)	1.399(8)
C(9)-C(14)	1.496(8)
C(49)-C(48)	1.375(8)
C(49)-C(50)	1.509(7)
C(24)-H(24A)	0.9800
C(24)-H(24B)	0.9800
C(24)-H(24C)	0.9800
C(64)-H(64)	0.9500

C(64)-C(63)	1.390(8)
C(4)-H(4)	0.9500
C(31)-H(31)	0.9500
C(31)-C(30)	1.384(8)
C(37)-C(36)	1.376(8)
C(37)-C(41)	1.503(8)
C(45)-C(46)	1.395(8)
C(45)-C(52)	1.512(8)
C(26)-H(26A)	0.9800
C(26)-H(26B)	0.9800
C(26)-H(26C)	0.9800
C(63)-C(62)	1.387(8)
C(63)-C(67)	1.511(8)
C(13)-C(16)	1.499(8)
C(13)-C(12)	1.394(8)
C(62)-H(62)	0.9500
C(36)-H(36)	0.9500
C(43)-H(43A)	0.9900
C(43)-H(43B)	0.9900
C(29)-H(29)	0.9500
C(29)-C(30)	1.361(9)
C(48)-H(48)	0.9500
C(48)-C(47)	1.376(8)
C(50)-H(50A)	0.9800
C(50)-H(50B)	0.9800
C(50)-H(50C)	0.9800
C(10)-H(10)	0.9500
C(10)-C(11)	1.371(9)
C(40)-H(40A)	0.9800
C(40)-H(40B)	0.9800
C(40)-H(40C)	0.9800
C(25)-H(25A)	0.9800
C(25)-H(25B)	0.9800
C(25)-H(25C)	0.9800
C(66)-H(66A)	0.9800
C(66)-H(66B)	0.9800

C(66)-H(66C)	0.9800
C(78)-H(78A)	0.9800
C(78)-H(78B)	0.9800
C(78)-H(78C)	0.9800
C(16)-H(16A)	0.9800
C(16)-H(16B)	0.9800
C(16)-H(16C)	0.9800
C(77)-H(77A)	0.9800
C(77)-H(77B)	0.9800
C(77)-H(77C)	0.9800
C(11)-C(12)	1.396(9)
C(11)-C(15)	1.527(8)
C(68)-H(68A)	0.9800
C(68)-H(68B)	0.9800
C(68)-H(68C)	0.9800
C(41)-H(41A)	0.9800
C(41)-H(41B)	0.9800
C(41)-H(41C)	0.9800
C(67)-H(67A)	0.9800
C(67)-H(67B)	0.9800
C(67)-H(67C)	0.9800
C(30)-H(30)	0.9500
C(12)-H(12)	0.9500
C(47)-C(46)	1.368(9)
C(47)-C(51)	1.513(9)
C(14)-H(14A)	0.9800
C(14)-H(14B)	0.9800
C(14)-H(14C)	0.9800
C(42)-H(42A)	0.9800
C(42)-H(42B)	0.9800
C(42)-H(42C)	0.9800
C(46)-H(46)	0.9500
C(52)-H(52A)	0.9800
C(52)-H(52B)	0.9800
C(52)-H(52C)	0.9800
C(15)-H(15A)	0.9800

C(15)-H(15B)	0.9800
C(15)-H(15C)	0.9800
C(51)-H(51A)	0.9800
C(51)-H(51B)	0.9800
C(51)-H(51C)	0.9800
P(1)-P(2)-P(3)	60.95(7)
C(27)-P(2)-P(3)	104.08(16)
C(27)-P(2)-P(1)	96.46(16)
P(2)-P(3)-P(1)	59.02(7)
C(53)-P(3)-P(2)	100.54(17)
C(53)-P(3)-P(1)	103.76(16)
P(2)-P(1)-P(3)	60.03(7)
C(1)-P(1)-P(2)	113.78(16)
C(1)-P(1)-P(3)	108.26(17)
C(70)-O(6)-C(69)	113.9(4)
C(18)-O(2)-C(17)	112.8(4)
C(8)-O(1)-C(7)	113.9(4)
C(44)-O(4)-C(43)	113.6(4)
C(34)-O(3)-C(33)	113.8(4)
C(60)-O(5)-C(59)	110.9(4)
C(1)-C(6)-C(7)	121.0(4)
C(5)-C(6)-C(1)	120.1(5)
C(5)-C(6)-C(7)	118.9(5)
C(70)-C(75)-C(74)	116.9(5)
C(70)-C(75)-C(76)	121.9(4)
C(74)-C(75)-C(76)	121.2(5)
C(57)-C(58)-C(53)	120.0(5)
C(57)-C(58)-C(69)	116.7(5)
C(53)-C(58)-C(69)	123.1(5)
C(58)-C(57)-H(57)	119.7
C(56)-C(57)-C(58)	120.6(5)
C(56)-C(57)-H(57)	119.7
O(2)-C(17)-H(17A)	109.4
O(2)-C(17)-H(17B)	109.4
O(2)-C(17)-C(2)	111.3(4)

H(17A)-C(17)-H(17B)	108.0
C(2)-C(17)-H(17A)	109.4
C(2)-C(17)-H(17B)	109.4
C(2)-C(3)-H(3)	119.2
C(4)-C(3)-H(3)	119.2
C(4)-C(3)-C(2)	121.6(5)
C(27)-C(28)-C(43)	123.6(5)
C(29)-C(28)-C(27)	117.6(5)
C(29)-C(28)-C(43)	118.8(5)
C(6)-C(1)-P(1)	112.1(3)
C(2)-C(1)-P(1)	129.1(4)
C(2)-C(1)-C(6)	118.6(4)
C(3)-C(2)-C(17)	117.8(4)
C(3)-C(2)-C(1)	119.1(5)
C(1)-C(2)-C(17)	123.0(4)
C(58)-C(53)-P(3)	117.4(4)
C(58)-C(53)-C(54)	119.1(5)
C(54)-C(53)-P(3)	123.4(4)
C(23)-C(18)-O(2)	118.7(4)
C(19)-C(18)-O(2)	119.4(4)
C(19)-C(18)-C(23)	121.8(4)
C(28)-C(27)-P(2)	123.2(4)
C(32)-C(27)-P(2)	116.8(4)
C(32)-C(27)-C(28)	119.4(5)
O(1)-C(7)-C(6)	110.4(4)
O(1)-C(7)-H(7A)	109.6
O(1)-C(7)-H(7B)	109.6
C(6)-C(7)-H(7A)	109.6
C(6)-C(7)-H(7B)	109.6
H(7A)-C(7)-H(7B)	108.1
C(65)-C(60)-O(5)	119.2(5)
C(65)-C(60)-C(61)	122.3(5)
C(61)-C(60)-O(5)	118.5(5)
C(18)-C(23)-C(26)	121.4(4)
C(22)-C(23)-C(18)	117.9(5)
C(22)-C(23)-C(26)	120.6(4)

C(54)-C(55)-H(55)	119.2
C(56)-C(55)-H(55)	119.2
C(56)-C(55)-C(54)	121.6(5)
O(5)-C(59)-H(59A)	109.7
O(5)-C(59)-H(59B)	109.7
O(5)-C(59)-C(54)	109.8(4)
H(59A)-C(59)-H(59B)	108.2
C(54)-C(59)-H(59A)	109.7
C(54)-C(59)-H(59B)	109.7
C(34)-C(39)-C(38)	117.2(5)
C(34)-C(39)-C(40)	121.4(5)
C(38)-C(39)-C(40)	121.4(5)
C(19)-C(20)-H(20)	118.7
C(19)-C(20)-C(21)	122.6(5)
C(21)-C(20)-H(20)	118.7
C(18)-C(19)-C(24)	121.5(5)
C(20)-C(19)-C(18)	117.9(5)
C(20)-C(19)-C(24)	120.6(5)
C(75)-C(70)-O(6)	119.1(4)
C(71)-C(70)-O(6)	118.7(4)
C(71)-C(70)-C(75)	121.9(5)
C(60)-C(65)-C(64)	118.5(5)
C(60)-C(65)-C(66)	121.7(5)
C(64)-C(65)-C(66)	119.7(5)
C(23)-C(22)-H(22)	119.0
C(21)-C(22)-C(23)	122.0(5)
C(21)-C(22)-H(22)	119.0
C(27)-C(32)-C(33)	122.3(5)
C(31)-C(32)-C(27)	120.6(5)
C(31)-C(32)-C(33)	117.0(5)
C(53)-C(54)-C(59)	121.8(5)
C(55)-C(54)-C(53)	119.0(5)
C(55)-C(54)-C(59)	119.3(4)
C(34)-C(35)-C(36)	118.0(5)
C(34)-C(35)-C(42)	120.8(5)
C(36)-C(35)-C(42)	121.1(5)

C(75)-C(74)-H(74)	118.5
C(73)-C(74)-C(75)	123.0(5)
C(73)-C(74)-H(74)	118.5
C(71)-C(72)-H(72)	119.3
C(73)-C(72)-H(72)	119.3
C(73)-C(72)-C(71)	121.4(5)
C(75)-C(76)-H(76A)	109.5
C(75)-C(76)-H(76B)	109.5
C(75)-C(76)-H(76C)	109.5
H(76A)-C(76)-H(76B)	109.5
H(76A)-C(76)-H(76C)	109.5
H(76B)-C(76)-H(76C)	109.5
O(4)-C(44)-C(45)	118.7(5)
C(49)-C(44)-O(4)	119.3(4)
C(49)-C(44)-C(45)	121.9(5)
C(70)-C(71)-C(78)	120.9(5)
C(72)-C(71)-C(70)	118.7(5)
C(72)-C(71)-C(78)	120.4(5)
C(39)-C(34)-O(3)	118.8(5)
C(35)-C(34)-O(3)	118.1(5)
C(35)-C(34)-C(39)	123.0(5)
C(39)-C(38)-H(38)	119.4
C(37)-C(38)-C(39)	121.3(5)
C(37)-C(38)-H(38)	119.4
O(6)-C(69)-C(58)	106.8(4)
O(6)-C(69)-H(69A)	110.4
O(6)-C(69)-H(69B)	110.4
C(58)-C(69)-H(69A)	110.4
C(58)-C(69)-H(69B)	110.4
H(69A)-C(69)-H(69B)	108.6
O(3)-C(33)-C(32)	107.3(4)
O(3)-C(33)-H(33A)	110.2
O(3)-C(33)-H(33B)	110.2
C(32)-C(33)-H(33A)	110.2
C(32)-C(33)-H(33B)	110.2
H(33A)-C(33)-H(33B)	108.5

O(1)-C(8)-C(9)	118.7(5)
C(13)-C(8)-O(1)	117.9(5)
C(13)-C(8)-C(9)	123.4(5)
C(20)-C(21)-C(25)	121.0(5)
C(22)-C(21)-C(20)	117.8(5)
C(22)-C(21)-C(25)	121.1(5)
C(6)-C(5)-H(5)	120.0
C(4)-C(5)-C(6)	120.0(5)
C(4)-C(5)-H(5)	120.0
C(74)-C(73)-C(72)	118.2(5)
C(74)-C(73)-C(77)	121.1(5)
C(72)-C(73)-C(77)	120.8(5)
C(57)-C(56)-H(56)	120.3
C(55)-C(56)-C(57)	119.5(5)
C(55)-C(56)-H(56)	120.3
C(60)-C(61)-C(62)	118.1(5)
C(60)-C(61)-C(68)	120.8(5)
C(62)-C(61)-C(68)	121.2(5)
C(8)-C(9)-C(14)	121.5(5)
C(10)-C(9)-C(8)	116.0(6)
C(10)-C(9)-C(14)	122.4(5)
C(44)-C(49)-C(50)	121.5(5)
C(48)-C(49)-C(44)	117.9(5)
C(48)-C(49)-C(50)	120.7(5)
C(19)-C(24)-H(24A)	109.5
C(19)-C(24)-H(24B)	109.5
C(19)-C(24)-H(24C)	109.5
H(24A)-C(24)-H(24B)	109.5
H(24A)-C(24)-H(24C)	109.5
H(24B)-C(24)-H(24C)	109.5
C(65)-C(64)-H(64)	119.7
C(63)-C(64)-C(65)	120.7(5)
C(63)-C(64)-H(64)	119.7
C(3)-C(4)-C(5)	120.4(5)
C(3)-C(4)-H(4)	119.8
C(5)-C(4)-H(4)	119.8

C(32)-C(31)-H(31)	120.1
C(30)-C(31)-C(32)	119.7(6)
C(30)-C(31)-H(31)	120.1
C(38)-C(37)-C(41)	120.1(5)
C(36)-C(37)-C(38)	119.2(5)
C(36)-C(37)-C(41)	120.6(5)
C(44)-C(45)-C(52)	123.2(6)
C(46)-C(45)-C(44)	116.8(6)
C(46)-C(45)-C(52)	120.0(5)
C(23)-C(26)-H(26A)	109.5
C(23)-C(26)-H(26B)	109.5
C(23)-C(26)-H(26C)	109.5
H(26A)-C(26)-H(26B)	109.5
H(26A)-C(26)-H(26C)	109.5
H(26B)-C(26)-H(26C)	109.5
C(64)-C(63)-C(67)	121.0(5)
C(62)-C(63)-C(64)	118.8(5)
C(62)-C(63)-C(67)	120.1(5)
C(8)-C(13)-C(16)	121.4(5)
C(8)-C(13)-C(12)	118.1(6)
C(12)-C(13)-C(16)	120.5(5)
C(61)-C(62)-H(62)	119.2
C(63)-C(62)-C(61)	121.6(5)
C(63)-C(62)-H(62)	119.2
C(35)-C(36)-H(36)	119.4
C(37)-C(36)-C(35)	121.3(5)
C(37)-C(36)-H(36)	119.4
O(4)-C(43)-C(28)	112.5(4)
O(4)-C(43)-H(43A)	109.1
O(4)-C(43)-H(43B)	109.1
C(28)-C(43)-H(43A)	109.1
C(28)-C(43)-H(43B)	109.1
H(43A)-C(43)-H(43B)	107.8
C(28)-C(29)-H(29)	118.7
C(30)-C(29)-C(28)	122.5(6)
C(30)-C(29)-H(29)	118.7

C(49)-C(48)-H(48)	118.6
C(49)-C(48)-C(47)	122.8(6)
C(47)-C(48)-H(48)	118.6
C(49)-C(50)-H(50A)	109.5
C(49)-C(50)-H(50B)	109.5
C(49)-C(50)-H(50C)	109.5
H(50A)-C(50)-H(50B)	109.5
H(50A)-C(50)-H(50C)	109.5
H(50B)-C(50)-H(50C)	109.5
C(9)-C(10)-H(10)	118.6
C(11)-C(10)-C(9)	122.7(6)
C(11)-C(10)-H(10)	118.6
C(39)-C(40)-H(40A)	109.5
C(39)-C(40)-H(40B)	109.5
C(39)-C(40)-H(40C)	109.5
H(40A)-C(40)-H(40B)	109.5
H(40A)-C(40)-H(40C)	109.5
H(40B)-C(40)-H(40C)	109.5
C(21)-C(25)-H(25A)	109.5
C(21)-C(25)-H(25B)	109.5
C(21)-C(25)-H(25C)	109.5
H(25A)-C(25)-H(25B)	109.5
H(25A)-C(25)-H(25C)	109.5
H(25B)-C(25)-H(25C)	109.5
C(65)-C(66)-H(66A)	109.5
C(65)-C(66)-H(66B)	109.5
C(65)-C(66)-H(66C)	109.5
H(66A)-C(66)-H(66B)	109.5
H(66A)-C(66)-H(66C)	109.5
H(66B)-C(66)-H(66C)	109.5
C(71)-C(78)-H(78A)	109.5
C(71)-C(78)-H(78B)	109.5
C(71)-C(78)-H(78C)	109.5
H(78A)-C(78)-H(78B)	109.5
H(78A)-C(78)-H(78C)	109.5
H(78B)-C(78)-H(78C)	109.5

C(13)-C(16)-H(16A)	109.5
C(13)-C(16)-H(16B)	109.5
C(13)-C(16)-H(16C)	109.5
H(16A)-C(16)-H(16B)	109.5
H(16A)-C(16)-H(16C)	109.5
H(16B)-C(16)-H(16C)	109.5
C(73)-C(77)-H(77A)	109.5
C(73)-C(77)-H(77B)	109.5
C(73)-C(77)-H(77C)	109.5
H(77A)-C(77)-H(77B)	109.5
H(77A)-C(77)-H(77C)	109.5
H(77B)-C(77)-H(77C)	109.5
C(10)-C(11)-C(12)	118.9(6)
C(10)-C(11)-C(15)	120.5(7)
C(12)-C(11)-C(15)	120.6(7)
C(61)-C(68)-H(68A)	109.5
C(61)-C(68)-H(68B)	109.5
C(61)-C(68)-H(68C)	109.5
H(68A)-C(68)-H(68B)	109.5
H(68A)-C(68)-H(68C)	109.5
H(68B)-C(68)-H(68C)	109.5
C(37)-C(41)-H(41A)	109.5
C(37)-C(41)-H(41B)	109.5
C(37)-C(41)-H(41C)	109.5
H(41A)-C(41)-H(41B)	109.5
H(41A)-C(41)-H(41C)	109.5
H(41B)-C(41)-H(41C)	109.5
C(63)-C(67)-H(67A)	109.5
C(63)-C(67)-H(67B)	109.5
C(63)-C(67)-H(67C)	109.5
H(67A)-C(67)-H(67B)	109.5
H(67A)-C(67)-H(67C)	109.5
H(67B)-C(67)-H(67C)	109.5
C(31)-C(30)-H(30)	120.0
C(29)-C(30)-C(31)	120.0(6)
C(29)-C(30)-H(30)	120.0

C(13)-C(12)-C(11)	121.0(6)
C(13)-C(12)-H(12)	119.5
C(11)-C(12)-H(12)	119.5
C(48)-C(47)-C(51)	120.7(6)
C(46)-C(47)-C(48)	117.7(6)
C(46)-C(47)-C(51)	121.6(6)
C(9)-C(14)-H(14A)	109.5
C(9)-C(14)-H(14B)	109.5
C(9)-C(14)-H(14C)	109.5
H(14A)-C(14)-H(14B)	109.5
H(14A)-C(14)-H(14C)	109.5
H(14B)-C(14)-H(14C)	109.5
C(35)-C(42)-H(42A)	109.5
C(35)-C(42)-H(42B)	109.5
C(35)-C(42)-H(42C)	109.5
H(42A)-C(42)-H(42B)	109.5
H(42A)-C(42)-H(42C)	109.5
H(42B)-C(42)-H(42C)	109.5
C(45)-C(46)-H(46)	118.6
C(47)-C(46)-C(45)	122.8(5)
C(47)-C(46)-H(46)	118.6
C(45)-C(52)-H(52A)	109.5
C(45)-C(52)-H(52B)	109.5
C(45)-C(52)-H(52C)	109.5
H(52A)-C(52)-H(52B)	109.5
H(52A)-C(52)-H(52C)	109.5
H(52B)-C(52)-H(52C)	109.5
C(11)-C(15)-H(15A)	109.5
C(11)-C(15)-H(15B)	109.5
C(11)-C(15)-H(15C)	109.5
H(15A)-C(15)-H(15B)	109.5
H(15A)-C(15)-H(15C)	109.5
H(15B)-C(15)-H(15C)	109.5
C(47)-C(51)-H(51A)	109.5
C(47)-C(51)-H(51B)	109.5
C(47)-C(51)-H(51C)	109.5

H(51A)-C(51)-H(51B)	109.5
H(51A)-C(51)-H(51C)	109.5
H(51B)-C(51)-H(51C)	109.5

Symmetry transformations used to generate equivalent atoms:

Table 4. Anisotropic displacement parameters ($Å^2$x 10^3) for Cain58 (**3**). The anisotropic displacement factor exponent takes the form: $-2\pi^2[h^2 a^{*2}U^{11} + ... + 2 h k a^* b^* U^{12}]$

	U^{11}	U^{22}	U^{33}	U^{23}	U^{13}	U^{12}
P(2)	12(1)	32(1)	24(1)	7(1)	4(1)	6(1)
P(3)	12(1)	32(1)	25(1)	7(1)	3(1)	4(1)
P(1)	14(1)	32(1)	24(1)	7(1)	5(1)	8(1)
O(6)	15(2)	33(2)	24(2)	6(2)	7(2)	4(2)
O(2)	10(2)	33(2)	28(2)	4(2)	1(2)	4(2)
O(1)	24(2)	35(2)	29(2)	16(2)	11(2)	12(2)
O(4)	21(2)	40(2)	39(2)	14(2)	-1(2)	6(2)
O(3)	22(2)	23(2)	51(2)	8(2)	-4(2)	0(2)
O(5)	32(2)	39(2)	27(2)	6(2)	12(2)	10(2)
C(6)	16(3)	28(3)	27(3)	6(2)	1(2)	5(2)
C(75)	20(3)	25(3)	26(3)	5(2)	2(2)	7(2)
C(58)	14(3)	31(3)	21(3)	4(2)	-4(2)	2(2)
C(57)	20(3)	27(3)	27(3)	6(2)	0(2)	3(2)
C(17)	17(3)	33(3)	25(3)	4(2)	9(2)	9(2)
C(3)	11(3)	28(3)	41(3)	7(3)	4(2)	6(2)
C(28)	13(3)	40(4)	38(3)	17(3)	10(2)	5(2)
C(1)	15(3)	22(3)	19(3)	2(2)	1(2)	6(2)
C(2)	14(3)	20(3)	29(3)	1(2)	2(2)	1(2)
C(53)	14(3)	25(3)	17(3)	-1(2)	-4(2)	2(2)
C(18)	14(3)	21(3)	26(3)	4(2)	2(2)	2(2)
C(27)	12(3)	27(3)	30(3)	11(3)	9(2)	4(2)
C(7)	25(3)	38(3)	25(3)	14(3)	10(2)	12(3)
C(60)	28(3)	33(3)	18(3)	3(2)	7(2)	7(3)
C(23)	18(3)	24(3)	27(3)	5(2)	8(2)	10(2)
C(55)	19(3)	35(3)	26(3)	2(3)	4(2)	7(2)
C(59)	30(3)	42(4)	23(3)	8(3)	9(2)	14(3)
C(39)	29(3)	20(3)	34(3)	7(2)	4(3)	5(2)
C(20)	26(3)	19(3)	27(3)	2(2)	-3(2)	-2(2)
C(19)	23(3)	17(3)	37(3)	6(2)	9(2)	5(2)
C(70)	20(3)	32(3)	20(3)	1(2)	5(2)	6(2)
C(65)	24(3)	36(3)	25(3)	0(3)	7(2)	8(3)

C(22)	14(3)	29(3)	45(4)	1(3)	6(2)	4(2)
C(32)	19(3)	31(3)	38(3)	14(3)	9(2)	6(2)
C(54)	15(3)	35(3)	23(3)	6(2)	1(2)	4(2)
C(35)	29(3)	31(3)	36(4)	6(3)	5(3)	9(3)
C(74)	20(3)	30(3)	37(3)	3(3)	6(2)	10(2)
C(72)	29(3)	27(3)	41(3)	11(3)	12(3)	7(3)
C(76)	23(3)	32(3)	40(3)	10(3)	8(3)	12(3)
C(44)	20(3)	46(4)	30(3)	12(3)	1(2)	9(3)
C(71)	24(3)	30(3)	36(3)	9(3)	11(2)	12(2)
C(34)	22(3)	19(3)	37(3)	9(3)	-3(3)	-1(2)
C(38)	24(3)	18(3)	54(4)	14(3)	11(3)	3(2)
C(69)	19(3)	29(3)	25(3)	6(2)	1(2)	3(2)
C(33)	27(3)	27(3)	34(3)	10(3)	5(3)	5(3)
C(8)	23(3)	38(3)	37(3)	23(3)	15(3)	16(3)
C(21)	24(3)	25(3)	35(3)	-3(3)	-3(3)	8(2)
C(5)	30(3)	31(3)	37(3)	15(3)	6(3)	10(3)
C(73)	24(3)	33(3)	31(3)	7(3)	11(2)	10(3)
C(56)	24(3)	37(3)	29(3)	-1(3)	-2(2)	12(3)
C(61)	29(3)	46(4)	22(3)	3(3)	6(3)	5(3)
C(9)	26(3)	44(4)	45(4)	28(3)	17(3)	12(3)
C(49)	23(3)	50(4)	27(3)	13(3)	4(2)	15(3)
C(24)	36(3)	34(3)	31(3)	2(3)	15(3)	10(3)
C(64)	29(3)	37(4)	25(3)	-4(3)	8(3)	-1(3)
C(4)	24(3)	38(4)	44(4)	18(3)	2(3)	14(3)
C(31)	30(3)	32(3)	54(4)	8(3)	6(3)	17(3)
C(37)	34(3)	20(3)	46(4)	10(3)	-8(3)	2(3)
C(45)	23(3)	71(5)	30(3)	15(3)	-1(3)	12(3)
C(26)	22(3)	45(4)	34(3)	10(3)	11(3)	7(3)
C(63)	48(4)	29(3)	28(3)	2(3)	13(3)	11(3)
C(13)	41(3)	30(3)	29(3)	10(3)	13(3)	7(3)
C(62)	35(3)	54(4)	24(3)	4(3)	3(3)	13(3)
C(36)	44(4)	29(3)	29(3)	2(3)	-1(3)	10(3)
C(43)	18(3)	51(4)	37(3)	20(3)	6(3)	10(3)
C(29)	21(3)	52(4)	66(5)	30(4)	4(3)	16(3)
C(48)	24(3)	63(4)	46(4)	27(3)	8(3)	11(3)
C(50)	26(3)	48(4)	33(3)	12(3)	2(3)	5(3)

C(10)	39(4)	57(4)	74(5)	46(4)	36(4)	30(3)
C(40)	43(4)	46(4)	29(3)	7(3)	10(3)	14(3)
C(25)	25(3)	28(3)	53(4)	-4(3)	-8(3)	8(3)
C(66)	28(3)	62(4)	35(3)	12(3)	4(3)	5(3)
C(78)	33(3)	37(4)	56(4)	17(3)	18(3)	20(3)
C(16)	41(4)	49(4)	35(4)	3(3)	1(3)	7(3)
C(77)	28(3)	43(4)	64(4)	20(3)	22(3)	12(3)
C(11)	58(4)	32(4)	57(5)	22(3)	40(4)	21(3)
C(68)	37(4)	69(5)	37(4)	9(3)	-4(3)	-2(3)
C(41)	36(4)	31(4)	68(5)	5(3)	-6(3)	7(3)
C(67)	67(5)	42(4)	42(4)	11(3)	16(3)	21(3)
C(30)	27(3)	49(4)	70(5)	16(4)	9(3)	18(3)
C(12)	62(5)	37(4)	47(4)	12(3)	29(3)	14(3)
C(47)	30(4)	98(6)	54(4)	48(4)	12(3)	19(4)
C(14)	22(3)	80(5)	50(4)	32(4)	4(3)	6(3)
C(42)	59(4)	66(5)	35(4)	5(3)	15(3)	24(4)
C(46)	24(3)	108(6)	39(4)	35(4)	0(3)	20(4)
C(52)	33(4)	78(5)	42(4)	14(4)	-8(3)	1(3)
C(15)	83(6)	58(5)	99(6)	22(5)	66(5)	31(4)
C(51)	41(5)	180(10)	126(8)	122(8)	1(5)	13(5)

Table 5. Hydrogen coordinates (x 10^4) and isotropic displacement parameters ($Å^2$x 10^3) for Cain58 (**3**).

	x	y	z	U(eq)
H(57)	2301	3245	7196	30
H(17A)	5753	6176	7490	29
H(17B)	4438	6387	7204	29
H(3)	5661	5343	8231	32
H(7A)	1456	6722	9021	33
H(7B)	553	5850	8529	33
H(55)	4174	4424	5953	33
H(59A)	2257	5998	5818	36
H(59B)	3755	6400	6134	36
H(20)	8292	8268	9527	31
H(22)	9896	7902	7983	36
H(74)	-4351	3918	7634	35
H(72)	-3005	1736	7949	38
H(76A)	-2081	5355	7474	46
H(76B)	-3234	4838	6929	46
H(76C)	-1738	4835	6849	46
H(38)	8558	9899	8047	37
H(69A)	858	3931	7756	30
H(69B)	498	4868	7632	30
H(33A)	3275	9006	8402	35
H(33B)	4085	8676	7868	35
H(5)	2706	4972	9245	37
H(56)	3886	3296	6534	37
H(24A)	5172	8225	8906	50
H(24B)	6053	8294	9530	50
H(24C)	5276	7292	9128	50
H(64)	6165	7777	4778	40
H(4)	4698	4704	8980	39
H(31)	2269	10386	7929	45
H(26A)	7839	6800	7018	50

H(26B)	8448	7884	7033	50
H(26C)	6889	7491	7007	50
H(62)	2411	6864	3950	46
H(36)	7584	10906	9671	42
H(43A)	79	6969	6217	40
H(43B)	-343	7702	5861	40
H(29)	-187	9168	6394	52
H(48)	3697	9904	5118	50
H(50A)	3526	9131	6323	54
H(50B)	4621	9300	5879	54
H(50C)	3944	8260	5943	54
H(10)	-2821	6199	9855	57
H(40A)	5729	8900	7268	58
H(40B)	7040	9595	7152	58
H(40C)	5709	9934	7192	58
H(25A)	10940	9073	9323	57
H(25B)	11303	8203	8913	57
H(25C)	10654	8082	9515	57
H(66A)	5768	6308	5843	63
H(66B)	6681	7255	5730	63
H(66C)	6711	6269	5326	63
H(78A)	-566	1933	7048	58
H(78B)	-1113	1416	7565	58
H(78C)	51	2356	7740	58
H(16A)	2494	6130	10390	65
H(16B)	2509	7081	10850	65
H(16C)	2702	7089	10159	65
H(77A)	-4928	2645	8550	64
H(77B)	-5359	1742	8008	64
H(77C)	-5818	2669	7949	64
H(68A)	1563	4830	4455	77
H(68B)	938	5526	4140	77
H(68C)	1021	5620	4856	77
H(41A)	10190	10348	8874	70
H(41B)	9671	10172	9495	70
H(41C)	10043	11209	9373	70

H(67A)	4138	8645	4054	72
H(67B)	4258	7857	3496	72
H(67C)	5539	8434	3960	72
H(30)	588	10411	7197	56
H(12)	457	7323	11053	57
H(14A)	-1779	5697	8641	74
H(14B)	-2776	5009	8951	74
H(14C)	-1401	4794	8806	74
H(42A)	4591	11201	9194	78
H(42B)	5447	11112	9787	78
H(42C)	4347	10224	9404	78
H(46)	223	8593	4130	65
H(52A)	-475	6684	4874	81
H(52B)	-734	6927	4225	81
H(52C)	-1353	7400	4785	81
H(15A)	-2986	7231	10746	111
H(15B)	-1662	7889	11147	111
H(15C)	-2277	6879	11277	111
H(51A)	1694	10542	4298	160
H(51B)	1896	9768	3751	160
H(51C)	3136	10395	4223	160

X-RAY OF 6

Table 1. Crystal data and structure refinement for cain100_0m_b (**6**).

Identification code	BIII	
Empirical formula	C20 H13 F12 O2 P	
Formula weight	544.27	
Temperature	100.0 K	
Wavelength	1.54178 Å	
Crystal system	Monoclinic	
Space group	P 21	
Unit cell dimensions	a = 6.4519(4) Å	α= 90°.
	b = 14.3030(9) Å	β= 92.203(4)°.
	c = 11.4008(6) Å	γ = 90°.
Volume	1051.31(11) Å^3	
Z	2	
Density (calculated)	1.719 Mg/m^3	
Absorption coefficient	2.334 mm^{-1}	
F(000)	544	
Crystal size	0.29 x 0.26 x 0.23 mm^3	
Theta range for data collection	3.880 to 70.023°.	
Index ranges	-7<=h<=7, -17<=k<=17, -13<=l<=13	
Reflections collected	6831	
Independent reflections	3721 [R(int) = 0.0264]	
Completeness to theta = 67.679°	97.6 %	
Absorption correction	None	
Max. and min. transmission	0.5220 and 0.4062	
Refinement method	Full-matrix least-squares on F^2	
Data / restraints / parameters	3721 / 1 / 333	
Goodness-of-fit on F^2	1.086	
Final R indices [I>2sigma(I)]	R1 = 0.0508, wR2 = 0.1294	
R indices (all data)	R1 = 0.0583, wR2 = 0.1345	
Absolute structure parameter	0.14(3)	
Extinction coefficient	n/a	
Largest diff. peak and hole	0.478 and -0.463 e.Å^{-3}	

Table 2. Atomic coordinates (x 10^4) and equivalent isotropic displacement parameters (Å^{2}x 10^3) for cain100_0m_b (**6**). U(eq) is defined as one third of the trace of the orthogonalized U^{ij} tensor.

	x	y	z	U(eq)
F(1)	5175(18)	6976(4)	6142(6)	112(4)
F(2)	3119(14)	8130(4)	6482(6)	84(3)
F(3)	5550(11)	7759(4)	7724(6)	62(2)
F(4)	685(14)	7966(4)	8341(5)	80(3)
F(5)	3006(9)	7381(4)	9475(4)	51(1)
F(6)	311(9)	6609(5)	9080(6)	61(2)
F(7)	334(10)	3373(5)	9067(7)	70(2)
F(8)	2999(9)	2549(5)	9391(5)	62(2)
F(9)	504(13)	2040(5)	8289(5)	74(2)
F(10)	5556(12)	2277(5)	7593(6)	71(2)
F(11)	3089(14)	1853(4)	6444(6)	84(3)
F(12)	4781(15)	3052(5)	5983(5)	84(2)
O(1)	921(18)	6450(7)	6835(9)	31(3)
O(1')	1540(40)	6529(17)	6550(20)	32(2)
O(2)	770(20)	3587(10)	6825(10)	30(2)
O(2')	1360(50)	3490(20)	6550(30)	30(2)
C(2)	1325(6)	5050(9)	5146(3)	36(1)
C(3)	3350(6)	5035(9)	4785(3)	39(1)
C(4)	3750(6)	5024(9)	3599(3)	34(1)
C(5)	2150(6)	4991(7)	2747(3)	28(1)
C(6)	141(6)	5005(8)	3115(3)	32(1)
C(7)	-280(6)	5012(8)	4298(3)	32(1)
C(8)	2618(7)	5001(9)	1462(3)	37(1)
C(9)	2599(13)	3261(6)	7506(7)	31(2)
C(10)	4030(20)	2603(7)	6873(8)	53(2)
C(11)	1621(15)	2792(6)	8575(8)	36(2)
C(12)	2871(5)	4995(7)	7548(3)	20(1)
C(13)	3709(12)	4155(5)	7903(6)	22(2)
C(14)	5524(12)	4142(5)	8651(6)	24(2)
C(15)	6415(6)	4979(8)	8995(3)	30(1)
C(16)	5554(13)	5817(6)	8658(7)	30(2)

C(17)	3790(12)	5833(5)	7960(6)	23(1)
C(18)	2662(14)	6713(6)	7561(6)	32(2)
C(19)	1655(14)	7187(6)	8613(7)	33(2)
C(20)	4130(20)	7403(7)	6970(7)	55(3)
P(1')	505(9)	4798(5)	6673(5)	22(1)
P(1)	496(6)	5280(3)	6649(3)	22(1)
C(1)	-110(30)	2882(12)	5942(15)	65(4)
C(1')	-20(30)	7134(15)	6016(16)	23(1)

Table 3. Bond lengths [Å] and angles [°] for cain100_0m_b (**6**).

F(1)-C(20)	1.328(12)
F(2)-C(20)	1.337(12)
F(3)-C(20)	1.334(13)
F(4)-C(19)	1.309(10)
F(5)-C(19)	1.318(9)
F(6)-C(19)	1.325(12)
F(7)-C(11)	1.316(12)
F(8)-C(11)	1.308(9)
F(9)-C(11)	1.329(10)
F(10)-C(10)	1.341(13)
F(11)-C(10)	1.317(11)
F(12)-C(10)	1.310(12)
O(1)-C(18)	1.421(13)
O(1')-C(18)	1.37(2)
O(1')-P(1)	1.91(2)
O(1')-C(1')	1.44(3)
O(2)-C(9)	1.463(16)
O(2)-P(1')	1.749(16)
O(2)-C(1)	1.520(19)
O(2')-C(9)	1.36(3)
C(2)-C(3)	1.385(6)
C(2)-C(7)	1.390(5)
C(2)-P(1')	1.874(7)
C(2)-P(1)	1.844(6)
C(3)-H(3)	0.9500
C(3)-C(4)	1.387(5)
C(4)-H(4)	0.9500
C(4)-C(5)	1.391(6)
C(5)-C(6)	1.378(6)
C(5)-C(8)	1.508(5)
C(6)-H(6)	0.9500
C(6)-C(7)	1.385(5)
C(7)-H(7)	0.9500
C(8)-H(8A)	0.9800

C(8)-H(8B)	0.9800
C(8)-H(8C)	0.9800
C(9)-C(10)	1.520(14)
C(9)-C(11)	1.548(12)
C(9)-C(13)	1.526(10)
C(12)-C(13)	1.372(11)
C(12)-C(17)	1.410(11)
C(12)-P(1')	1.814(7)
C(12)-P(1)	1.856(6)
C(13)-C(14)	1.422(11)
C(14)-H(14)	0.9500
C(14)-C(15)	1.379(13)
C(15)-H(15)	0.9500
C(15)-C(16)	1.370(13)
C(16)-H(16)	0.9500
C(16)-C(17)	1.364(11)
C(17)-C(18)	1.515(10)
C(18)-C(19)	1.542(11)
C(18)-C(20)	1.541(14)
C(1)-H(1A)	0.9800
C(1)-H(1B)	0.9800
C(1)-H(1C)	0.9800
C(1')-H(1'A)	0.9800
C(1')-H(1'B)	0.9800
C(1')-H(1'C)	0.9800
C(18)-O(1')-P(1)	107.8(15)
C(18)-O(1')-C(1')	125(2)
C(1')-O(1')-P(1)	110.2(16)
C(9)-O(2)-P(1')	116.2(9)
C(9)-O(2)-C(1)	114.5(11)
C(1)-O(2)-P(1')	124.0(11)
C(3)-C(2)-C(7)	118.6(4)
C(3)-C(2)-P(1')	125.1(4)
C(3)-C(2)-P(1)	126.1(4)
C(7)-C(2)-P(1')	114.1(4)

C(7)-C(2)-P(1)	114.7(4)
C(2)-C(3)-H(3)	119.9
C(2)-C(3)-C(4)	120.2(4)
C(4)-C(3)-H(3)	119.9
C(3)-C(4)-H(4)	119.3
C(3)-C(4)-C(5)	121.4(4)
C(5)-C(4)-H(4)	119.3
C(4)-C(5)-C(8)	120.5(4)
C(6)-C(5)-C(4)	117.9(3)
C(6)-C(5)-C(8)	121.5(4)
C(5)-C(6)-H(6)	119.4
C(5)-C(6)-C(7)	121.2(4)
C(7)-C(6)-H(6)	119.4
C(2)-C(7)-H(7)	119.7
C(6)-C(7)-C(2)	120.6(4)
C(6)-C(7)-H(7)	119.7
C(5)-C(8)-H(8A)	109.5
C(5)-C(8)-H(8B)	109.5
C(5)-C(8)-H(8C)	109.5
H(8A)-C(8)-H(8B)	109.5
H(8A)-C(8)-H(8C)	109.5
H(8B)-C(8)-H(8C)	109.5
O(2)-C(9)-C(10)	116.0(8)
O(2)-C(9)-C(11)	102.2(8)
O(2)-C(9)-C(13)	104.5(8)
O(2')-C(9)-C(10)	96.9(13)
O(2')-C(9)-C(11)	119.3(15)
O(2')-C(9)-C(13)	106.7(16)
C(10)-C(9)-C(11)	112.2(7)
C(10)-C(9)-C(13)	111.9(8)
C(13)-C(9)-C(11)	109.3(6)
F(10)-C(10)-C(9)	111.7(7)
F(11)-C(10)-F(10)	105.2(8)
F(11)-C(10)-C(9)	113.7(10)
F(12)-C(10)-F(10)	111.1(11)
F(12)-C(10)-F(11)	106.8(8)

F(12)-C(10)-C(9)	108.3(8)
F(7)-C(11)-F(9)	105.7(9)
F(7)-C(11)-C(9)	110.1(7)
F(8)-C(11)-F(7)	106.7(8)
F(8)-C(11)-F(9)	107.9(8)
F(8)-C(11)-C(9)	112.9(8)
F(9)-C(11)-C(9)	113.0(7)
C(13)-C(12)-C(17)	119.4(3)
C(13)-C(12)-P(1')	109.9(6)
C(13)-C(12)-P(1)	131.5(6)
C(17)-C(12)-P(1')	130.5(7)
C(17)-C(12)-P(1)	109.0(6)
C(12)-C(13)-C(9)	118.1(7)
C(12)-C(13)-C(14)	119.6(6)
C(14)-C(13)-C(9)	122.3(7)
C(13)-C(14)-H(14)	120.5
C(15)-C(14)-C(13)	119.0(7)
C(15)-C(14)-H(14)	120.5
C(14)-C(15)-H(15)	119.3
C(16)-C(15)-C(14)	121.3(4)
C(16)-C(15)-H(15)	119.3
C(15)-C(16)-H(16)	120.0
C(17)-C(16)-C(15)	119.9(8)
C(17)-C(16)-H(16)	120.0
C(12)-C(17)-C(18)	114.6(6)
C(16)-C(17)-C(12)	120.7(7)
C(16)-C(17)-C(18)	124.7(7)
O(1)-C(18)-C(17)	108.3(7)
O(1)-C(18)-C(19)	102.8(8)
O(1)-C(18)-C(20)	113.6(8)
O(1')-C(18)-C(17)	109.1(12)
O(1')-C(18)-C(19)	121.1(12)
O(1')-C(18)-C(20)	94.0(11)
C(17)-C(18)-C(19)	110.1(6)
C(17)-C(18)-C(20)	111.5(8)
C(20)-C(18)-C(19)	110.1(7)

F(4)-C(19)-F(5)	107.1(7)
F(4)-C(19)-F(6)	108.2(9)
F(4)-C(19)-C(18)	113.7(7)
F(5)-C(19)-F(6)	104.8(8)
F(5)-C(19)-C(18)	112.7(7)
F(6)-C(19)-C(18)	109.9(7)
F(1)-C(20)-F(2)	108.4(8)
F(1)-C(20)-F(3)	106.2(12)
F(1)-C(20)-C(18)	110.8(8)
F(2)-C(20)-C(18)	112.4(11)
F(3)-C(20)-F(2)	106.4(8)
F(3)-C(20)-C(18)	112.4(7)
O(2)-P(1')-C(2)	104.6(7)
O(2)-P(1')-C(12)	91.3(6)
C(12)-P(1')-C(2)	102.7(4)
C(2)-P(1)-O(1')	89.7(8)
C(2)-P(1)-C(12)	102.2(3)
C(12)-P(1)-O(1')	87.3(8)
O(2)-C(1)-H(1A)	109.5
O(2)-C(1)-H(1B)	109.5
O(2)-C(1)-H(1C)	109.5
H(1A)-C(1)-H(1B)	109.5
H(1A)-C(1)-H(1C)	109.5
H(1B)-C(1)-H(1C)	109.5
O(1')-C(1')-H(1'A)	109.5
O(1')-C(1')-H(1'B)	109.5
O(1')-C(1')-H(1'C)	109.5
H(1'A)-C(1')-H(1'B)	109.5
H(1'A)-C(1')-H(1'C)	109.5
H(1'B)-C(1')-H(1'C)	109.5

Symmetry transformations used to generate equivalent atoms:

Table 4. Anisotropic displacement parameters ($Å^2 x 10^3$) for cain100_0m_b (**6**). The anisotropic displacement factor exponent takes the form: $-2\pi^2[h^2 a^{*2}U^{11} + ... + 2 h k a^* b^* U^{12}]$

	U^{11}	U^{22}	U^{33}	U^{23}	U^{13}	U^{12}
F(1)	231(10)	35(4)	79(4)	18(3)	99(6)	16(4)
F(2)	171(9)	20(3)	58(4)	11(3)	-26(4)	10(4)
F(3)	63(4)	40(3)	85(4)	17(3)	12(3)	-12(3)
F(4)	139(7)	49(4)	51(3)	-18(3)	-19(4)	63(4)
F(5)	39(3)	80(4)	33(2)	-23(2)	-6(2)	1(3)
F(6)	37(3)	54(4)	94(5)	-33(3)	25(3)	-3(3)
F(7)	43(4)	64(4)	105(5)	38(4)	32(4)	6(3)
F(8)	38(3)	88(5)	60(3)	52(3)	-15(2)	-10(3)
F(9)	108(6)	62(4)	53(3)	20(3)	-8(3)	-54(4)
F(10)	70(5)	54(4)	88(4)	-31(3)	0(3)	23(3)
F(11)	165(9)	31(3)	53(3)	-8(3)	-23(4)	-20(4)
F(12)	153(7)	46(4)	57(3)	-10(3)	49(4)	-1(3)
O(1)	34(6)	26(5)	32(5)	-13(4)	-14(4)	13(4)
O(1')	40(5)	27(4)	29(3)	-8(3)	-10(3)	4(3)
O(2)	37(7)	26(4)	27(6)	5(4)	-10(4)	-8(5)
O(2')	37(7)	26(4)	27(6)	5(4)	-10(4)	-8(5)
C(2)	25(2)	63(4)	18(2)	4(4)	-2(1)	-3(4)
C(3)	25(2)	69(4)	21(2)	-1(4)	-4(2)	5(5)
C(4)	26(2)	47(3)	28(2)	0(4)	4(2)	4(4)
C(5)	40(2)	23(2)	21(2)	1(3)	1(2)	-1(4)
C(6)	35(2)	36(2)	23(2)	-10(4)	-8(2)	3(4)
C(7)	23(2)	48(3)	24(2)	-1(4)	-3(1)	-6(4)
C(8)	46(3)	42(3)	24(2)	3(4)	2(2)	-1(5)
C(9)	38(5)	21(4)	33(3)	4(3)	-10(3)	-10(3)
C(10)	85(8)	24(5)	50(5)	-8(4)	6(4)	1(5)
C(11)	31(5)	29(5)	47(4)	8(3)	-9(3)	-9(3)
C(12)	21(2)	23(2)	16(2)	0(3)	2(1)	-3(4)
C(13)	26(4)	22(4)	19(3)	0(2)	4(2)	1(3)
C(14)	26(4)	22(4)	23(3)	3(3)	-2(3)	5(3)
C(15)	26(2)	37(2)	25(2)	-2(4)	-9(1)	15(4)
C(16)	27(4)	28(4)	33(4)	-3(3)	-3(3)	-3(3)

C(17)	26(4)	18(4)	24(3)	-2(2)	-2(2)	4(3)
C(18)	40(5)	27(4)	29(3)	-8(3)	-10(3)	4(3)
C(19)	29(4)	32(5)	37(4)	-17(3)	-14(3)	7(3)
C(20)	107(9)	27(5)	32(4)	4(4)	6(4)	12(5)
P(1')	19(1)	26(1)	19(1)	-5(2)	-2(1)	2(2)
P(1)	19(1)	26(1)	19(1)	-5(2)	-2(1)	2(2)
C(1)	61(8)	57(7)	76(8)	-12(6)	-20(6)	-31(6)
C(1')	26(4)	18(4)	24(3)	-2(2)	-2(2)	4(3)

Table 5. Hydrogen coordinates (x 10^4) and isotropic displacement parameters ($Å^2$x 10^3) for cain100_0m_b (**6**).

	x	y	z	U(eq)
H(3)	4468	5031	5352	46
H(4)	5146	5040	3363	40
H(6)	-977	5009	2548	38
H(7)	-1676	4990	4531	38
H(8A)	2789	5648	1201	56
H(8B)	1470	4709	1008	56
H(8C)	3899	4651	1341	56
H(14)	6113	3566	8910	29
H(15)	7650	4975	9476	36
H(16)	6185	6387	8910	35
H(1A)	665	2914	5221	98
H(1B)	-1575	3026	5761	98
H(1C)	3	2251	6274	98
H(1'A)	-593	7538	6618	34
H(1'B)	-1137	6754	5653	34
H(1'C)	604	7521	5415	34
